# Probabilistic projections of global wind and solar power growth based on historical national experience

**DOI:** 10.1038/s41560-026-02021-w

**Published:** 2026-04-14

**Authors:** Avi Jakhmola, Jessica Jewell, Vadim Vinichenko, Aleh Cherp

**Affiliations:** 1 Division of Physical Resource Theory, Department of Space, Earth and Environment, Chalmers University of Technology, Gothenburg, Sweden; 2 Centre for Climate and Energy Transformations and Department of Geography, University of Bergen, Bergen, Norway; 3 Advancing Systems Analysis, International Institute for Applied Systems Analysis, Laxenburg, Austria; 4 Department of Environmental Science and Policy, Central European University University, Vienna, Austria; 5 International Institute for Industrial Environmental Economics, Lund University, Lund, Sweden

**Keywords:** Climate-change mitigation, Energy supply and demand, Political economy of energy

## Abstract

Despite the recent surge of wind and solar power, both technologies need to accelerate to meet climate goals. Yet, there are no robust methods to assess the likelihood of such acceleration. Here we show that renewable energy deployment follows a recurring pattern across countries with prolonged periods of relatively steady growth punctuated by growth pulses. Based on this insight and on observed growth trajectories in early adopting countries, we develop a probabilistic model (PROLONG) for projecting global wind and solar power deployment. In our central projections, both wind and solar power grow similarly to Intergovernmental Panel on Climate Change 2 °C-compatible pathways and faster than in current policy scenarios. The COP28 pledge to triple renewables by 2030 is near the 95th percentile of our projections and requires that the growth of wind and solar photovoltaics in major economies accelerate by 1.4–3 times and 2–5 times, respectively. PROLONG can be adopted for data-driven projections of other policy-dependent energy technologies.

## Abstract (web summary)

PROLONG, a data-driven probabilistic model of technology growth, projects wind and solar expansion consistent with 2 °C pathways and faster than current policy scenarios. The 1.5 °C pathway lies beyond the 95th percentile of projections and meeting this target would require major effort.

## Main

The meteoric rise of wind and solar power^1,2^, impressive as it is, must still accelerate to meet climate change mitigation targets^2–4^. Responding to this challenge, over 130 countries have joined the global pledge to triple renewable energy capacity by 2030^5^. Yet, whether this goal will be met, and how growth will unfold beyond 2030, remains uncertain^1^. Despite the importance of these questions for climate and energy policy, scholars disagree on the likely speed of future wind and solar expansion^6–11^.

This disagreement reflects the lack of a systematic method for projecting renewable power growth. Most existing projections^12–14^ rely on integrated assessment models (IAMs) or energy system optimization models (ESOMs), which remain the primary tools for exploring long-term climate and energy scenarios. These models excel at identifying plausible energy system configurations that balance supply and demand under geophysical, economic, infrastructural and policy constraints (for example, carbon pricing or emissions limits). However, they struggle to capture innovation, inertia, path dependence and feedbacks in sociotechnical and political systems^15,16^—factors that may cause renewable energy growth to diverge from cost-optimal trajectories^17–19^. In response, alternative approaches have emerged, where future growth is projected on the basis of historical energy transitions^17,20–23^ or recent deployment trends^6,9–11,24^. Rather than modelling causal mechanisms, these data-driven projections extrapolate empirically observed growth trajectories, which implicitly reflect the combined influence of technological, economic and sociopolitical drivers and barriers.

Such extrapolations are challenging, however, because renewables growth is nonlinear: like other new technologies, it initially accelerates before slowing along an S-shaped trajectory^25,26^. Because wind and solar power are still accelerating globally, projections for their future hinge on assumptions about how long this acceleration will last and how quickly it will give way to slow-down. These assumptions, in turn, depend on the balance of positive feedbacks—such as those between deployment, technological learning and cost decline^7,9^—and negative feedbacks from conflicting land uses^27^, local opposition^28,29^, political backlash^30^ and grid integration challenges^31^. Despite growing qualitative understanding of these mechanisms, few quantitative models capture and extrapolate their combined effects on deployment speeds.

Here, we develop PROLONG: a data-driven model of global wind and solar power growth that draws on national deployment trajectories and recurring growth phases. These include the formative phase with typically erratic growth, the initial acceleration phase and prolonged periods of steady expansion interspersed with growth pulses often triggered by policy change. Using patterns from early adopters, PROLONG generates global probabilistic projections through 2050. In our central projection, onshore wind generates 13.4% (interquartile range (IQR) 12.6–14.3%) of global electricity by 2030 and 26% (21−34%) by 2050, while solar photovoltaics (PV) reaches 12.3% (10.6–14.8%) by 2030 and 21% (15–29%) by 2050. These estimates exceed current policy scenarios and closely track 2 °C-compatible pathways assessed by the Intergovernmental Panel on Climate Change (IPCC), however they fall short of many 1.5 °C-consistent trajectories. The COP28 pledge to triple renewable capacity by 2030 lies near the 95th percentile of our projections and would require accelerating recent wind growth by 1.4–3 times and solar PV growth by 2–5 times across different regions. Beyond informing policy debates around feasibility and ambition, PROLONG offers a promising framework for projecting the growth of other policy-dependent energy technologies based on empirical evidence.

## Phases of technology growth and diffusion

**Fig. 1:**
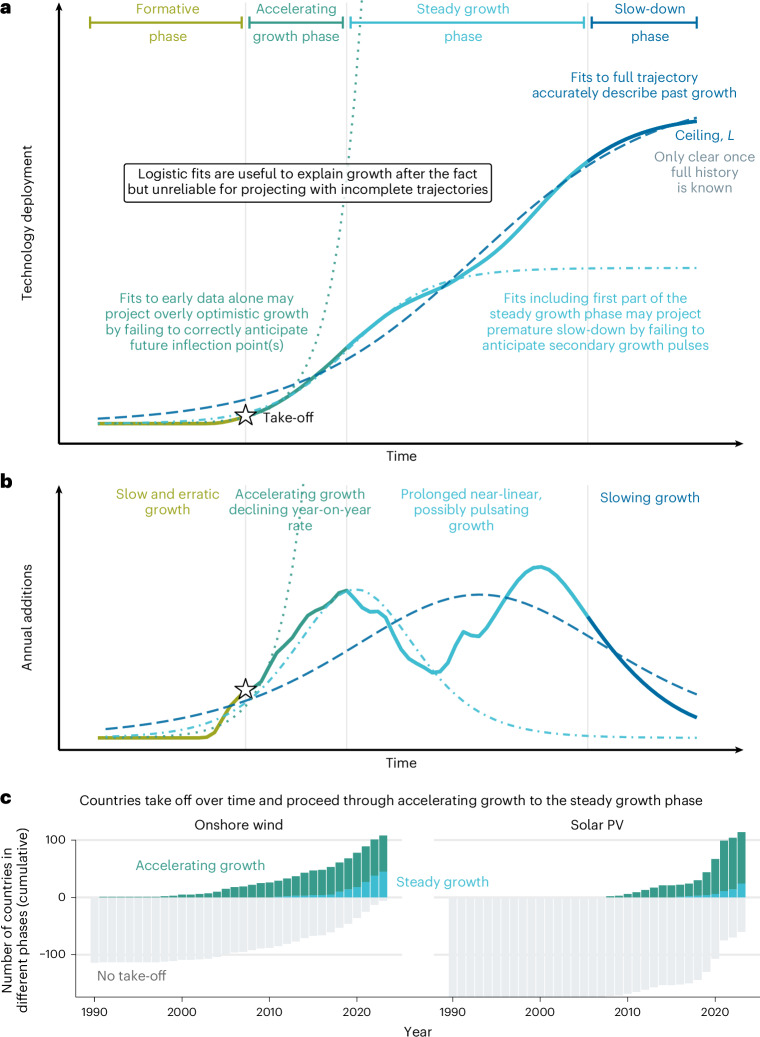
Phases of policy-driven technology growth and diffusion of onshore wind and solar power. **a**,**b**, Technology deployment follows an S-curve with distinct phases, each shaped by different mechanisms, making it challenging to anticipate later growth from earlier evidence^33^. Policy-driven technologies often include a prolonged steady growth phase between initial acceleration and eventual slow-down, which may include distinct growth ‘pulses’. **c**, Over time, more countries experience onshore wind and solar take-off and proceed through initial acceleration to the steady growth phase. Source data

Growth of new technologies is uneven: it is driven by different mechanisms and exhibits different patterns at different phases^25,26,32,33^ (Fig. 1 and Supplementary Note 1). At the first, formative phase, technologies are used in niche applications and grow slowly and erratically, constrained by high costs, uncertainty and frequent failures^18,34–37^. During this phase, the system-wide impact of technologies remains small, and their growth is unpredictable, bearing little resemblance to the more regular patterns of later stages. The formative phase is especially pronounced at the national level for policy-driven technologies such as nuclear, wind and solar power, where it involves not only technological innovation but also the formation of institutions and actor networks^35,38^. For successful technologies, the formative phase ends in ‘take-off’ marking the start of consistent acceleration^37,39^. Most prior studies have defined take-off through semi-intuitive deployment thresholds—such as absolute level^35,40^, shares of the final market^25,34,37^ and shares of the total electricity supply^18,23^—that have not been empirically validated.

**Extended Data Fig. 1:**
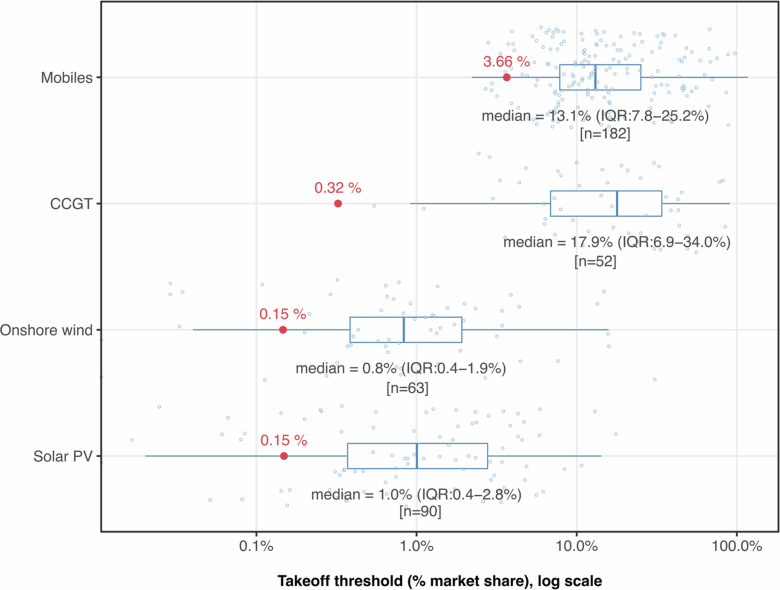
National and global takeoff thresholds for selected technologies. Boxplots show national takeoff thresholds – defined as the market share at which consistent growth begins – for solar PV, onshore wind, CCGTs, and mobile phones, based on empirical data from individual countries (faint blue dots). The boxes show the IQR, with the median marked by a solid line and whiskers extending from the IQR range to the non-outlier minimum and maximum. Red dots indicate global takeoff thresholds. Note: horizontal axis is logarithmic. Source data

**Extended Data Fig. 2:**
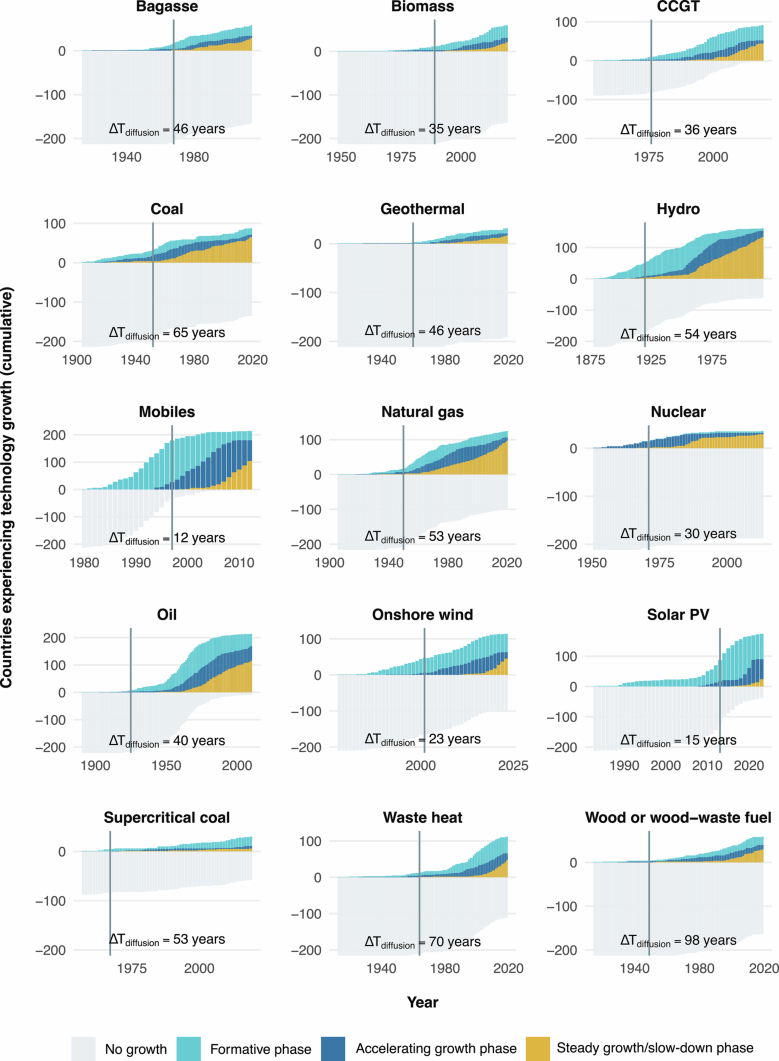
Diffusion of technologies across countries and growth phases. Cumulative number of countries in each growth phase for 14 electricity technologies and mobile telephones. Bars show the number of countries with no deployment (gray), in the formative phase (light blue), in the accelerating growth phase (dark blue), and in the steady growth or slowdown phase (yellow). The vertical gray line marks the global takeoff year. Diffusion duration indicates the time span between the 10th and 90th percentile of countries reaching takeoff. Source data

We use a Bayesian change-point estimator to empirically identify take-off years—defined as the point when a technology transitions from erratic to consistent growth (Methods). Nationally, take-off occurs at median electricity generation shares of 0.8% (IQR 0.4–1.9%) for onshore wind and 1% (0.4–2.8%) for solar PV (Extended Data Fig. 1 and Extended Data Table 1), consistent with ref. ^18^. Globally, onshore wind and solar PV took off in 1999 and 2010, respectively, when their shares in global electricity generation first exceeded 0.15%. As with other technologies, solar and wind power take off at different times across countries^18^, driven by policy and technology diffusion^25,35,41^ (Fig. 1 and Extended Data Fig. 2). We find that, by 2023, take-off had occurred in 63 countries for onshore wind and 90 for solar PV, expanding and updating the estimate in ref. ^18^, which diagnosed take-off in 37 (wind) and 31 (solar) of the 60 largest countries based on 2018–2019 data (Extended Data Table 1). The projected global diffusion duration—measured as the time it takes for the cumulative number of countries reaching take-off to increase from 10% to 90% of its estimated maximum—is 23 years for wind and 15 years for solar (Methods; Supplementary Note 1). This is faster than historical cross-country diffusion rates for other energy technologies (Extended Data Fig. 2), probably reflecting renewables’ modular design and globalized supply chains and helping to explain the prevailing optimism about their rapid expansion.

**Fig. 2:**
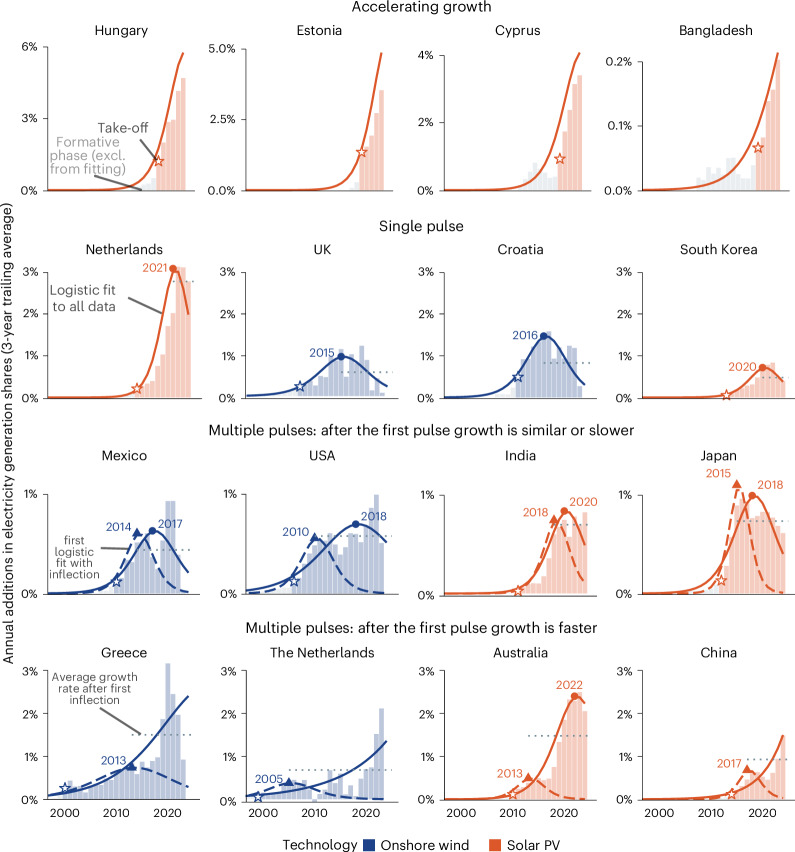
Patterns of onshore wind and solar power growth in selected countries. Bars show 3-year trailing averages for annual additions in each technology’s share in electricity generation. Grey bars show formative phase observations excluded from curve fitting. Stars mark take-off years. Triangles and circles indicate diagnosed growth peaks based on logistic fits to the full time series (all panels) or truncated time series (third and fourth rows). Solid and dashed lines show logistic fits to the full and truncated series, respectively. Horizontal dashed lines in the second, third and fourth rows indicate the average post-peak growth rate. Countries are grouped by growth patterns: continued acceleration (top row), single peak (second row) and multiple pulses (third and fourth rows). excl., excluded. Source data

**Extended Data Fig. 3:**
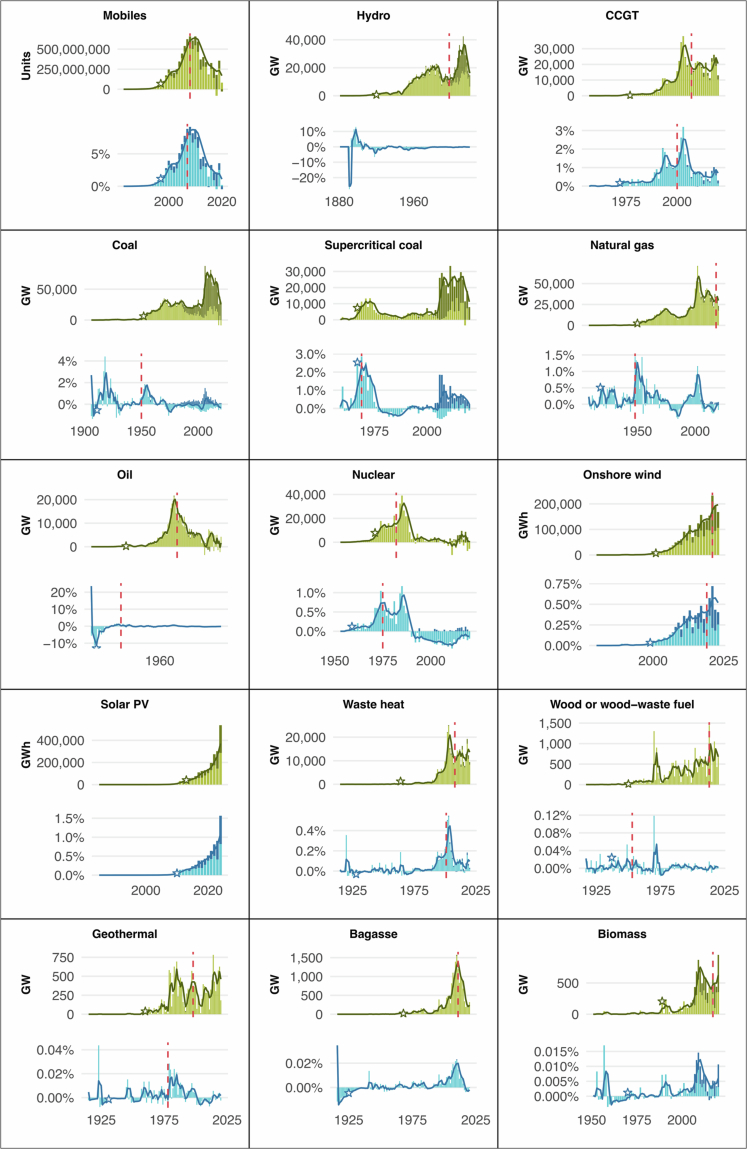
Global growth of selected technologies. Annual global additions for each technology shown in absolute units (green bars) and as share of total electricity generation, installed capacity, or other market (blue bars), with darker shares indicating contributions from China. Solid lines show three-year trailing averages; stars mark takeoff years and vertical red dashed lines mark inflection points of logistic fits to full time series. Source data

Following take-off, technologies typically enter the accelerating growth phase, driven by positive feedbacks from technological learning, economies of scale, and political support^42^. As deployment expands, however, new barriers emerge—such as land and resource constraints, system integration challenges and sociopolitical resistance—that gradually slow growth^27–31^. Growth peaks when these barriers balance the drivers, marking the end of acceleration^18,33^. Mainstream diffusion theory posits that this must be followed by an immediate slow-down phase, as barriers continue to intensify and the technology approaches market saturation^25,32^. Although this S-shaped pattern (and corresponding inverted U-shape for annual additions) is well documented for the long-term global diffusion of historical technologies (Supplementary Fig. 1 and Extended Data Fig. 3), it offers limited guidance for the medium-term growth of policy-driven technologies such as wind and solar. Their expansion can be rapidly altered by policy shifts—such as changes in subsidies, market rules, land-use regulation or state-backed infrastructure investment^33,43^. Apparent slow-downs may thus be temporary, followed by renewed growth spurts or ‘pulses’, as observed in many national trajectories (Fig. 2 and Supplementary Note 2).

Global inflection points are detectable for onshore wind but not for solar, where growth continues to accelerate (Extended Data Fig. 3; Methods). At the national level, however, inflection points are observed for both technologies in many countries (Fig. 2). In line with our expectations, some countries experience persistent slow-down after peak growth, while others re-accelerate through additional ‘pulses’. This pulsating pattern aggregates into relatively steady growth at ‘cruising speed’—an empirical regularity that has been identified in prior studies^20^, used to benchmark scenarios^18,19,23,34^ and project future deployment^20,44^. Although not formalized in mainstream diffusion or transition theories, we propose that this steady growth phase^33^, situated between initially accelerating growth and slow-down (Fig. 1), is a frequent feature of policy-driven technologies that should be explicitly considered in projection models.

## Probabilistic models for global wind and solar growth

Scholars have long sought mathematical models to represent the nonlinear dynamics of technology growth. The most widely used model—the logistic growth function—offers a parsimonious three-parameter characterization of the typical S-shaped trajectory, capturing both initial acceleration and eventual saturation^11,17,18,24,25^. While logistic functions fitted to growth trajectories have formed the basis of a rich literature on technology diffusion^17,25,26,45^ (Fig. 1 and Supplementary Note 2), they proved much less suitable for extrapolating future growth from partial time series^6,11,18,24,46–48^. As Fig. 1 illustrates, extrapolations from early phases may miss upcoming inflection points and overestimate deployment, while post-inflection fits may underestimate growth by failing to anticipate secondary pulses (Fig. 2 and Supplementary Note 2).

**Extended Data Fig. 4:**
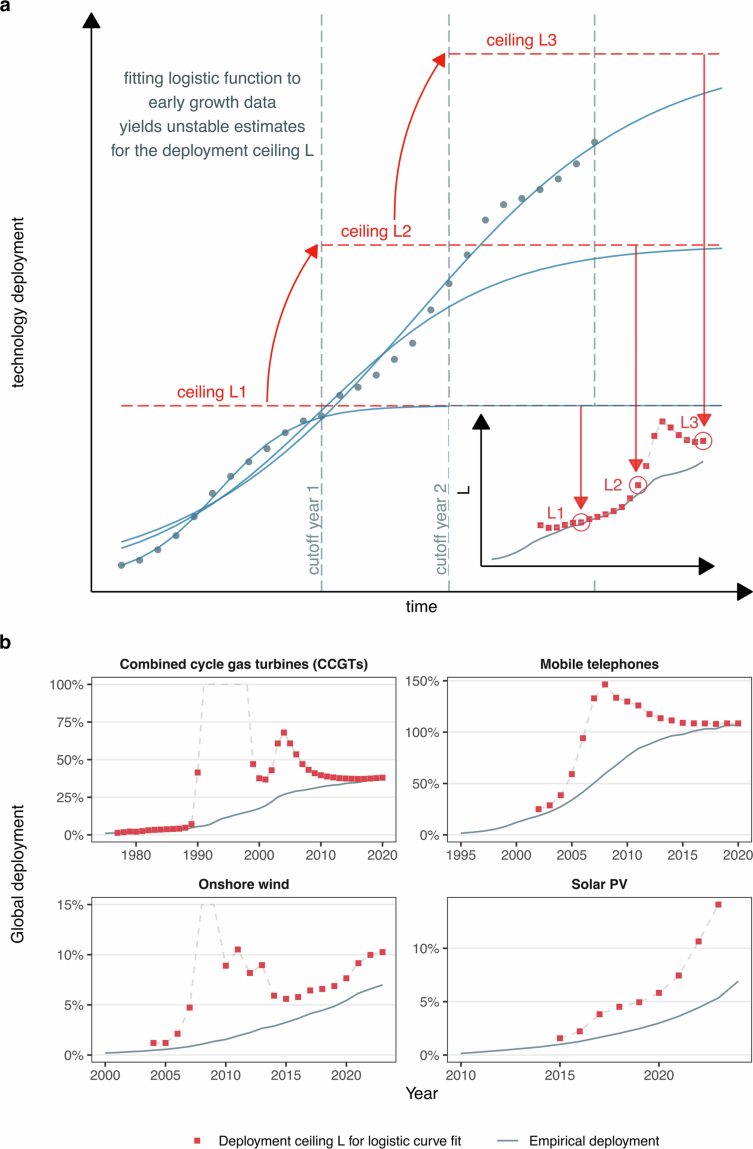
Challenges of estimating deployment ceilings from early-stage data (the ‘moving L’ problem). (**a**) Logistic functions fit to incomplete deployment data yield unstable estimates of the technology deployment ceilings, *L*, which shift as new data become available. (**b**) Red squares show changing estimates of *L* from logistic fits to truncated empirical deployment data (gray lines). Source data

Mathematically, this implies that the parameters of a logistic function that best fit the final S-curve of a technology’s diffusion cannot be reliably estimated from observing early data. This limitation is especially pronounced for the asymptote (the projected saturation level) *L*, which often shifts substantially as new observations become available (Extended Data Fig. 4). Previous studies have addressed this challenge by fixing *L* exogenously^10,49^ or by using alternative growth models^6,20,47,48^. However, these strategies do not resolve the core difficulty: the mechanisms of late-stage growth cannot be inferred from early-stage observations (Fig. 1 and Supplementary Note 2). This presents a particular challenge for projecting global wind and solar deployment, as both technologies are currently in relatively early phases of diffusion (Extended Data Figs. 2 and 3). Consequently, past projections have either under- or overestimated deployment^24,50^.

To address these limitations, we develop PROLONG (probabilistic model of technology growth), a framework for generating data-driven probabilistic estimates of global wind and solar power growth. PROLONG formalizes three stylized insights about renewable energy diffusion over time and space (Table 1).

**Table 1:** Three stylized insights about technology growth and diffusion and their implementation in PROLONG

**Insight**	Growth follows distinct phases	Policy-driven technologies often show prolonged steady growth composed of several pulses	Growth timing varies across countries, but its mechanisms remain broadly similar
**Implementation**	Segmenting observations by phase allows the estimation of meaningful growth parameters.	Bilogistic functions capture this pattern better than single-inflection S-curves.	Early adopters can inform assumptions about growth in later adopters.

**Extended Data Fig. 5:**
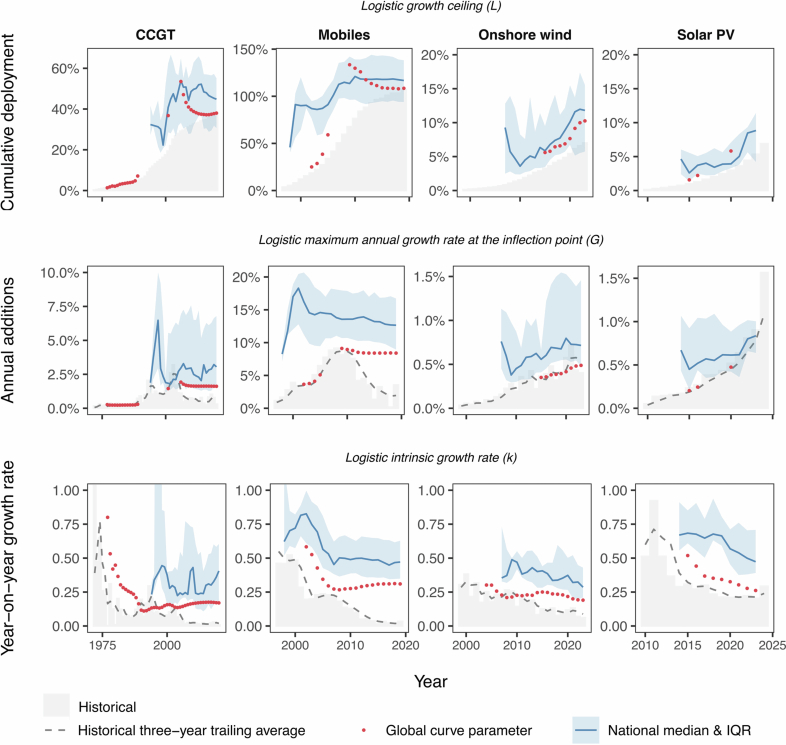
Evolution and stability of logistic function parameters for selected technologies. Gray bars show historical values and dashed lines their three-year trailing average. Red dots show parameters of logistic fits to global deployment. Solid blue lines indicate the median and shaded blue bands the IQR for samples of logistic parameters estimated from national trajectories. Top row: cumulative global deployment, and the logistic asymptote (*L*). Middle row: global annual additions, and the logistic growth rate at the inflection point (*G*). Bottom row: global year-on-year growth rate, and logistic growth parameter (*k*). Source data

First, because different phases of technology growth are shaped by distinct mechanisms, empirical observations must be segmented accordingly to yield meaningful extrapolations. We estimate take-off years to exclude the formative phase, which is uninformative for long-term trends. Post-take-off data are then used to estimate the growth constant *k*, which characterizes the accelerating growth phase. In countries that have passed the inflection point, we additionally estimate the peak growth rate *G*—a parameter shown to be robust to assumptions about the functional form of the S-curve^18^ and whose national distribution is stable over time for several technologies (Extended Data Fig. 5).

Second, many countries experience multiple growth pulses, which are poorly captured by standard S-curve functions with a single inflection point (Fig. 2 and Supplementary Note 2). To better reflect this pattern, we simulate national growth using both logistic and bilogistic functions^51^ (Methods).

Third, although countries adopt renewables at different times, the diffusion mechanisms are broadly similar across early and late adopters. PROLONG leverages mature growth trajectories in early adopters to infer probabilistic signals about global deployment dynamics.

Building on these insights, we model global renewables deployment as the aggregate outcome of national growth and international diffusion. For technologies still in early stages of global uptake, however, the relationship between national trajectories and eventual global patterns remains difficult to establish empirically. PROLONG addresses this challenge by combining Monte Carlo simulations with machine learning to systematically explore the space of possible relationships between national and global growth parameters.

**Fig. 3:**
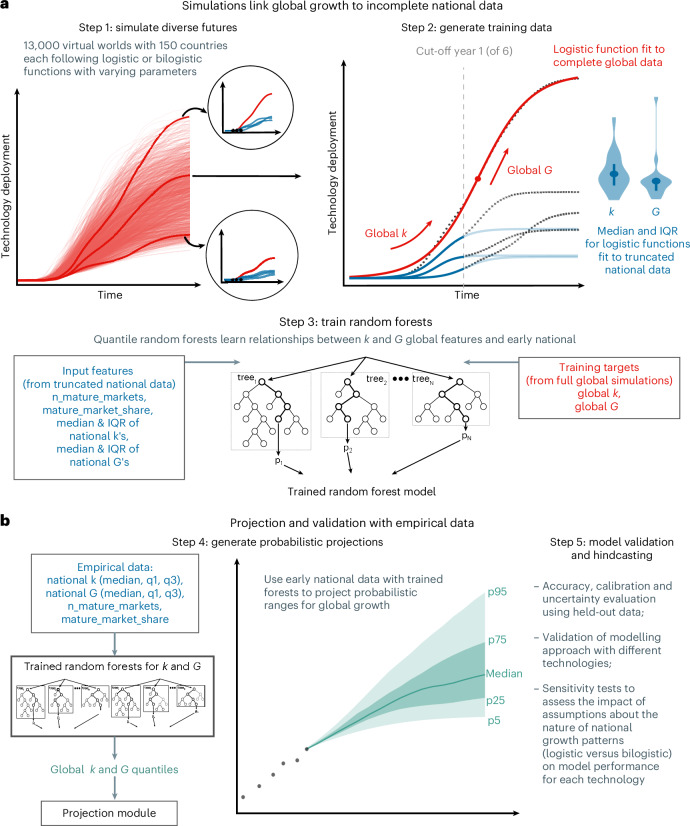
Five-step approach for PROLONG. **a**, Simulation and model training (Steps 1–3). **b**, Projection and validation with empirical data (Steps 4–5).

For each technology, we simulate 13,000 virtual worlds representing diffusion across 150 entities scaled to real-world country sizes. Each entity has a distinct take-off year and follows either a noise-perturbed logistic or bilogistic trajectory (Methods). These ensembles span both historically observed and theoretically plausible, but unobserved, growth patterns (Fig. 3, step 1, Supplementary Note 4 and Supplementary Fig. 2; Methods). We then train quantile random forests to learn how early national trends relate to global diffusion outcomes (steps 2 and 3; Methods). The result is a technology-specific probabilistic model capable of projecting global trajectories from incomplete national time series (step 4).

We validate each technology-specific model in three steps (Fig. 3, step 5). First, we conduct hindcasting using simulated test data by comparing model projections based on truncated national time series with their full global counterparts (Supplementary Note 4 and Supplementary Figs. 3 and 4). Second, we replicate the procedure with empirical data to assess how well the models reproduce historical global deployment. Third, to evaluate sensitivity to national growth assumptions, we compare model variants trained on ensembles with logistic, bilogistic or mixed functional forms and retain the best-performing variant for each technology (Supplementary Fig. 5; Methods).

**Fig. 4:**
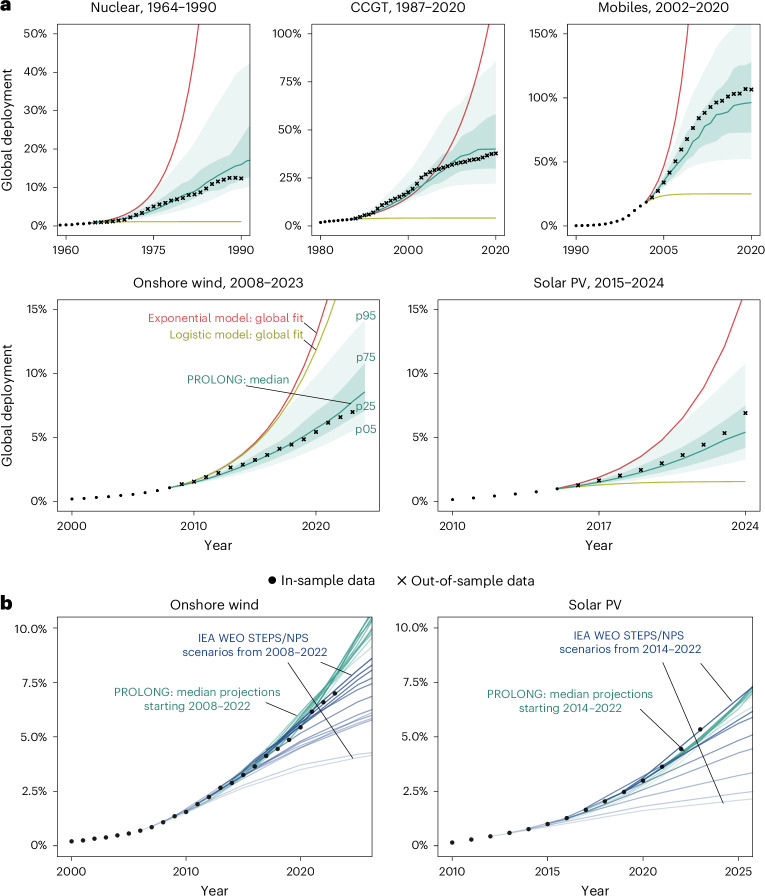
Comparison of projection models for historical and contemporary technologies. **a**, Hindcasting comparison for five technologies (as share of electricity for renewables and nuclear, gas-powered generation for CCGT and population for mobiles): dots show in-sample data; crosses show out-of-sample data. Solid lines indicate projections for exponential (red) and logistic (olive) models fitted to global data and the median projection from PROLONG (green). Shaded areas show PROLONG IQR (dark green) and 5th–95th percentile range (light green). **b**, Median PROLONG projections for global deployment from hindcasts initiated between 2008–2022 (onshore wind) and 2014–2022 (solar PV) compared with the IEA World Energy Outlook (WEO) STEPS and NPS projections from the same years^50^. Source data

**Extended Data Fig. 6:**
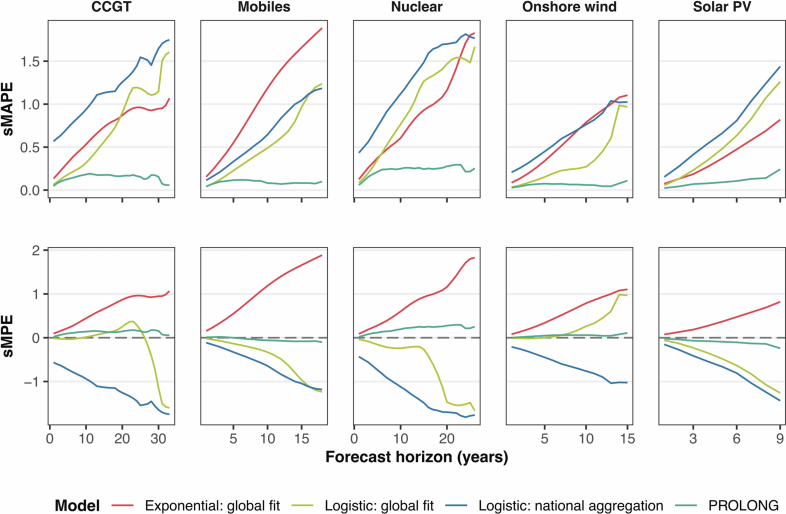
Comparative projection performance of PROLONG and other models across technologies and forecast horizons. Projection accuracy for PROLONG (green), aggregation of national logistic (blue), global exponential (red) and global logistic (olive) extrapolations, evaluated against empirical out-of-sample data. Top row shows the symmetric Mean Absolute Percentage Errors (sMAPE) and bottom row the symmetric Mean Percentage Errors (sMPE). PROLONG consistently demonstrates higher accuracy than other methods. Source data

We conduct this analysis for wind and solar power and then extend this validation to several reference technologies that have largely completed their global diffusion: combined-cycle gas turbines (CCGTs), nuclear power and mobile telephones (Extended Data Fig. 2). These technologies span opposite ends of the spectrum in terms of complexity and policy dependence: CCGTs and nuclear are complex, policy-driven systems^35^, while mobile telephones represent a relatively simple, market-driven technology^52^. For each case, we benchmark PROLONG against simpler extrapolation methods, including global logistic^11,24^ and exponential^9,49^ curve fits (Fig. 4), as well as aggregated national logistic projections (Extended Data Fig. 6 and Supplementary Figs. 6 and 7; Methods).

**Extended Data Fig. 7:**
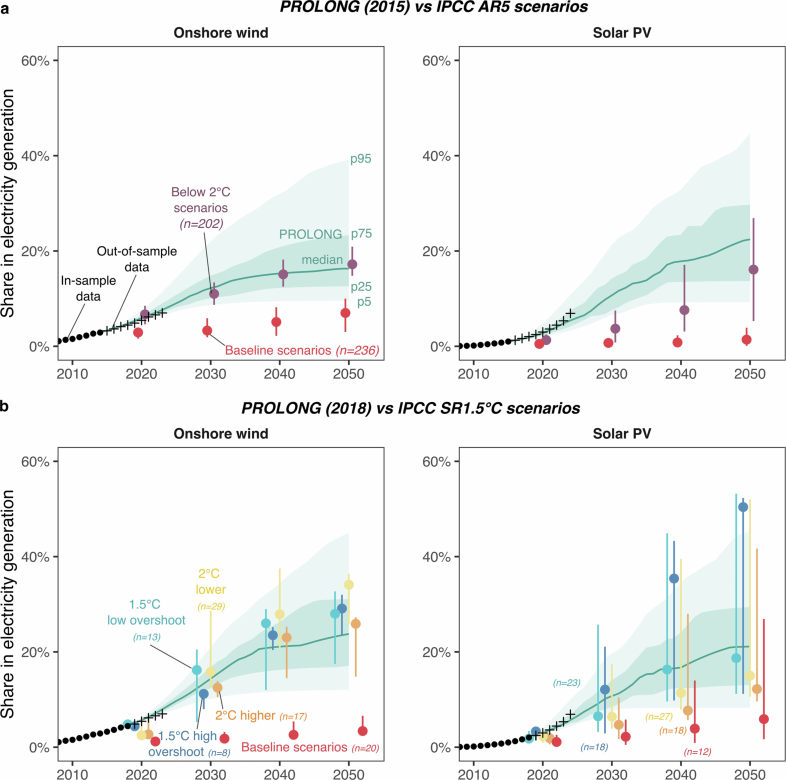
Comparing PROLONG projections to IPCC scenarios from AR5 and SR1.5°C reports. (a) Hindcasts from PROLONG using data through 2015 compared with baseline and below 2°C scenarios from the Intergovernmental Panel on Climate Change’s (IPCC) Fifth Assessment Report (AR5)^77^. (b) Hindcasts using data through 2018 compared with baseline and mitigation scenarios from the IPCC Special Report on 1.5°C (SR1.5°C)^78^. Black dots show in-sample historical data, crosses show out-of-sample historical data used for evaluation. Solid green lines show the median of PROLONG’s projections with shaded areas showing the interval between its 25-75th (dark green), and 5-95th (light) percentile projections. Coloured point-range lines depict deployment distributions with each ensemble’s median as a central dot and vertical lines capped at the 25th and 75th percentiles. PROLONG central projections more accurately capture out-of-sample developments compared to the baseline scenarios from both reports. Source data

Across five technologies and multiple forecast horizons, hindcasting consistently shows that PROLONG outperforms alternative projection methods (Extended Data Fig. 6 and Supplementary Figs. 6 and 7). For example, using data through 2015, PROLONG projected 2023 global electricity shares of 7.8% (IQR 7.1–8.8%) for onshore wind and 4.8% (3.6–6.3%) for solar—closely matching the actual 7% and 5.3%. By comparison, exponential fits overestimated deployment (13% wind, 12% solar), while logistic extrapolations were too conservative (5% wind, 1.5% solar). In addition to higher accuracy, PROLONG yields well-calibrated uncertainty intervals, as shown by our analysis of coverage rates, probabilistic skill scores and residual errors (Supplementary Fig. 8 and Supplementary Note 5). Hindcasts from multiple starting years further demonstrate that PROLONG more closely tracks observed outcomes than the International Energy Agency (IEA)’s ‘current policy’ and IPCC ‘baseline’ scenarios (Fig. 4 and Extended Data Fig. 7), probably reflecting its more realistic representation of policy-driven growth and cross-country diffusion.

## Probabilistic projections and scenarios of wind and solar growth

**Fig. 5:**
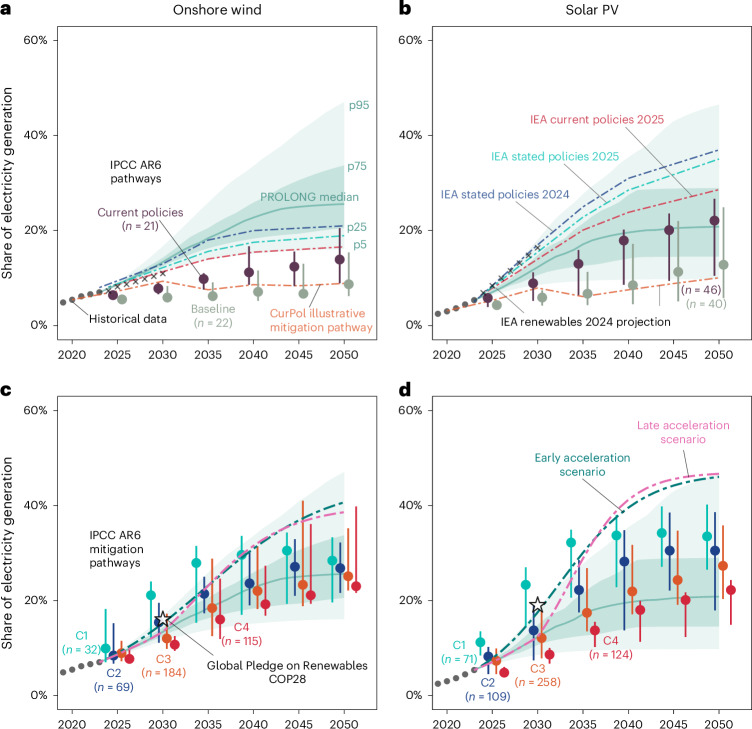
Probabilistic projections and mitigation-aligned scenarios for global onshore wind and solar PV deployment. **a**–**d**, Projected shares of global electricity from onshore wind (**a** and **c**) and solar PV (**b** and **d**) between 2023 and 2050. Black dots show historical data. Solid green lines show the median of PROLONG’s projections, with shaded areas showing the interval between its 25th–75th (dark green), and 5th–95th (light) percentile projections. Stars mark 2030 deployment levels aligned with the COP28 Global Renewables Pledge^5,76^. Coloured point-range lines depict deployment distributions for current policy, baseline scenarios (**a** and **b**) and climate mitigation pathways (**c** and **d**) from the IPCC AR6^12^ with each ensemble’s median as a central dot and vertical lines capped at the 25th and 75th percentiles. Dot–dash lines show the current policies (CurPol) illustrative mitigation pathway from the IPCC AR6, scenarios from the IEA World Energy Outlook^4,74^ (**a** and **b**) and early and late acceleration scenarios aligned with reaching deployment levels consistent with the IEA NZE scenario^4^ and IPCC AR6 1.5 °C no/limited overshoot^12^ pathway medians by 2040 (**c** and **d**). Source data

**Extended Data Fig. 8:**
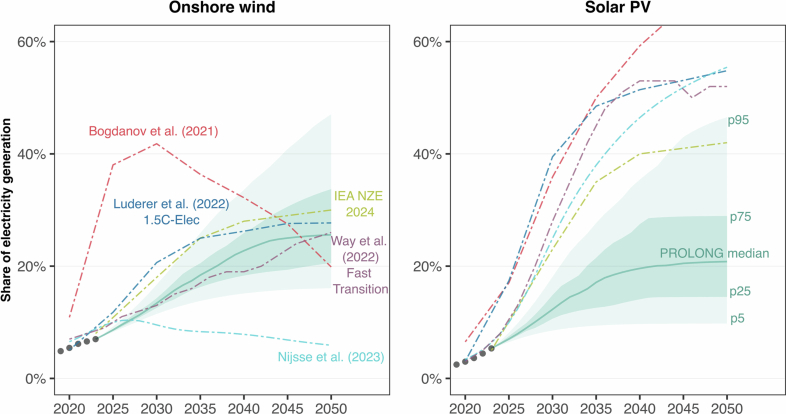
Comparison of PROLONG projections with selected literature scenarios for wind and solar growth in 2023-2050. Black dots show in-sample historical data. Solid green lines show the median of PROLONG’s projections with shaded areas showing the interval between its 25-75th (dark green), and 5-95th (light) percentile projections. Dot-dash lines show projections from refs. ^4,8,9,14,75^. Source data

We use PROLONG to generate probabilistic projections of solar and wind power deployment through 2050 (Fig. 5 and Table 2). Median estimates show onshore wind reaching 13.4% (IQR 12.6–14.3%) of global electricity generation by 2030, rising to 22.8% (18.3–26%) in 2040 and 25.6% (20.5–33.7%) by 2050. For solar PV, projected shares are 12.3% (10.6−15%) in 2030, 19.6% (14–27.5%) in 2040, and 20.8% (14.5−29%) by 2050. At the 95th percentile, onshore wind could reach 17%, 34% and 47% by 2030, 2040 and 2050, while solar could reach 18%, 35% and 47%. We compare these projections with the Global Renewables Pledge, baseline and current policy scenarios, climate mitigation pathways (Table 2 and Fig. 5) and other scenarios and forecasts from the literature (Extended Data Fig. 8).

**Table 2:** Comparing global onshore wind and solar PV shares in electricity generation in scenarios assessed by the IPCC AR6, the global pledge to triple renewables capacity adopted at the 28th Conference of Parties (COP28) and PROLONG probabilistic projections

Technology	Year	IPCC AR6 pathways			COP28 pledge	PROLONG projections				
		<1.5 °C no/limited overshoot; median (IQR)	Likely <2 °C; median (IQR)	Current policies; median (IQR)		p05	p25	Median	p75	p95
Onshore wind	2030	21%	12%	7.8%	16%	11.5%	12.6%	13.4%	14.3%	16.7%
		(12–24%)	(9.8–17.2%)	(6.6–9%)						
	2040	29.6%	22%	11.2%		15.1%	18.3%	22.8%	25.9%	33.6%
		(17–33.6%)	(18.2–31.4%)	(8.6–16.7%)						
	2050	28.4%	25.1%	13.9%		16.1%	20.5%	25.6%	33.7%	47.1%
		(19.6–33.3%)	(22.1–35.2%)	(8.9–20.5%)						
Solar PV	2030	23.3%	12.1%	8.9%	19%	8.5%	10.6%	12.3%	14.8%	18.1%
		(14.3–27%)	(7.8–17.9%)	(5.2–11.2%)						
	2040	33.7%	21.9%	17.9%		9.7%	14.1%	19.6%	27.5%	35.4%
		(22.8–38%)	(17.1–31.7%)	(8.6–20.2%)						
	2050	33.5%	27.3%	22.1%		9.8%	14.5%	20.8%	28.9%	46.5%
		(26.5–40.2%)	(20.3–35.8%)	(10.5–26.7%)						

The ranges for IPCC AR6 scenarios reflect the span of scenario outcomes across multiple models and assumptions, without systematic or probabilistic sampling of the full possibility space. The ranges for PROLONG represent the probability distribution of simulated trajectories (p05 stands for 5th-percentile and so on). These two types of range are therefore not directly comparable, but are shown here to illustrate the contrast between empirical and scenario-based approaches.

Our probabilistic projections show varying alignment with the most recent set of baseline and current policies scenarios across technologies and time frames (Fig. 5). For onshore wind, the IEA Stated Policies Scenario (STEPS)^4^ closely tracks our median projection, while most baseline and current policy scenarios from the IPCC’s Sixth Assessment Report (AR6)^12^ fall below our 25th percentile projection. For solar PV, baseline scenarios similarly fall below our 25th percentile projection, whereas current policies scenarios start below our median projection but gradually converge by 2040. The IEA’s STEPS exceed our 75th percentile for solar; however, the most recent (2025) update for the current policies scenario is lower than in 2024 and falls within PROLONG’s 50% interval (Fig. 5).

Projections consistent with the Global Pledge on Renewables and Energy Efficiency announced at COP28 in 2023^5^—which calls for tripling renewable energy capacity by 2030—lie near the 95th percentile of our projections. Compared with climate mitigation scenarios from the IPCC AR6, our IQR largely overlaps with 2 °C-compatible pathways for both technologies, as well as with 1.5 °C high overshoot pathways for onshore wind power. However, the more ambitious 1.5 °C no/limited overshoot pathways and the IEA Net Zero Emissions by 2050 (NZE) pathway^4^ exceed our 95th percentile projection during 2030–2035, particularly for solar. This gap narrows after 2035, as mitigation pathways reach saturation while our upper projection range continues to expand—by 2050, our 75th percentile encompasses even the most ambitious onshore wind pathways and approaches the median of stringent solar pathways.

**Extended Data Fig. 9:**
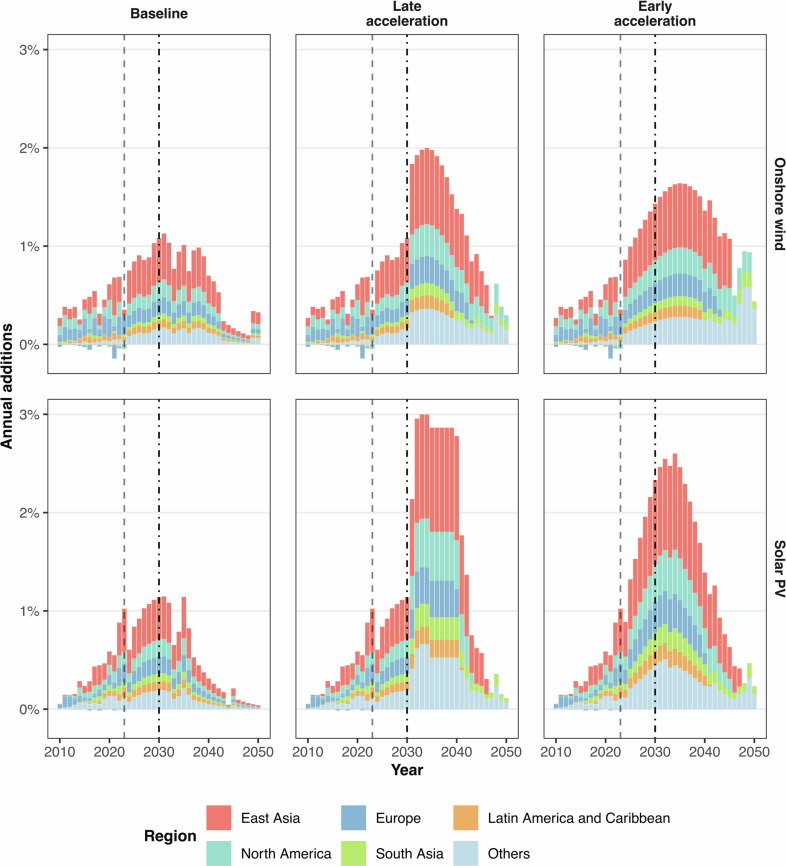
Regional growth of onshore wind and solar PV historically, under baseline and acceleration scenarios. Stacked bars show annual additions in the technology’s share in global electricity generation, segregated by region for each scenario: Baseline (aligned with PROLONG median), Early Acceleration (aligned with meeting the COP28 Global Pledge (refs. ^5,76^) by 2030 and median levels in 1.5°C no/limited overshoot pathways by 2040), and Late Acceleration (delayed action until 2030). Faint dashed lines mark 2023 (the start year for the scenarios), dot-dash lines mark 2030 (the onset of the second pulse in the Late Acceleration scenario). Source data

To illustrate the policy effort required for climate targets, we construct regionally differentiated scenarios for onshore wind and solar deployment. For each technology, we define three scenarios: a baseline aligned with PROLONG’s median projection, and two acceleration scenarios consistent with either the median of IPCC AR6 1.5 °C no/limited-overshoot pathways or the IEA NZE target for 2040 (Fig. 5). Early acceleration assumes immediate growth to meet both the 2030 Global Renewables Pledge and 2040 mitigation goals. Late acceleration follows the baseline until 2030, then sharply accelerates to reach the 2040 target (Table 3). We model early acceleration using logistic functions with different ceilings *L* and late acceleration using bilogistic functions with post-2030 growth pulses (Table 3). Growth is optimized across ten global regions under constraints on deployment ceilings, annual growth and acceleration rates, based on empirical data and energy system feasibility (Methods; Extended Data Fig. 9, Supplementary Figs. 9–12 and Supplementary Note 6).

**Table 3:** Growth of onshore wind and solar PV in the baseline, early acceleration and late acceleration scenarios

	Global growth assumptions		Global (bi-)logistic parameters	
	Before 2030	After 2030	Onshore wind	Solar PV
Baseline	In line with median of PROLONG projections		Ceiling 28% Δ*t* = 29 years	Ceiling 21% Δ*t* = 20 years
Early acceleration	In line with COP28 Global Pledge on Renewables for 2030	In line with IEA NZE or 1.5 °C by 2040	Ceiling 45% Δ*t* = 29 years	Ceiling 47% Δ*t* = 20 years
Late acceleration	Same as baseline	In line with IEA NZE or 1.5 °C by 2040	Ceiling 40% Δ*t*_2_ = 22 years	Ceiling 47% Δ*t*_2_ = 15 years

The IEA NZE scenario is from ref. ^4^, and the 1.5 °C-compatible (no/limited overshoot) scenarios from ref. ^12^; ‘ceiling’ refers to the asymptote *L* of the logistic or bilogistic function (estimated in case of baseline and assumed in case of acceleration scenarios) as share of total global electricity generation; Δ*t* is *ln*(81)/*k*, where *k* is the estimated/assumed logistic growth constant; Δ*t*_2_ is *ln*(81)/*k*_2_, where *k*_2_ is the assumed growth constant of the secondary pulse (starting in 2030) of the bilogistic growth function of the late acceleration scenario.

In the baseline scenario, about 73% of global onshore wind deployment (2023–2050) occurs in Europe, East Asia and North America, with annual additions accelerating to ~1.3 percentage points (pp) of electricity generation per year in Europe, East Asia and Latin America and the Caribbean (LAC), ~1 pp yr^−1^ in North America, and ~0.8 pp yr^−1^ elsewhere. Early acceleration requires annual growth of ~1.8 pp yr^−1^ in Europe and East Asia, ~1.6 pp yr^−1^ in North America, and up to ~2.7 pp yr^−1^ in other regions—similar to the European Union’s REPowerEU plan^53^ but requiring East Asia and North America to accelerate 1.4–1.6 times their previous peak rates, and other regions 2–14 times faster. The late acceleration scenario demands even steeper growth post-2030—~2.2 pp yr^−1^ in Europe, East Asia and LAC—requiring 1.4–8.7× increases over historical rates.

For solar power, the baseline scenario projects East Asia, Europe and North America contributing about 68% of global growth from 2023 to 2050, with annual additions reaching 1.4 pp yr^−1^ in Europe, 1.5 pp yr^−1^ in LAC, 1.2 pp yr^−1^ in East Asia and 1 pp yr^−1^ elsewhere. Early acceleration requires all major regions to exceed 2.7 pp yr^−1^—2–4 times their historical peaks surpassing China’s record in 2024. While ambitious, such rates are not unprecedented; for instance, India’s Central Electricity Authority targets ~2.4 pp yr^−1^ through 2030, up from its current 1 pp yr^−1^ (ref. ^54^). Under late acceleration, regional growth peaks at ~3 pp yr^−1^ post-2030. Both acceleration scenarios converge towards 45−50% solar shares by 2050 in most regions.

## Discussion and conclusions

Mainstream climate and energy scenarios rely on IAMs and ESOMs to project renewable energy growth. These models typically deploy renewables once they become cost-effective under assumed policy constraints such as carbon pricing or emissions caps. Many also anticipate future cost reductions through intertemporal optimization and account for system integration costs by balancing renewables with complementary technologies. However, optimization algorithms do not fully capture the political, institutional, and social dynamics that drive path dependence, inertia and other nonlinearities in large sociotechnical systems^15^. As a result, IAMs may project transitions that are unrealistically fast or slow^17–19^, prompting calls for alternative approaches^16^.

To address this gap, we introduce PROLONG, a probabilistic framework for projecting global technology growth from national deployment trajectories and international policy and technology diffusion patterns. By identifying regularities in historical adoption across diverse markets, institutions and social contexts, PROLONG provides a conceptual and empirical foundation for projecting future growth. It complements process-oriented models like IAMs and advances existing data-driven approaches^6,10,11,24,49^ by formalizing three insights about renewable energy diffusion.

First, we segment national growth trajectories into formative, accelerating and steady growth phases^18,33^, each governed by distinct mechanisms that provide different inputs for projections. Second, many countries experience multiple growth pulses—often triggered by policy shifts^33,43^—that standard S-curves fail to capture. We model these using bilogistic functions, departing from earlier interpretations of slow-downs as either temporary fluctuations^55,56^ or signs of imminent saturation^11,24^. Third, despite variation in adoption timing, growth mechanisms are broadly similar across countries, enabling us to use evidence from frontrunners to inform global projections.

We implement these insights in a machine learning framework trained on thousands of Monte Carlo simulations, creating a virtual laboratory of plausible futures and enabling robust uncertainty quantification. In doing so, PROLONG overcomes a key limitation of time-series extrapolation—its inability to explore multiple futures from a single historical trajectory—while also addressing the complementary weakness of scenario-based models, which cannot assign likelihoods to outcomes owing to their reliance on exogenous assumptions^57,58^.

These innovations allow PROLONG to generate probabilistic, medium-term projections of wind and solar power growth. In hindcasting, its median projections are generally higher and more accurate than past ‘current policy’ and ‘baseline’ scenarios from the IEA and the IPCC (Fig. 4 and Extended Data Fig. 7), reflecting its ability to capture empirically observed growth patterns often associated with policy change and cross-country diffusion—rather than assuming unchanged policies as in typical baseline models^59^. Looking ahead, PROLONG also projects higher median growth for wind and comparable projections for solar relative to current policy scenarios (Fig. 5).

PROLONG’s projections also provide a lens for assessing the policy effort implied by climate mitigation pathways. In our median estimates, renewables grow more slowly—but over a longer period—than in many 1.5 °C-compatible pathways, echoing earlier analyses of the extraordinary policy effort required to meet such targets^18,23,57^. Achieving this acceleration would require replicating exceptional cases such as the EU’s REPowerEU or China’s record solar growth in 2024—both of which face uncertain futures^60,61^. This underscores that such acceleration cannot rely on technology evolution alone, but requires extraordinary policy effort. Our approach complements literature quantifying policy effort in economic terms^62,63^ by also capturing non-economic barriers—social, political, institutional and infrastructural—that increasingly shape the pace of renewable energy transitions^43,61^.

PROLONG fills a methodological gap between short-term technology forecasts and long-term system-wide scenario modelling. At the same time, industry projections based on project-level data are typically more accurate over short horizons (<5 years) owing to the lag between empirical inputs and probabilistic outcomes in PROLONG. Conversely, IAMs and ESOMs remain better suited for estimating the scale of deployment needed to meet climate targets under varying assumptions about policy, technology and economics, and for systematically accounting for geophysical limits and evolving energy demand. PROLONG projects growth rates rather than saturation levels (Methods), which explains its stronger performance during accelerating and steady growth phases. However, being based on past trends, it cannot capture discontinuous shifts such as those triggered by geopolitical upheavals^61^.

Our analysis highlights several directions for future research. First, PROLONG could be extended to incorporate country-specific deployment ceilings based on physical resource potential and national characteristics, such as institutional capacity or electricity demand growth. Second, applying PROLONG at higher regional resolution could improve projections by better capturing growth in major economies such as China, the EU, India and the USA. Third, while PROLONG relies on the logistic function—which fits completed historical diffusion curves better than immediate alternatives (Supplementary Table 1) and enables transparent, parsimonious, reliable probabilistic projections—alternative S-curve forms could be explored. Finally, future work could examine the joint diffusion of complementary technologies (for example, storage and grids), integrate PROLONG’s probabilistic logic with IAMs and ESOMs, and account for sociopolitical dynamics such as competition for political attention, institutional capacity and social acceptance that shape multitechnology transitions.

This study helps to bridge qualitative insights and quantitative modelling of policy-driven energy transitions^16^, improving our ability to anticipate and inform climate mitigation strategies. By grounding projections in historical experience and probabilistic reasoning, PROLONG complements existing tools for exploring plausible renewable energy futures. As climate action becomes more urgent, empirically informed models like ours can help close the gap between aspirational goals and realistic trajectories—supporting more effective planning, policy design and public debate.

## Methods

### Technology focus and sample

Our analysis focuses on historical deployment of onshore wind and solar PV across 211 and 213 countries, respectively^1,64–66^. We restrict our analysis of wind to onshore deployment as the international diffusion of offshore wind remains too limited to yield a sufficient sample size (Supplementary Note 7) and plays a relatively modest role in long-term scenarios^18^.

To further validate our methods, we use several different technologies as reference cases^57^. These include mobile telephones^67^, CCGTs and nuclear power^68^, which were selected because they span diverse technological characteristics^52^ and diffusion timescales. All references cases are at relatively advanced stages of diffusion and provide suitable benchmarks.

For onshore wind and solar PV, deployment is measured as share in electricity generation and for other energy technologies as share in installed capacity. Mobile telephone deployment is measured as individual subscriptions per capita.

### Diagnosing national and global take-off

We define take-off as the end of a technology’s formative phase, marking the transition from sporadic deployment to sustained growth (Fig. 1 and Extended Data Fig. 1). To identify take-off years, we introduce a formative phase Bayesian change-point indicator (FOBI) implemented using the RBeast package in R (ref. ^69^). The detection algorithm uses a combination of piece-wise Bayesian regressions and model averaging to identify structural breaks in the time series where the trend (slope) of technology deployment shifts significantly upwards, indicating a transition from sporadic or experimental deployment to sustained growth. The algorithm detects multiple potential change points in the deployment time series along with their associated probabilities, then selects the earliest change point with sufficient statistical confidence as the take-off year, ensuring that the identified transition represents a robust shift towards sustained growth rather than temporary fluctuations. We include only deployment time series with at least five non-zero datapoints and identify both the year of take-off and the deployment level at take-off. We do not include pre-take-off data from the formative phase in diagnosing post-take-off growth or in calibrating our models because these data typically reflect failures, experimentation, rapid innovation and niche deployment while not capturing mechanisms shaping more mature phases of technology deployment.

### Quantifying cross-country diffusion

To measure the geographic spread of technologies, we track the cumulative number of countries reaching take-off over time. We then estimate the speed of cross-country diffusion by fitting a logistic curve to this count and calculating the diffusion duration Δ*T*_diffusion_—the time required for a technology to spread from 10% to 90% of the estimated maximum number of countries taking off (Extended Data Fig. 2 and Supplementary Note 1). This equals ln(81)/*k*, where *k* is the estimated logistic growth constant. These estimates enable comparison across technologies.

### Diagnosing inflection points and growth pulses

We identify inflection points—years of peak annual additions—using a curve-fitting approach from ref. ^18^. Logistic functions are fitted to deployment time series with Gauss–Newton (nls) and Levenberg–Marquardt (nlsLM) algorithms in R. The standard logistic form is $y(t)=\frac{L}{1+{{\rm{e}}}^{-k(t-{t}_{0})}}$, where *L* is the growth asymptote or ‘deployment ceiling’, *k* is the growth constant and *t*_0_ is the inflection point. We also calculate the curve maturity^18^, a diagnostic parameter defined as the ratio of the last observed value to *L*, to distinguish the accelerating growth phase (<50% maturity) from post-inflection or steady growth (≥50%) (Extended Data Figs. 2 and 3 and Supplementary Fig. 1).

Empirical observations often show multiple peaks or ‘growth pulses’ at the national level^70^. To detect these, we fitted logistic functions to progressively truncated time series. If the technology is firmly in the accelerating growth phase in a given cut-off year, no inflection point is detected (maturity is always <50%). If maturity rises past 50% and then later declines, the inflection point shifts forward, indicating a secondary pulse. This procedure formalizes the concept of a steady growth phase with policy-driven pulses (Fig. 2).

### Estimating the robustness of growth parameters

To assess the stability of logistic parameters, we fitted curves to progressively truncated series at national and global levels and then compare distributions of *L*, *k* and the derived peak annual growth rate $G=\frac{kL}{4}$ (ref. ^18^) across time. Estimates of *L* are unstable and highly sensitive to truncation, whereas *G* is relatively robust and can be reliably inferred from early national data (Extended Data Fig. 5).

### Constructing probabilistic models for projecting global growth

We developed PROLONG, a computational approach for probabilistic technology diffusion projections that links national adoption patterns to global diffusion (Fig. 3 and Supplementary Note 4). At the centre of our model is a machine learning algorithm that is trained to probabilistically predict global growth based on historical observations of early national growth.

To generate training data, we simulate 13,000 diffusion trajectories per technology in a ‘virtual world’ of 150 entities scaled to real-world country sizes. Each entity adopts the technology at a different time, following either a logistic^71^ or bilogistic^51^ function with correlated noise added to reflect real-world variability. In bilogistic cases, growth is split between two pulses, capturing policy-driven slow-downs and rebounds. Parameter values for take-off year, *k* and *L* are drawn from broad distributions to cover both observed and plausible-but-unseen dynamics (Supplementary Fig. 2 and Supplementary Tables 3–7).

For each simulation, we truncate national time series at different points, fit logistic curves and calculate distributions of *k* and *G* across mature countries (≥50% maturity), as well as two contextual variables: the number of mature countries and their cumulative global market share. These data are used to train quantile random forests^72^ (via ranger in R), which learn the conditional relationships between early national signals and eventual global outcomes.

To generate probabilistic global trajectories with real-world data, we sample quantiles of national parameters and use the trained random forests to probabilistically predict global *k* and *G*. We then sample parameter combinations at different quantiles (5th, 25th, 50th, 75th and 95th), generate trajectories using *t*_0_ = ((1/*k*) × *log*((*L*/*y*_*i*_) − 1)) + *t*_*i*_ (where *y*_*i*_ is current deployment and *t*_*i*_ is current year) and derive probabilistic projection intervals. Note that, in PROLONG, the models are trained to predict *k* and *G* from early signals rather than to directly infer *L*—we compute only an indicative ceiling *L* = 4*G*/*k*, which should not be interpreted as a firm estimate. Separate models are trained on logistic-only, bilogistic-only and mixed simulations, with technology-specific selection based on hindcasting performance.

### Model variant selection and validation through hindcasting

We validate PROLONG in three stages. First, we perform hindcasting on simulated test data, where we generate global projections using truncated national series from simulated ensembles not previously seen by the model and compare them with the known ‘out-of-sample’ simulated trajectories (Supplementary Note 4 and Supplementary Figs. 3 and 4).

Second, we replicate this hindcasting with real-world data for onshore wind and solar, testing how well the models predict ‘out-of-sample’ global trajectories when given only partial data. We measure accuracy using the symmetric mean absolute percentage error (sMAPE), which captures average forecast deviation, and the symmetric mean percentage error (sMPE), which indicates systematic bias^73^. Across horizons of 1–30 years, PROLONG maintains sMAPE values typically below 0.5 and sMPE values consistently close to zero, showing both accuracy and low bias. By contrast, exponential and logistic global fits often exceed 1.0 sMAPE and show large positive or negative biases that worsen with horizon.

Third, we assess sensitivity to assumptions about national growth patterns by training model variants on ensembles where all countries follow (a) logistic, (b) bilogistic or (c) mixed growth trajectories. We retain the best-performing variant for each technology (Supplementary Fig. 5).

We repeat validation for CCGTs, nuclear power and mobile telephones and benchmark PROLONG against simpler methods: global logistic and exponential fits, and aggregated national logistic extrapolations. Across all three technologies, PROLONG yields lower forecast errors and reduced bias compared with global curve fits and national aggregation methods, particularly in early-to-mid diffusion phases where simple extrapolation fails to anticipate changing dynamics (Fig. 4, Extended Data Fig. 6 and Supplementary Figs. 6 and 7).

### Uncertainty quantification

In addition to accuracy, we systematically evaluate the quality of PROLONG’s probabilistic forecasts. Rather than relying only on point estimates, we assess how well the models generate calibrated probability distributions that reflect the full range of plausible outcomes. To do this, we conduct hindcasting experiments in which we truncate historical deployment data, generate probabilistic forecasts and then compare them to withheld observations. We use a suite of complementary metrics that capture different aspects of uncertainty, including interval widths, coverage rates, relative uncertainty, interval scores and the continuous ranked probability score, alongside distributional diagnostics such as tail heaviness. These metrics allow us to test whether forecast intervals expand credibly with horizon, whether observed outcomes fall within predicted ranges at appropriate frequencies, and whether the underlying probability distributions are both informative and well calibrated. The results show that PROLONG produces uncertainty ranges that grow steadily but remain well behaved, achieves near-nominal coverage rates, maintains low forecast errors and avoids unrealistic fat-tailed behaviour. A full description of the metrics, their rationale and detailed results are provided in Supplementary Note 5.

### Comparing with IPCC scenarios and other projections/forecasts

We benchmark PROLONG’s probabilistic projections against widely used scenarios and forecasts. For the scenarios assessed by the IPCC, we calculate onshore wind and solar shares in global electricity from the AR6 scenario database^12^, including baseline (P1a), current policies (P1b) and all vetted mitigation pathways. Mitigation scenarios are grouped by temperature outcome: below 1.5 °C with no/limited overshoot (C1), below 1.5 °C with high overshoot (C2), probably below 2 °C (C3) and below 2 °C with >50% probability (C4). We restrict analysis to scenarios with complete data at 5-year intervals between 2025 and 2050 (Fig. 5). Our comparisons also include the ‘current policy’ illustrative mitigation pathway from ref. ^12^ and the IEA’s STEPS from the 2024 and 2025 editions of the World Energy Outlook^4,74^, as well as short- to medium-term market projections from the IEA Renewables 2024 report^1^ that reflect announced projects and market intelligence rather than normative scenarios. We also benchmark PROLONG against ambitious pathways including the Fast Transition scenario from ref. ^9^, the 1.5C-Elec scenario from ref. ^14^, the IEA’s NZE scenario^4^ and scenarios from refs. ^8,75^. To assess projections aligned with the Global Pledge on Renewables and Energy Efficiency announced at COP28 in 2023, which targets tripling renewable energy capacity by 2030, we use scenario data from ref. ^76^ to estimate the implied global electricity generation shares for solar and wind power. For scenarios reporting only total wind generation, we treat projections as aggregates of onshore and offshore wind power.

In addition to the above, we also compare PROLONG’s median hindcasts with contemporaneous IEA STEPS or New Policies Scenario (NPS) projections and with baseline and mitigation pathways in the IPCC AR5^77^ and SR1.5^78^, providing a historical test of comparative projection skill (Fig. 4 and Extended Data Fig. 7).

Unlike deterministic scenarios, PROLONG provides calibrated probability distributions. This makes it possible to evaluate not just central trajectories but also the plausibility of high- and low-growth outcomes, offering a complementary perspective on the credibility of established policy and industry pathways.

### Developing regionally differentiated acceleration scenarios

To explore policy effort consistent with 1.5 °C pathways, we construct a baseline (median PROLONG projection) and two acceleration scenarios: early acceleration with a higher deployment ceiling (*L*) but unchanged *k*, representing immediate policy action, and late acceleration with a second growth pulse after 2030 with new parameters (*k*_2_, *L*_2_), representing delayed but intensified policy action.

For wind, early acceleration assumes *L* = 45%, while late acceleration assumes *L* = 40%, *k*_2_ = 0.2 from 2030. For solar, early acceleration sets *L* = 47%, and late acceleration sets *L* = 47%, *k*_2_ = 0.3 from 2030 (Table 2).

To translate these global trajectories into regional pathways, we distribute the required growth across ten world regions using a constrained optimization approach. Our algorithm allocates annual growth proportionally to available deployment headroom (difference between current deployment and regional ceiling). The optimization incorporates three empirically grounded constraints: maximum regional deployment ceilings (ranging from 30% to 55% depending on regional conditions and based on peak deployment levels in IPCC AR6 scenarios^12^; Supplementary Table 7), maximum annual growth rate (3-pp increase in market share per year) and maximum year-on-year acceleration in growth rates (1 pp). These constraints reflect practical limitations observed in historical technology diffusion patterns for large systems.

The algorithm iteratively redistributes growth where ceilings are reached (see Supplementary Note 6 for details). This yields regionally differentiated deployment pathways, enabling us to assess the trade-offs between early sustained versus delayed intensive policies under empirically grounded diffusion limits (Supplementary Figs. 9–12).

## Supplementary information

Supplementary Information Supplementary Figs. 1–14, Tables 1–9 and Notes 1–7.

## Source data

Source Data Fig. 1 Statistical source data.

Source Data Fig. 2 Statistical source data.

Source Data Fig. 4 Statistical source data.

Source Data Fig. 5 Statistical source data.

Source Data Extended Data Fig. 1 Statistical source data.

Source Data Extended Data Fig. 2 Statistical source data.

Source Data Extended Data Fig. 3 Statistical source data.

Source Data Extended Data Fig. 4 Statistical source data.

Source Data Extended Data Fig. 5 Statistical source data.

Source Data Extended Data Fig. 6 Statistical source data.

Source Data Extended Data Fig. 7 Statistical source data.

Source Data Extended Data Fig. 8 Statistical source data.

Source Data Extended Data Fig. 9 Statistical source data.

Source Data Extended Data Table 1 Statistical source data.

## Figures and Tables

**Extended Data Table 1:**
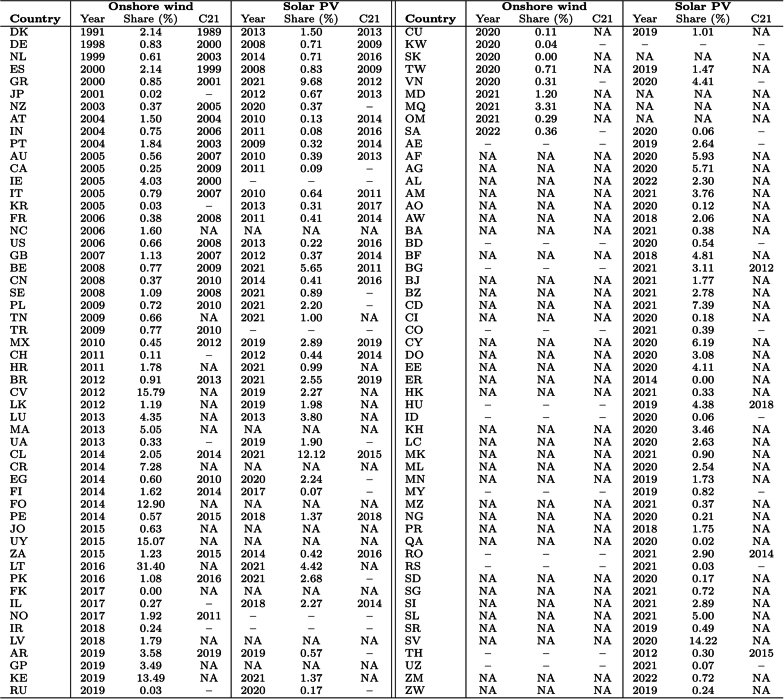
Solar PV and onshore wind takeoff years and deployment levels by country

Deployment levels are shown as shares in electricity generation at the takeoff year diagnosed by FOBI (Method). C21 refers to takeoff years reported by ref. ^18^, who assumed takeoff at 1% of electricity share. Country names reflect ISO 3166-1 alpha-2 codes.

Source data

## Data Availability

For the empirical analysis of onshore wind and solar PV growth, we use global and national electricity generation time series data from the IEA^1,64^, the International Renewable Energy Agency (IRENA)^65^ and EMBER^66^. For the analysis of their growth in scenarios, we use data from refs. ^1,4,8,9,12,14,50,75,77,78^. For the targets under Global Pledge on Renewables and Energy Effciency and the EU’s RePowerEU plan we use data from refs. ^76^ and refs. ^23,53^, respectively. For the analysis of CCGTs, we use licensed data from WEPP-PLATTS^68^. For the analysis of mobile telephones, we use mobile phone subscription data from the International Telecommunications Union (ITU) DataHub^67^ and population data from the United Nations Department of Economic and Social Affairs (UN-DESA)^79^. The data used for reproducing the main results of this Article are available via Zenodo at 10.5281/zenodo.18336350 (ref. ^80^). Source data are provided with this paper.

## Code availability

The code supporting the findings of this study is available via Zenodo at 10.5281/zenodo.18336350 (ref. ^80^).
