# Supplementary material for: Probabilistic projections of global wind and solar power growth based on historical national experience
Source: Nat Energy. 2026 Apr 14;11(5):743–55. doi: 10.1038/s41560-026-02021-w (PMC13215882; doi:10.1038/s41560-026-02021-w)
Supplement: Supplementary file 1 — Supplementary Figs. 1–14, Tables 1–9 and Notes 1–7. [file 41560_2026_2021_MOESM1_ESM.pdf]

nature energy

Supplementary information https://doi.org/10.1038/s41560-026-02021-w

Probabilistic projections of global wind and solar power growth based on historical national experience

In the format provided by the authors and unedited

Contents

Supplementary Figures 2

Supplementary Tables 17

Supplementary Notes 21

Supplementary Note 1: Technology growth and diffusion mechanisms and phases 21

Supplementary Note 2: Mathematical models for technology growth 24

Supplementary Note 3: Projecting global technology growth using national-level data 28

Supplementary Note 4: PROLONG (PRobabilistic mOdeL Of techNology Growth) 31

Step 0: Defining rules for the virtual worlds 32

Step 1: Exploring diverse technology futures using Monte Carlo simulations 34

Step 2: Generating training data from the ensemble of simulated trajectories 36

Step 3: Using machine learning to identify the relationships between truncated national and final global parameters 37

Step 4: Generating probabilistic projections from empirical data 37

Step 5: Model validation and hindcasting 39

Supplementary Note 5: Treatment and measurement of uncertainty in PROLONG 42

Types of uncertainty in technology modeling and how it is addressed in PROLONG . 43

Uncertainty quantification for solar PV and onshore wind 44

Supplementary Note 6: Acceleration scenarios for onshore wind and solar PV 46

Supplementary Note 7: The case of offshore wind power 49

Supplementary Figures

Supplementary Figure 1 Historical technology deployment curves.
Solid lines show global deployment measured in absolute units (green) and as percentage market share (blue); stars indicate takeoff as measured using the respective data.

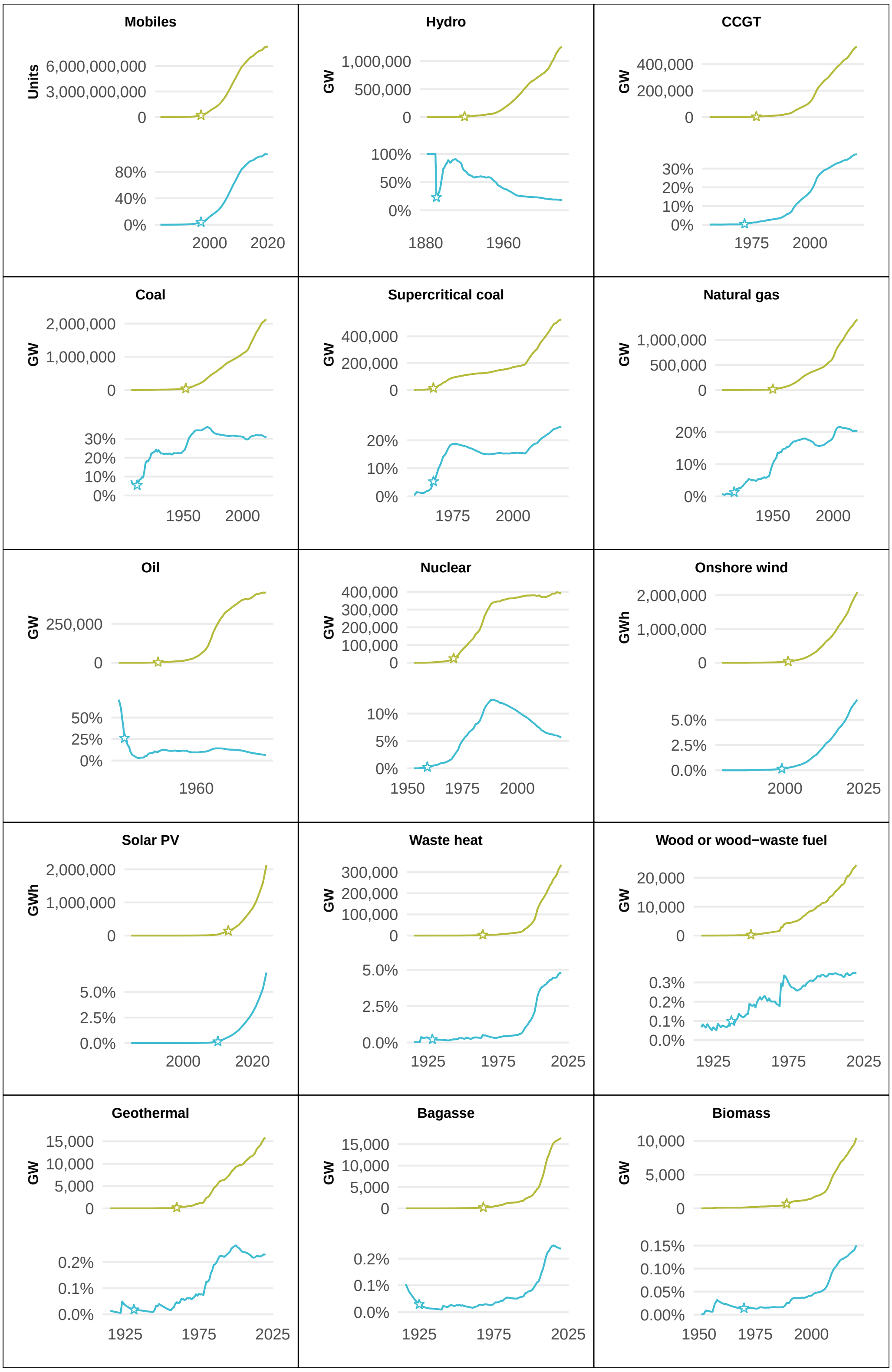

Supplementary Figure 2 Parameter configurations used for Monte Carlo simulations. Grid points (blue circles) and face-centered points (red-triangles) indicate the means of gamma distributions for growth rate (k) and saturation level (L) used in the hybrid coverage strategy for Monte Carlo simulations. The 3x3 grid (points 1-9) ensures systematic parameter space coverage, while face-centered configurations (points 10-13) improve coverage density. Actual simulation values are drawn from gamma distribution around these means. This hybrid sampling strategy balances broad coverage of plausible growth trajectories with computational efficiency.

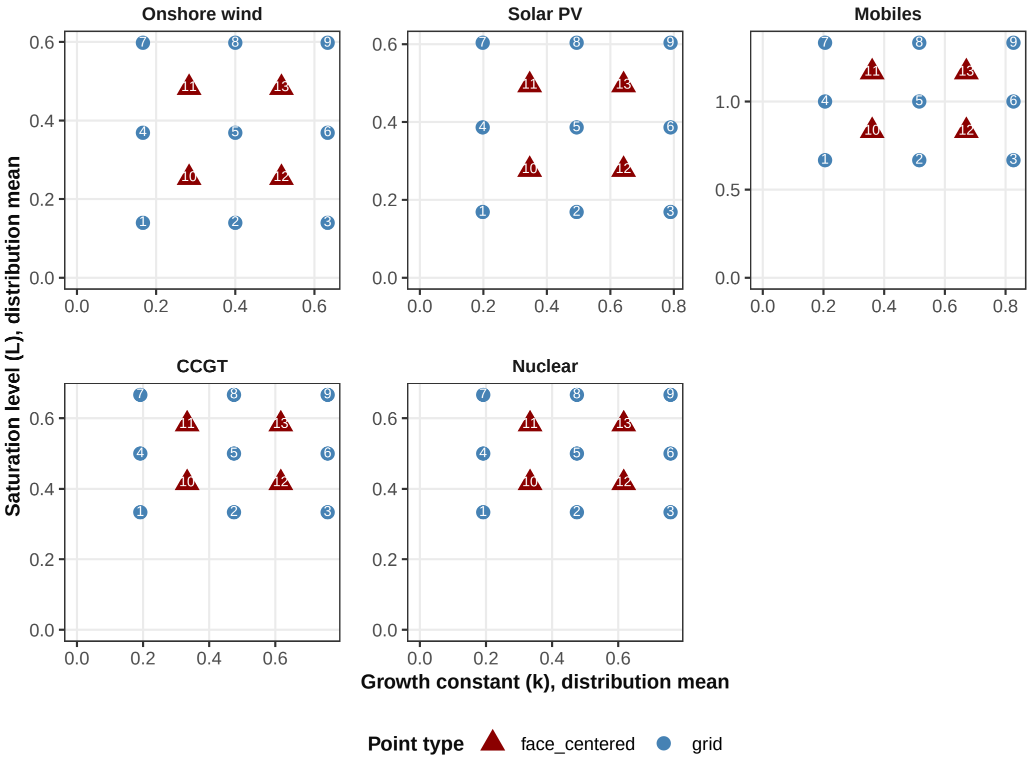

Supplementary Figure 3 Performance indicators for PROLONG models validated using simulated test data by forecast horizon.
Indicators measuring the projection performance of PROLONG models trained on simulations for onshore wind (cyan), solar PV (orange), mobiles (olive), combined cycle gas turbines (CCGTs, yellow) and nuclear power (purple). Panels show the mean widths of the 90% projection intervals, (b) mean relative uncertainty, (c) mean interval score for the 90% projection intervals, (d) mean continuous ranked probability scores (CRPS), (e) symmentric mean absolute percentage errors (sMAPE), and (f) for symmetric mean percentage error (sMPE) over different forecast horizons.

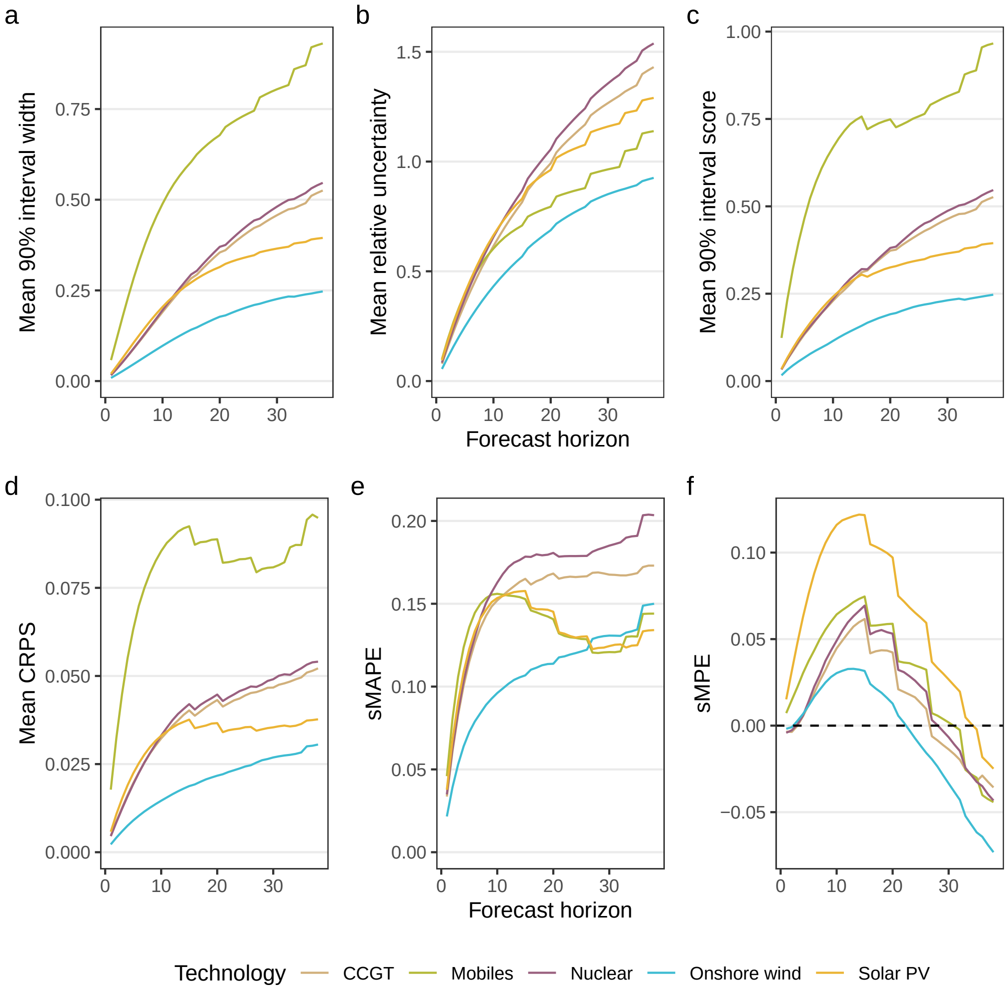

Supplementary Figure 4 Performance indicators for PROLONG models validated using simulated test data by year of truncation.
Indicators measuring the projection performance of PROLONG models trained on simulations for onshore wind (cyan), solar PV (orange), mobiles (olive), combined cycle gas turbines (CCGTs, yellow) and nuclear power (purple). Panels show the mean widths of the 90% projection intervals, (b) mean relative uncertainty, (c) mean interval score for the 90% projection intervals, (d) mean continuous ranked probability scores (CRPS), (e) symmentric mean absolute percentage errors (sMAPE), and (f) for symmetric mean percentage error (sMPE) for different truncated data.

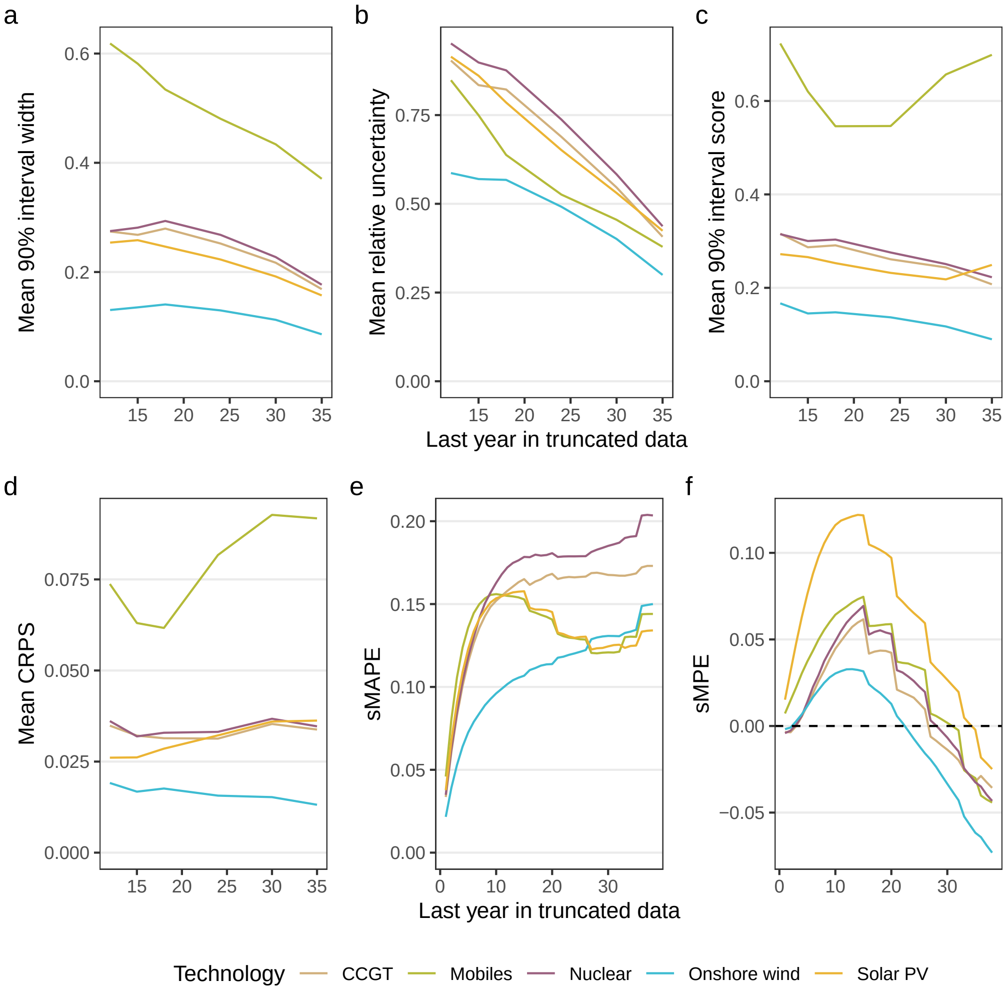

Supplementary Figure 5 Performance indicators for different variants of PROLONG models validated against empirical out-of-sample data.
Indicators measuring the projection performance of PROLONG models trained on simulations assuming individuals countries follow only bilogistic functions (yellow), only logistic functions (olive), or are equally likely to follow either function ('Mixed', green). The symmetric Mean Absolute Percentage Error (a) and symmetric Mean Percentage Error (b) for the median global projections from each model variant at different truncation years. The mean Continuous Ranked Probability score (c) for each model variant for different forecast horizons.

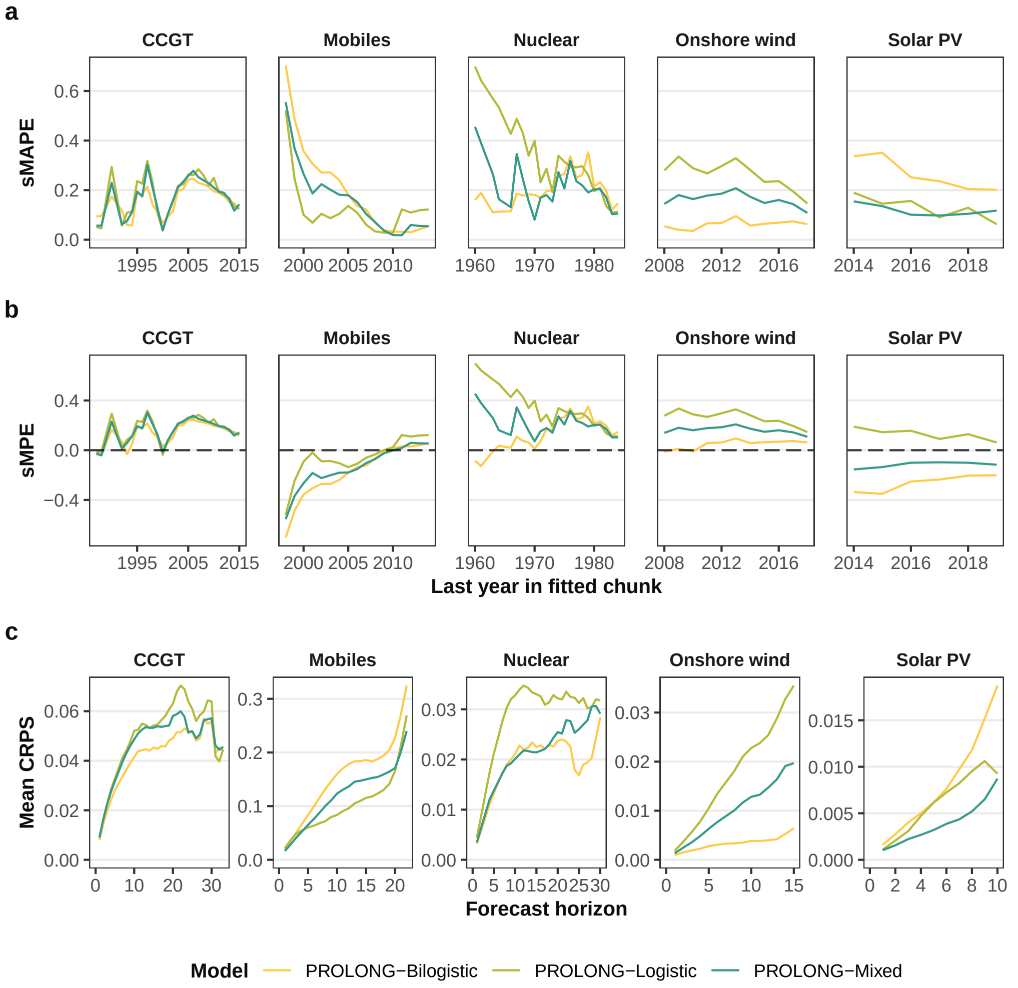

Supplementary Figure 6 Comparing performance indicators for PROLONG and other models validated against empirical out-of-sample data by truncation year.
Indicators measuring the projection performance of a PROLONG model (green), an aggregation of extrapolations for logistic curves fit to national data (blue), and extrapolations for exponential (red) and logistic (olive) curves fit to global time series data for different truncation years. Top row shows the symmetric Mean Absolute Percentage Errors (sMAPE) and bottom row the symmetric Mean Percentage Error (sMPE).

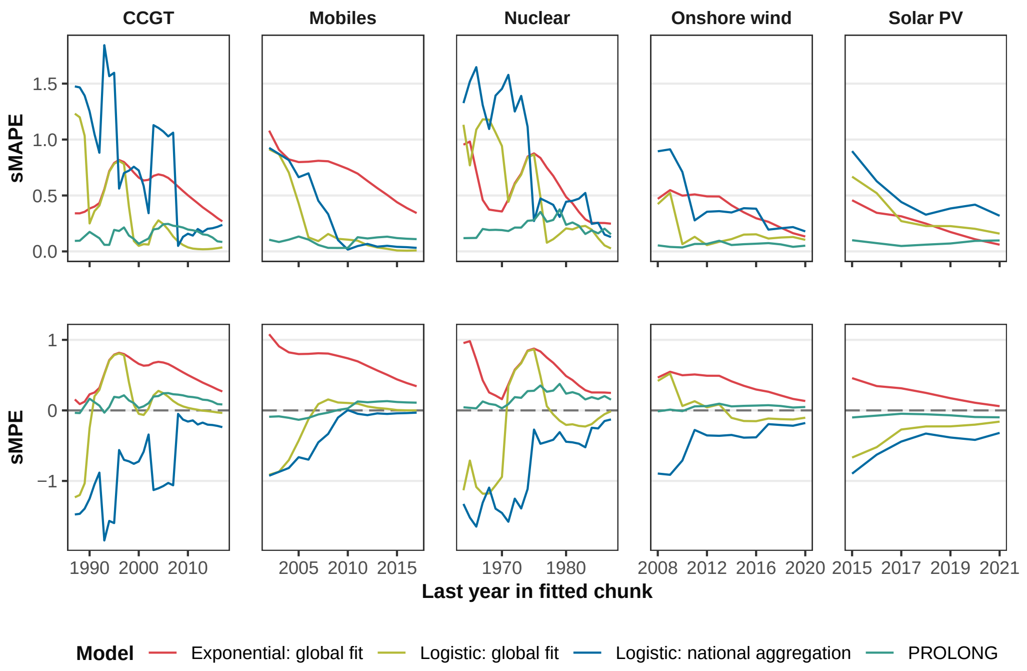

Supplementary Figure 7 Hindcasting comparison between median projections from PROLONG models and a bottom-up approach aggregating logistic extrapolations for individual countries. Green lines show median projections for PROLONG from different hindcast years between, and blue lines show the corresponding projections from the bottom-up aggregation approach. Projections shown start from the earliest year where both approaches can be used.

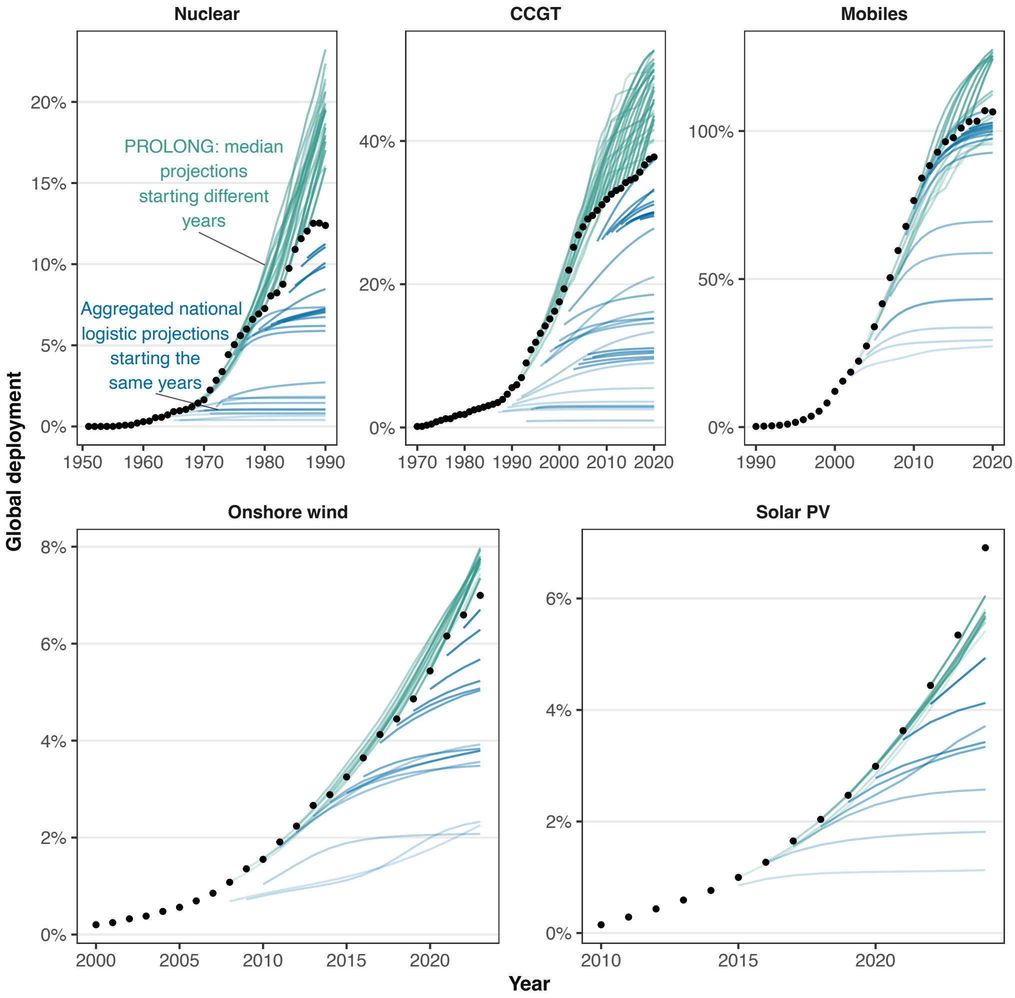

Supplementary Figure 8 Uncertainty quantification for PROLONG projections for onshore wind and solar PV validated against empirical out-of-sample data.
Indicators measuring different elements of uncertainty in PROLONG projections for onshore wind (cyan) and solar PV (orange) over different forecast horizons. Panels show (a) the mean widths of the 90% projection intervals, (b) mean coverage of the 90% projection intervals, (c) mean relative uncertainty, (d) mean interval scores for the 90% projection intervals, (e) mean continuous ranked probability scores (CRPS), and (f) mean tail heaviness. Note: We can only validate hindcasts empirically for horizons up to 10 years for solar PV and 15 years for onshore wind.

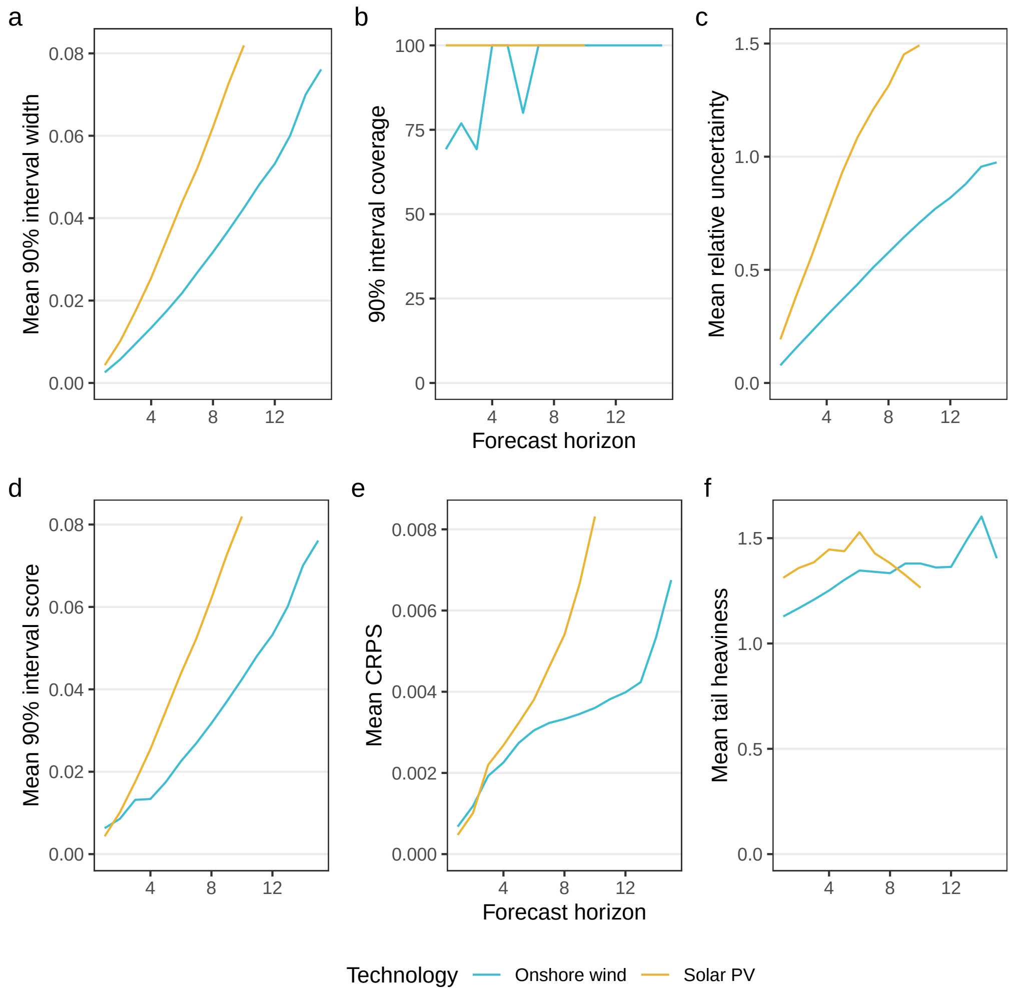

Supplementary Figure 9 Region-wise onshore wind deployment under the Baseline, Early and Late acceleration scenarios.
Dots show historical data; solid lines show deployment in the Basline (red), Early acceleration (green) and Late acceleration scenarios (blue).

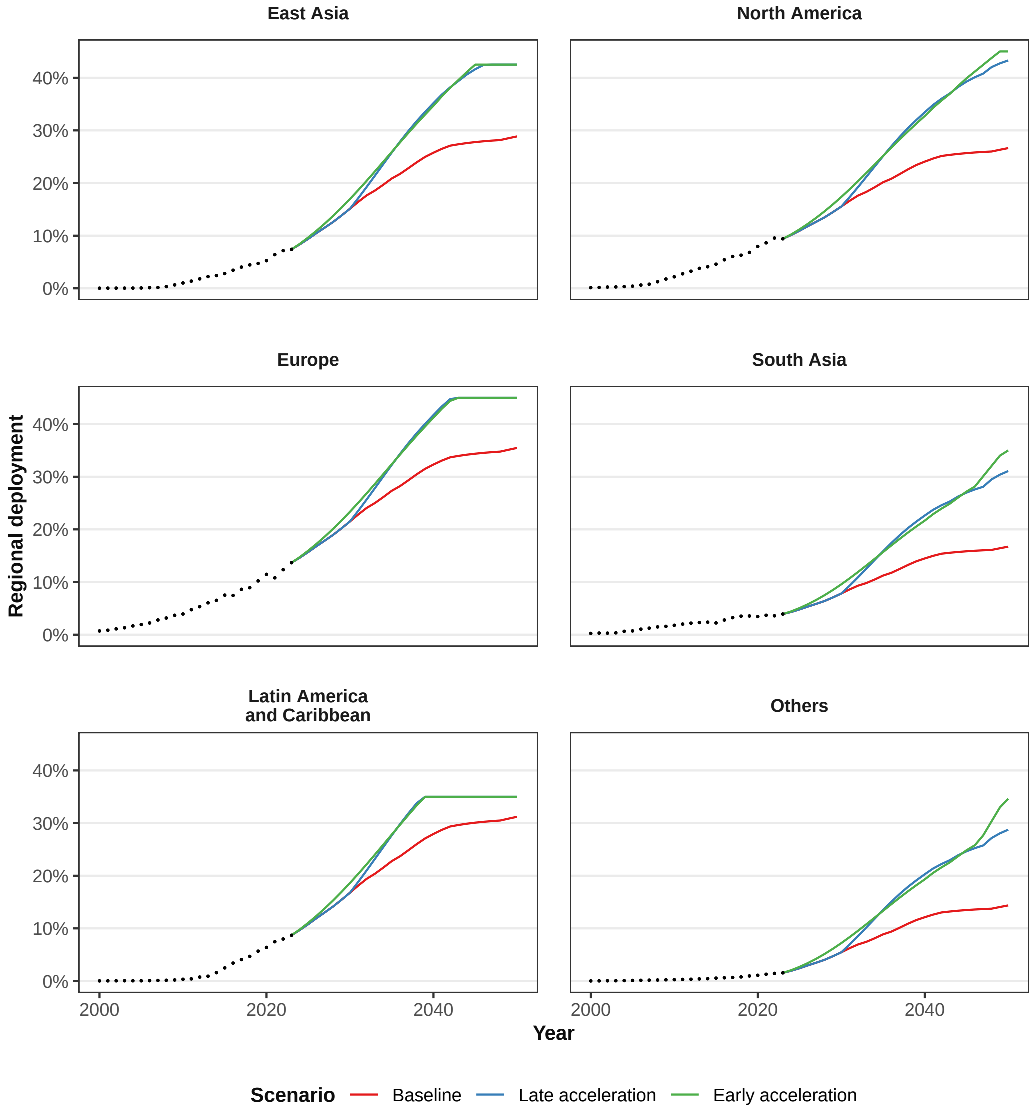

Supplementary Figure 10 Region-wise annual onshore wind additions under the Baseline, Early and Late acceleration scenarios.
Gray bars show historical data; coloured bars show deployment in the Basline (red), Early acceleration (green) and Late acceleration scenarios (blue).

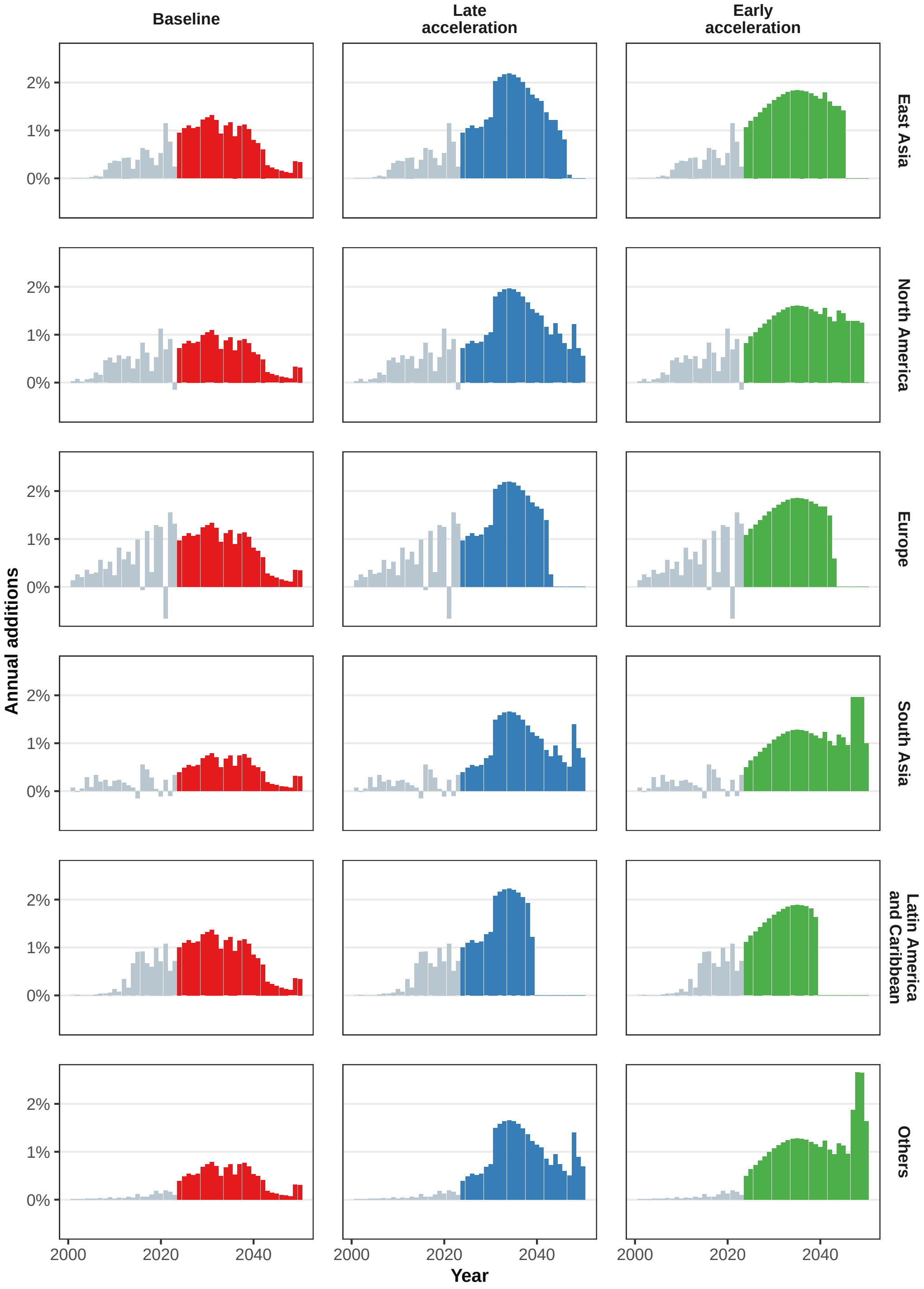

Supplementary Figure 11 Region-wise solar PV deployment under the Baseline, Early and Late acceleration scenarios.
Dots show historical data; solid lines show deployment in the Basline (red), Early acceleration (green) and Late acceleration scenarios (blue).

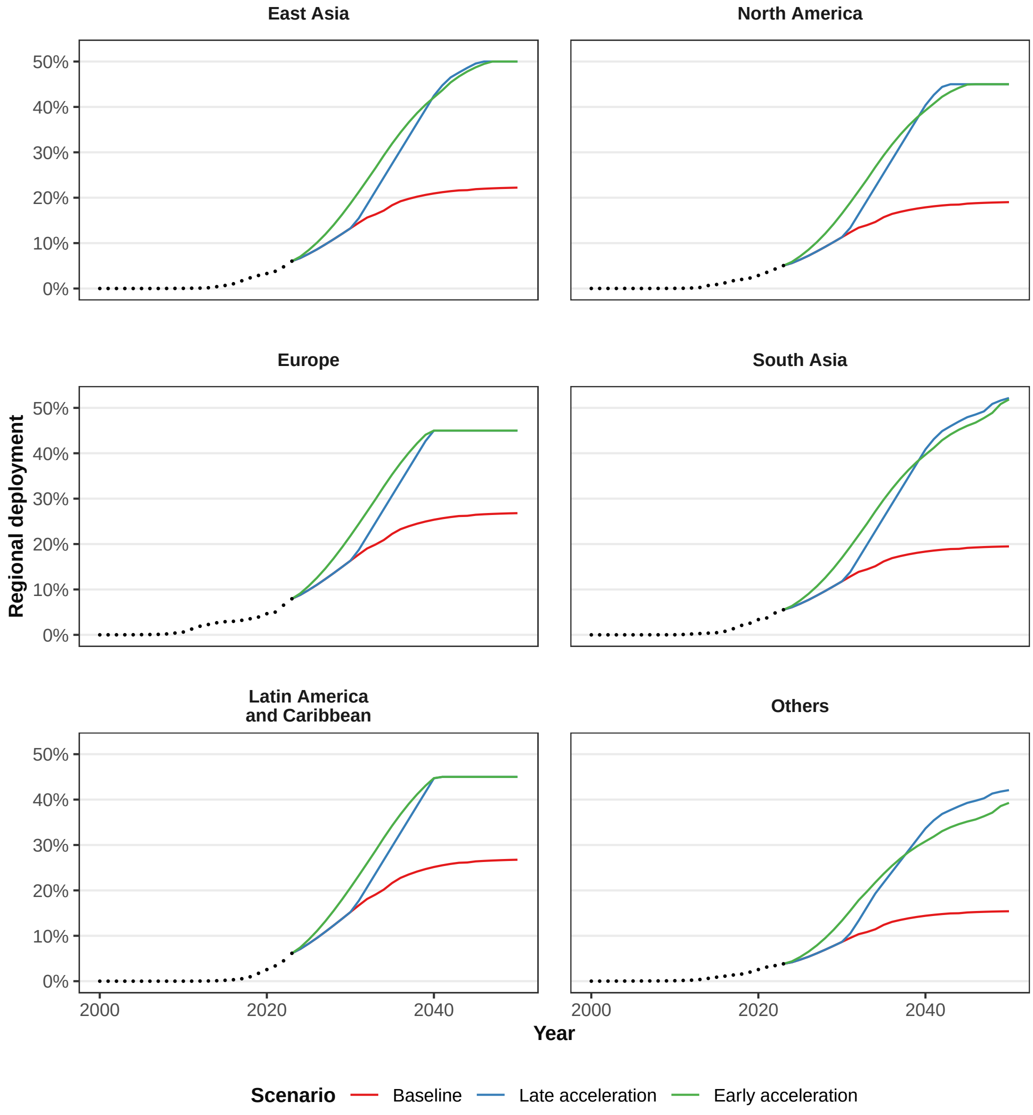

Supplementary Figure 12 Region-wise annual solar PV additions under the Baseline, Early and Late acceleration scenarios.
Gray bars show historical data; coloured bars show deployment in the Basline (red), Early acceleration (green) and Late acceleration scenarios (blue).

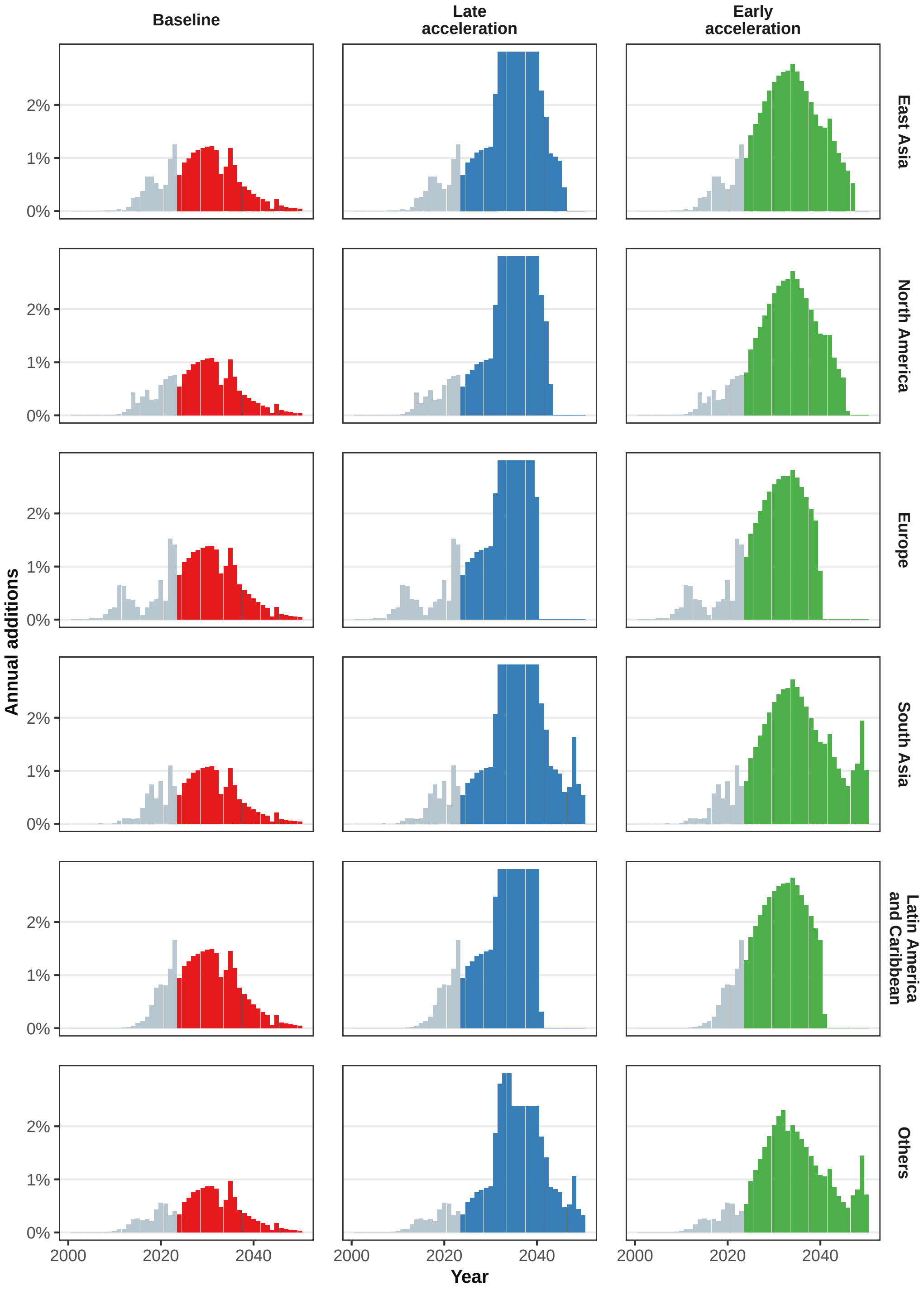

Supplementary Figure 13 Offshore wind deployment
Solid lines show historical deployment; stars indicate technology takeoff.

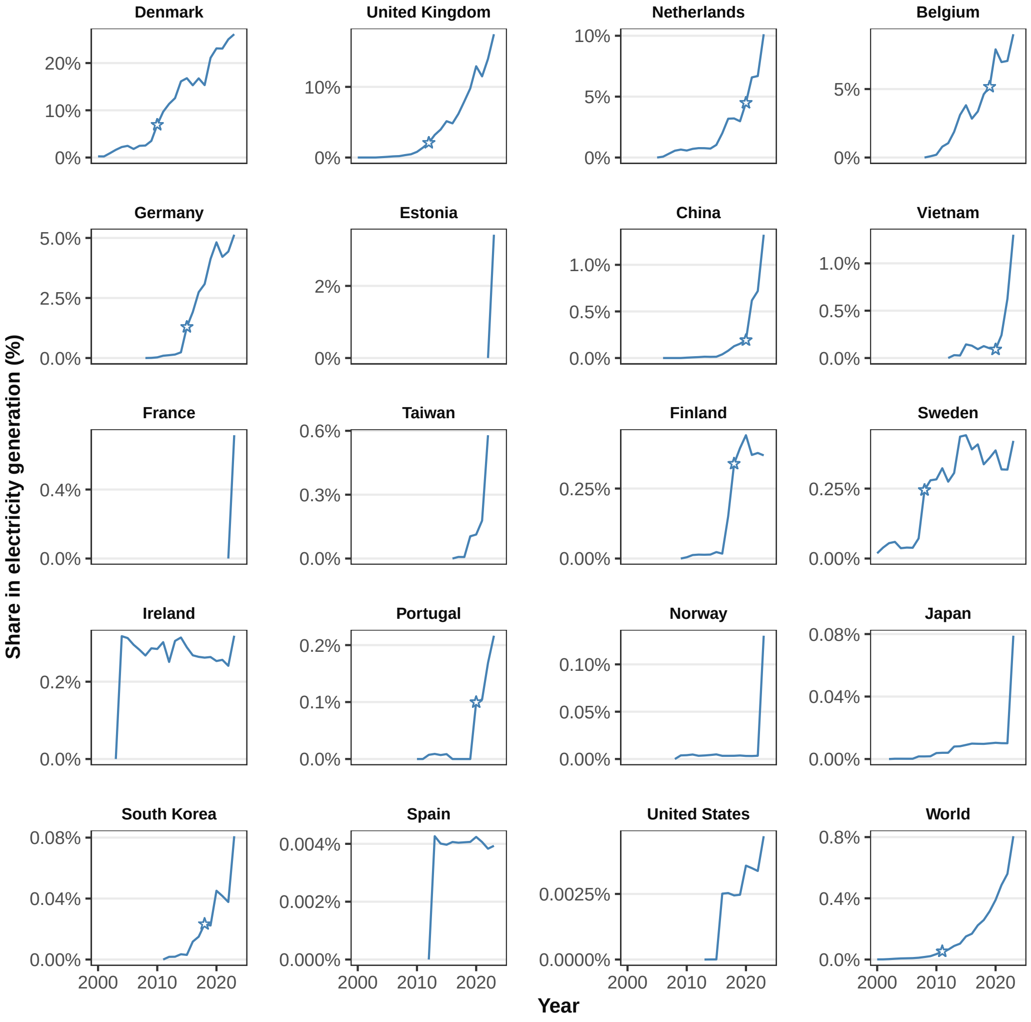

Supplementary Figure 14 Offshore wind rates
Bars show annual additions, solid lines show their three-year trailing average.

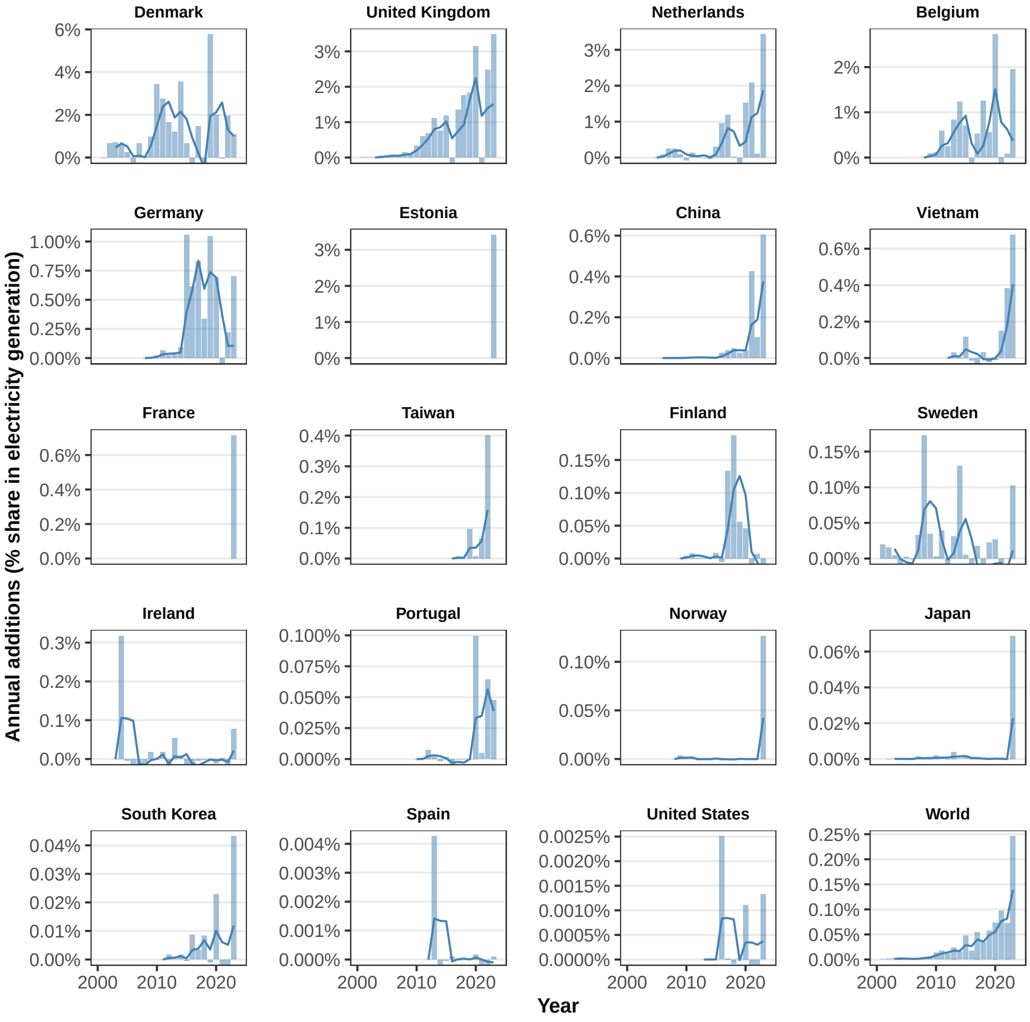

Supplementary Tables

Supplementary Table 1 Residual sum of squares (RSS) for logistic and Gompertz models fit to historical technology deployment time series.

Technology	RSS (Gompertz)	RSS (Logistic)	Best Fit	Ratio
CCGT	0.00606	0.00258	Logistic	2.35
Mobiles	0.0189	0.00310	Logistic	6.11
Nuclear	0.0215	0.0207	Logistic	1.04

Supplementary Table 2 Parameter configurations for onshore wind Monte Carlo simulations.

**Config**	**Growth rate (k)**	**Ceiling (L)**	**Takeoff year**	**Design type**
Base distribution parameters				
Base	$\Gamma(4.00, 11.43)$	$\Gamma(3.00, 120.00)$	$\mathcal{N}(5.5, 3.0)$	–
Grid exploration configurations				
1	0.17	0.14	5.5	Grid (low k, low L)
2	0.40	0.14	5.5	Grid (mid k, low L)
3	0.63	0.14	5.5	Grid (high k, low L)
4	0.17	0.37	5.5	Grid (low k, mid L)
5	0.40	0.37	5.5	Grid (mid k, mid L)
6	0.63	0.37	5.5	Grid (high k, mid L)
7	0.17	0.60	5.5	Grid (low k, high L)
8	0.40	0.60	5.5	Grid (mid k, high L)
9	0.63	0.60	5.5	Grid (high k, high L)
Face-centered configurations				
10	0.28	0.25	5.5	Face-centered (lower diagonal)
11	0.28	0.48	5.5	Face-centered (upper row)
12	0.52	0.25	5.5	Face-centered (right column)
13	0.52	0.48	5.5	Face-centered (upper diagonal)

Note: Base distributions are bounded ($0.01 \leq L \leq 0.95$, $0.05 \leq k \leq 1.00$). Parameter distributions are shifted to the means shown while maintaining the shape of the base distribution.

Supplementary Table 3 Parameter configurations for solar PV Monte Carlo simulations

Config	Growth rate (k)	Ceiling (L)	Takeoff year	Design type
Base distribution parameters				
Base	$\Gamma(4.00, 5.71)$	$\Gamma(3.00, 50.00)$	$\mathcal{N}(5.4, 1.98)$	–
Grid exploration configurations				
1	0.20	0.17	5.4	Grid (low k, low L)
2	0.49	0.17	5.4	Grid (mid k, low L)
3	0.79	0.17	5.4	Grid (high k, low L)
4	0.20	0.39	5.4	Grid (low k, mid L)
5	0.49	0.39	5.4	Grid (mid k, mid L)
6	0.79	0.39	5.4	Grid (high k, mid L)
7	0.20	0.60	5.4	Grid (low k, high L)
8	0.49	0.60	5.4	Grid (mid k, high L)
9	0.79	0.60	5.4	Grid (high k, high L)
Face-centered configurations				
10	0.35	0.28	5.4	Face-centered (lower diagonal)
11	0.35	0.50	5.4	Face-centered (upper row)
12	0.64	0.28	5.4	Face-centered (right column)
13	0.64	0.50	5.4	Face-centered (upper diagonal)

Note: Base distributions are bounded ($0.01 \leq L \leq 0.95$, $0.05 \leq k \leq 1.25$). Parameter distributions are shifted to the means shown while maintaining the shape of the base distribution.

Supplementary Table 4 Parameter configurations for mobile phones Monte Carlo simulations

Config	Growth rate (k)	Ceiling (L)	Takeoff year	Design type
Base distribution parameters				
Base	$\Gamma(4.00, 5.10)$	$\Gamma(3.00, 6.00)$	$\mathcal{N}(5.92, 2.68)$	–
Grid exploration configurations				
1	0.21	0.67	5.92	Grid (low k, low L)
2	0.52	0.67	5.92	Grid (mid k, low L)
3	0.83	0.67	5.92	Grid (high k, low L)
4	0.21	1.00	5.92	Grid (low k, mid L)
5	0.52	1.00	5.92	Grid (mid k, mid L)
6	0.83	1.00	5.92	Grid (high k, mid L)
7	0.21	1.33	5.92	Grid (low k, high L)
8	0.52	1.33	5.92	Grid (mid k, high L)
9	0.83	1.33	5.92	Grid (high k, high L)
Face-centered configurations				
10	0.36	0.83	5.92	Face-centered (lower diagonal)
11	0.36	1.17	5.92	Face-centered (upper row)
12	0.67	0.83	5.92	Face-centered (right column)
13	0.67	1.17	5.92	Face-centered (upper diagonal)

Note: Base distributions are bounded ($0.10 \leq L \leq 2.0$, $0.1 \leq k \leq 1.2$). Parameter distributions are shifted to the means shown while maintaining the shape of the base distribution.

Supplementary Table 5 Parameter configurations for CCGT Monte Carlo simulations

Config	Growth rate (k)	Ceiling (L)	Takeoff year	Design type
Base distribution parameters				
Base	$\Gamma(4.00, 10.00)$	$\Gamma(3.00, 12.00)$	$\mathcal{N}(6.0, 5.0)$	–
Grid exploration configurations				
1	0.19	0.33	6.0	Grid (low k, low L)
2	0.48	0.33	6.0	Grid (mid k, low L)
3	0.76	0.33	6.0	Grid (high k, low L)
4	0.19	0.50	6.0	Grid (low k, mid L)
5	0.48	0.50	6.0	Grid (mid k, mid L)
6	0.76	0.50	6.0	Grid (high k, mid L)
7	0.19	0.67	6.0	Grid (low k, high L)
8	0.48	0.67	6.0	Grid (mid k, high L)
9	0.76	0.67	6.0	Grid (high k, high L)
Face-centered configurations				
10	0.33	0.42	6.0	Face-centered (lower diagonal)
11	0.33	0.58	6.0	Face-centered (upper row)
12	0.62	0.42	6.0	Face-centered (right column)
13	0.62	0.58	6.0	Face-centered (upper diagonal)

Note: Base distributions are bounded ($0.01 \leq L \leq 1.0$, $0.1 \leq k \leq 1.2$). Parameter distributions are shifted to the means shown while maintaining the shape of the base distribution.

Supplementary Table 6 Parameter configurations for nuclear power Monte Carlo simulations

Config	Growth rate (k)	Ceiling (L)	Takeoff year	Design type
Base distribution parameters				
Base	$\Gamma(4.00, 10.00)$	$\Gamma(3.00, 12.00)$	$\mathcal{N}(6.0, 5.0)$	–
Grid exploration configurations				
1	0.19	0.33	6.0	Grid (low k, low L)
2	0.48	0.33	6.0	Grid (mid k, low L)
3	0.76	0.33	6.0	Grid (high k, low L)
4	0.19	0.50	6.0	Grid (low k, mid L)
5	0.48	0.50	6.0	Grid (mid k, mid L)
6	0.76	0.50	6.0	Grid (high k, mid L)
7	0.19	0.67	6.0	Grid (low k, high L)
8	0.48	0.67	6.0	Grid (mid k, high L)
9	0.76	0.67	6.0	Grid (high k, high L)
Face-centered configurations				
10	0.33	0.42	6.0	Face-centered (lower diagonal)
11	0.33	0.58	6.0	Face-centered (upper row)
12	0.62	0.42	6.0	Face-centered (right column)
13	0.62	0.58	6.0	Face-centered (upper diagonal)

Note: All configurations use bilogistic (100%) models. Base distributions are bounded ($0.01 \leq L \leq 1.0$, $0.1 \leq k \leq 1.2$). Parameter distributions are shifted to the means shown while maintaining the shape of the base distribution.

Supplementary Table 7 Regional deployment ceilings for onshore wind and solar PV in the acceleration scenarios.

Region	Onshore wind ceiling (%)	Solar PV ceiling (%)
East Asia	42.5	50
North America	45	45
South Asia	35	55
Europe	45	45
Asia-Pacific Developed	35	30
South-East Asia and developing Pacific	35	55
Africa	40	55
Eurasia	50	40
Latin America and Caribbean	35	45
Middle East	40	55

Note: Ceiling values represent maximum deployment potential as percentage of electricity generation.

Supplementary Table 8 Onshore wind deployment by acceleration scenario and region.

Scenario	Region	Deployment in 2050 (%)	Peak rate (p.p./yr)	Total growth (p.p.)	Growth share (%)
Baseline	*Global*	25.3	–	18.3	–
	Eastern Asia	28.9	1.32	–	42
	Europe	35.5	1.34	–	15
	Latin America and Caribbean	31.2	1.37	–	7
	North America	26.6	1.09	–	16
	South Asia	16.7	0.78	–	5
	Others	14.4	0.79	–	15
Early acceleration	*Global*	40.5	–	33.4	–
	Eastern Asia	42.5	1.84	–	37
	Europe	45.0	1.86	–	12
	Latin America and Caribbean	35.0	1.89	–	5
	North America	45.0	1.61	–	18
	South Asia	35.0	1.96	–	7
	Others	34.6	2.66	–	22
Late acceleration	*Global*	38.6	–	31.6	–
	Eastern Asia	42.5	2.18	–	39
	Europe	45.0	2.20	–	12
	Latin America and Caribbean	35.0	2.23	–	5
	North America	43.3	1.97	–	18
	South Asia	31.1	1.66	–	7
	Others	28.7	1.66	–	19

Note: Deployment and total growth shown as percentage of electricity generation; Peak rate shown as annual percentage point increase (p.p./yr); Growth share represents regional contribution to total global growth between 2023-2050.

Supplementary Table 9 Solar PV deployment by acceleration scenario and region.

Scenario	Region	Deployment in 2050 (%)	Peak rate (p.p./yr)	Total growth (p.p.)	Growth share (%)
Baseline	*Global*	20.8	–	15.2	–
	East Asia	22.2	1.22	–	38
	Europe	26.8	1.38	–	15
	Latin America and Caribbean	26.8	1.48	–	8
	North America	19.0	1.08	–	15
	South Asia	19.5	1.08	–	7
	Others	15.4	0.96	–	17
Early acceleration	*Global*	46.0	–	40.4	–
	East Asia	50.0	2.77	–	38
	Europe	45.0	2.82	–	11
	Latin America and Caribbean	45.0	2.83	–	6
	North America	45.0	2.72	–	16
	South Asia	51.9	2.72	–	9
	Others	39.3	2.31	–	19
Late acceleration	*Global*	46.7	–	41.1	–
	East Asia	50.0	3.00	–	38
	Europe	45.0	3.00	–	11
	Latin America and Caribbean	45.0	3.00	–	6
	North America	45.0	3.00	–	16
	South Asia	52.2	3.00	–	9
	Others	42.1	3.00	–	21

Note: Deployment and total growth shown as percentage of electricity generation; Peak rate shown as annual percentage point increase (p.p./yr); Growth share represents regional contribution to total global growth between 2023-2050.

Supplementary Notes

Supplementary Note 1: Technology growth and diffusion mechanisms and phases

The growth of new technologies is often represented as following an S-curve, where growth first accelerates, reaches a peak rate, and subsequently slows down and ends [1–3]. This mirrors the use of S-curves in disciplines like ecology and epidemiology and reflects the intuitive idea of early growth being driven by positive feedbacks before encountering increasing barriers and limits which ultimately prove unsurmountable.

The formative phase and technology take-off

Every new technology begins in the formative phase where its use is characterised by low deployment levels and erratic, unpredictable growth within a small set of niches [4–8]. Here, the growth and evolution of the technology is shaped by innovation, experimentation, and failures and as it "is tested, refined and adapted to market conditions" [9] with little interaction with the wider system. Together with the emergence of actor networks, business models and regulatory and policy environments, these developments allow the socio-technical regimes around the technology to facilitate consistent growth [5, 6, 10, 11].

This growth 'take-off' [6, 8, 12, 13] or "beginning of the movement" [1] signals the end of the formative phase and marks the first turning point in the technology's adoption. The existence of the formative phase has been documented at both the global [8] and country levels [6, 13]. At the global level it involves technology learning to reduce costs, standardisation of designs and establishing global supply chains, while at the country level it involves establishing necessary regulatory and market conditions suited to a specific national context.

The differences in when and which countries experience take-off illustrate the characteristics of the spatial diffusion of the technology [6]. However, while the literature proposes indicative thresholds for take-off – 2.5% [8] or between 0.3-1.8% [4] of the market, 100 MW installed capacity [14], 1% of total electricity supply [6, 15] – no take-off thresholds have been empirically established as yet. This presents a important challenge with regards to assessments of the role that emerging technologies might play in the future energy system. A technology that is beyond the formative phase in multiple markets may be judged to hold greater, more evidence-backed promise compared to one which isn't, but how do we make these assessments in the absence of a way to reliably measure when the formative phase ends? This also has implications for which policies are designed and implemented to accelerate

technological change. Supportive policies can play an important role in shortening the duration of the formative phase and enable earlier take-off within countries as well as faster cross-country diffusion.

Our contribution to this puzzle is two-fold: we first develop a method to empirically identify when different technologies take-off in different countries (Methods), and then use these estimates (Extended Data Figure 1) to measure how quickly technologies diffuse across countries (Extended Data Figure 2).

To measure this 'diffusion duration' we fit a logistic curve to the cumulative count for the number of countries where a technology has taken off and calculate $\Delta T_{\text{diffusion}} = ln(81)/k$, where k is the estimated growth constant. This metric indicates the years elapsed between the technology reaching take-off in 10% and 90% of countries. We show that while this cross-national diffusion took over half a century for older energy technologies like coal and hydropower, more recent (but still complex [16]) technologies like combined cycle gas turbines (CCGTs) and nuclear power have had shorter diffusion of durations of 36 and 30 years respectively. On the other end of the spectrum, mobile telephones, a granular [17], consumer-driven technology has had a diffusion duration of only 12 years. Solar PV and onshore wind fall in-between these cases with diffusion durations of 15 and 23 years respectively (Extended Data Figure 2).

The accelerating growth phase

After take-off, the technology enters the accelerating growth phase where its growth begins to follow an S-curve. The accelerating growth phase is characterised by a consistent increase in annual deployment additions driven by positive feedbacks from technology and policy learning, and increasing investment profitability. These 'increasing returns' [18] or 'cumulative causation' [19] may manifest as an increase in the profitability of installing and operating new technologies or political gains from extending support to them at the national level. At the global level they may result from increasing economies of scale for manufacturing and servicing artifacts for the new technology and an expansion in the number of countries adopting the technology.

Consistent with S-curve behaviour, technologies in the accelerating growth phase experience a consistent decline in the year-on-year relative growth rates even as the annual additions are expanding in absolute terms. The annual additions keep increasing until they reach their maximum rate – this marks the second turning point in the technology's adoption where the positive feedbacks driving accelerating growth are balanced out by countervailing barriers [6].

These barriers can take various shapes and forms and appear at different stages of the adoption process, with their cumulative impact intensifying with increasing deployment. They can include

resistance from incumbents, declining social acceptance and increasing public opposition, system integration challenges, land and other resource-use constraints, limits to supporting infrastructure, supply chain congestion, or limits to institutional capacity [20–33].

Growth pulses and the steady growth phase

It is typically assumed that a technology's growth immediately slows and approaches the saturation phase after it achieves its peak growth rate as the increasing barriers to adoption overpower the mechanisms driving it. At the end of the saturation the technology's growth grinds to a complete halt and stagnates at its peak market share or final deployment ceiling [34]. Following this point, the technology may maintain its market share until it begins to lose ground to an emerging alternative.

Contrary to this idea, we find that in some cases, the technology may experience a fresh 'pulse' of growth due to the availability of new markets, or the (re-)introduction of supportive measures. These growth pulses can be observed for several technologies at both the national and global scales. At the national scale, these pulses may be linked to the introduction of new policies, changing regulations, market reforms, financial support schemes, or due to other changes in the socio-technical landscape. At the global scale, they may result from market expansion as a technology diffuses to a new set of countries, or renewed growth in countries where growth had previously begun to slow or stagnate. For some technologies including nuclear power, coal and hydropower we see a renewed pulse of global additions due to delayed but large-scale adoption in Asia with China driving a substantial share of new growth (Extended Data Figure 3).

Together, the interplay of these mechanisms often culminates in a prolonged steady growth phase, with a balance between drivers and barriers yielding a period of nearly-linear growth. The presence of this steady phase hints at the role that targeted policy effort can play in sustaining growth over longer periods of time and delaying saturation. Policy effort can induce diffusion through cross-country technology transfers and knowledge spillovers, accelerate takeoff by helping curtail the duration of the formative phase, prolong the steady growth phase by introducing measures that address emerging barriers and delay saturation, and catalyse renewed growth pulses by creating new incentive structures.

In sum, we argue that the growth of a new technology proceeds through a sequence of four distinct phases – formative, accelerating growth, steady growth, and slow-down. These phases are repeated in every country at different points in time, and global deployment patterns emerge from the aggregate effects of national adoption. Initially global growth is largely influenced by technology take-off and acceleration in a small number of early adopters. Subsequently, it is shaped by asynchronicities

in cross-national diffusion, takeoff, acceleration, peaking and saturation which generate a myriad co-evolving feedbacks, synergies and dissonance.

The measurement of technology growth

Our analysis also shows that there are different metrics which can be used to measure technology adoption, and that the choice of metric influences the inferences one draws about the nature of a technology's growth. We measure technology growth in relation to the larger market – shares in electricity generation for solar and wind for which we have better generation data, shares in installed electricity generation capacity for other energy technologies, and share in population for mobiles.

Technology adoption is also often measured in absolute units (e.g. GW of solar capacity or MWh of solar electricity generation), but this ignores the crucial relationship between a technology and the market it operates in – when we measure adoption, a technology's market share is not only influenced by its own deployment growth dynamics but also those of the larger market within which it operates.

We show that the earliest electricity generating technologies based on coal, oil and hydropower were often responsible for creating a new market for electricity when they first began to be deployed. This led to their market shares starting close to 100% and then declining when other technologies entered the market (Supplementary Figure 1). Their deployment patterns are markedly different from later technologies like nuclear power that entered a pre-existing market for electricity and saw their shares gradually increase from zero. The example of nuclear power also illustrates how the size of the overall market impacts the characteristics of the technology's adoption curve – because of the relatively smaller size of the overall electricity system at the time of its adoption, even small increases in absolute deployment could translate to relatively big jumps in the technology's market share.

Thus, for many historical technologies, focusing on absolute versus relative adoption metrics would lead to significantly different inferences about the nature of growth. For more recent technologies like CCGTs, mobiles, solar PV and onshore wind, we see a near-perfect alignment between the patterns for the two, as absolute deployment went hand-in-hand with increasing market penetration amid relatively modest changes in the size of the overall market.

Supplementary Note 2: Mathematical models for technology growth

Technology S-curves are frequently represented using the logistic model [35], a three parameter function of the form

$$f(t) = \frac{L}{(1 + e^{-k(t-t_0)})} \tag{1}$$

with parameters k (the growth constant), L (the final deployment asymptote or 'ceiling'), and t_0 (the inflection point). Deployment achieves its maximum annual growth rate at the inflection point, which can be expressed as $G = \frac{Lk}{4}$ [6].

Logistic curves fit to empirical deployment data have been widely used for ex-post description and comparison of growth for historical technologies which have already reached their final ceilings[1–3, 36]. Such analyses are less suited to emerging technologies with expanding deployment due to the limited reliability of parameters estimated by fitting logistic functions to time series with continuing growth [6, 37, 38]

We posit that different parameters of the logistic curve reflect information about mechanisms shaping growth at different phases of the S-curve: the growth constant k captures the rate of deployment expansion during the accelerating growth phase, the inflection point t_0 marks the middle of the quasi-linear steady growth phase, and the deployment ceiling L indicates where growth finishes. Parameters reflecting later phases cannot be reliably estimated from early observations because these data are unable to represent all phases of technology growth – as they are only able to capture dynamics from a limited set of early phases, they do not have any information about the evolving mechanisms that shape later phases of growth. Thus, fitting a logistic function to data from the accelerating growth phase with consistently increasing annual additions might give us a reasonable estimate for k, but will only be able to guess at the values of t_0, or L as the underlying data does not have any information on the phases to come. The more data we have and the better its coverage of the whole deployment curve, the more reliable our parameter estimates.

We illustrate this phenomenon by fitting a logistic function to systematically truncated deployment time series for different technologies. For each technology, we track the evolution of the parameters estimated by fitting the curve to truncated data ending in a specific year. Our analysis shows that while the estimate for k stabilises fairly early (essentially as soon as the technology is nearing the end of the accelerating growth phase), the estimate for L is extremely unstable until very close to true saturation (Extended Data Figure 5). This 'moving L' phenomenon (Extended Data Figure 4), where the estimate for the ceiling regularly changes with increasing deployment makes it particularly difficult to use logistic fits to project future technology growth and often leads to overfitting (when the curve describes the data it is fit to reasonably well but is unable to accurately predict future growth).

Several different approaches have attempted to address this parameter estimation challenge. We see these as falling into two broad categories – one focusing on modifying the standard logistic curve

fitting approach, and another looking beyond the logistic function and introducing alternative growth models.

In the first category, ref. [39] suggest using an exogenously assumed, fixed value for L (aligning with a normative target or goal such as the 1.5ºC target) with a set take-off year and empirically derived estimates for the ‘emergence rate’ (which is analogous to the growth constant k) to generate a logistic curve. While the trajectories generated using this approach can be used to assess if growth is on track to reach the desired level by a certain time given a certain emergence rate, the projection is purely conditional on the choice of L and the emergence rate.

Another modelling framework from ref. [38] improves on this ‘fixed L’ approach by having L linearly increase with time at an exogenously defined rate. This formulation more accurately captures the ‘moving L’ phenomenon we highlight, but is again sensitive to assumptions about how fast L changes and at what level it peaks. Thus, while it is useful for constructing feasibility spaces for future growth under different assumptions about the emergence rate and the ‘demand pull’ raising L, it does not resolve the fundamental problem of empirically deducing L from early data.

The second category focuses on exploring alternatives to the logistic model, and has had a significantly longer history than the first.

Ref. [40] introduced the Gompertz model [41] to analyses of technology diffusion already in 1980. The Gompertz model, an asymmetric S-curve of the form

$$f(t) = Le^{-e^{-k(t-t_0)}} \tag{2}$$

with parameters k (the growth constant), L (the final deployment ceiling), and t_0 (the inflection point), has a longer growth phase with delayed saturation which mitigates some of the logistic model’s pessimism about the ceiling when used with earlier observations. However, it is also vulnerable to the same problem of being unable to reliably estimate parameters describing later phases from early data [6, 37].

Given the accelerating nature of growth early in the S-curve it is sometimes (implicitly [42–44] and explicitly [38, 45]) argued that the accelerating growth phase can be described by an exponential function. The exponential function is of the form

$$f(t) = y_i e^{K(t-t_i)} \tag{3}$$

where e is the exponential growth constant, and y_i is the value at initial time t_i. While it is quite obviously unsuitable for describing or projecting technology growth over the long-term due to its inherent inability to account for any barriers and slow-down, we also show that it is ill-suited to describing the accelerating growth phase because it does not capture the declining year-on-year growth rates characterising it; by definition, the exponential model assumes a fixed growth rate equal to the growth constant.

We propose that another model which can be used to describe the growth of policy-driven technologies is the bilogistic function [46]. The bilogistic curve is composed of two overlapping logistic curves and described by

$$f(t) = \frac{L_1}{1 + e^{-k_1(t-t_{01})}} + \frac{L_2}{1 + e^{-k_2(t-t_{02})}} \quad (4)$$

where L_1 and L_2 are the saturation levels for the first and second logistic curves, k_1 and k_2 their respective growth constants, t_{0_1} and t_{0_2} their respective inflection points.

To systematically assess the prevalence of multiple growth pulses, we conducted a comprehensive comparison of logistic versus bi-logistic model fits across national onshore wind and solar PV deployment time series. Growth pulses are identified by fitting both bilogistic and logistic models to the same empirical data and comparing goodness of fit using the Akaike Information Criterion (AIC), which penalizes the bilogistic model for its additional parameters. We define a ‘good’ bilogistic fit as one that depicts actual peaks of growth rate separated by a ‘trough’ where the growth rate is at least 10% or 30% slower than the rate at the lowest of the two peaks, with neither logistic component depicting decline. We find that wind and solar power show substantially high shares of bilogistic best fits: 71% of onshore wind deployment series (15 of 21 eligible cases) and 62% of solar PV series (21 of 34 cases) are better represented by bilogistic functions under the 10% trough criterion. Even with the more stringent 30% criterion, nearly half of wind (48%) and solar (47%) deployment series exhibit clear multi-pulse patterns. In addition to renewables, we also find evidence for bilogistic growth in national nuclear power, mobile telephone, and CCGT deployment. At the global level, only nuclear power exhibits a good bilogistic fit, where the growth rate trough is 11% lower than the lower peak, while neither wind nor solar show clear global bilogistic patterns despite their prevalence at national levels. These findings provide strong empirical justification for incorporating bi-logistic functions in modeling renewable energy diffusion, with the caveat that while the bilogistic model may be good at describing past growth, it may be susceptible to over-fitting and fail to reliably anticipate future growth.

Recent studies [47, 48] have demonstrated yet another approach which attempts to mitigate the deficiencies of individual models by using several different models and averaging projections from across the ensemble. They fit different growth models to empirical data, generate projections, use hindcasting to evaluate the performance of each model, and then generate weighted projections where better performing models are assigned higher weights. While the use of a more diverse ensemble of models coupled with iterative hindcasting makes this approach less vulnerable to overfitting compared to projections based on a single model fit to a single time series, it still faces the same underlying problem of estimating parameters corresponding to later phases using early data.

We argue that this parameter estimation problem is not limited to a particular growth model but extends to the broader enterprise of using models fit to empirical data to make projections.

Supplementary Note 3: Projecting global technology growth using national-level data

Aggregating projections for individual countries

Models fit to a single set of historical observations are implicitly constrained in their ability to anticipate future technology growth dynamics. One way to try and address this limitation is to adopt a more bottom-up approach, and project global deployment by aggregating projections for the growth in individual countries.

Conceptually, this is quite logical – global deployment is essentially the aggregate of country-level developments, and in the event of global data not yielding reliable projections, another approach could use all available national data to make projections for individual countries and then sum them up.

We experiment with this approach, and fit logistic curves to all national time series with 5 or more non-zero datapoints. We discard those cases where the fitting algorithm fails, or where the estimated growth asymptote L is deemed too high (>100% for onshore wind, solar PV, CCGT, nuclear; >200% for mobiles). We extrapolate each national curve into the future, and aggregate all available national curves to derive a projection for global deployment.

The global deployment at year t is given by $f(t) = \sum(w_i * d_i(t))$ where w_i is country i's share in the global market and d_i its projected deployment in year t.

To assess the reliability of this approach, we systematically truncate our national time series and hindcast the growth of different technologies from different years in the past and measure the

resulting trajectories' ability to describe both, in-sample and out-of-sample global deployment (see step 5 in Supplementary Note 4 for a detailed description of our hindcasting approach).

We find that the approach performs poorly at projecting out-of-sample deployment, especially early in the diffusion process. This happens because of two main reasons.

First, technology growth does not start simultaneously in all countries. This means that the national sample being used to generate the global projections does not cover a sufficiently large share of the global market. We measure the coverage of the sample in a given year as $\sum w_i$ where w_i is country i's share in the global market in that year. In the absence of a sufficiently large sample, the national projections made using this approach struggle to add up to the in-sample global deployment, let alone reliably estimate future growth.

Second, the general limitations of approaches based on fitting curves to time series data we highlight in Supplementary Note 2 also extend to this approach centred on national data. It is difficult to reliably estimate S-curve parameters, especially the growth asymptote (L), from empirical observations capturing earlier stages of technology growth. Incorrect estimates for these parameters yield extrapolations that perform poorly at predicting future deployment. Thus, this approach focusing on aggregating national projections to derive global projections using early national data often accumulates large errors.

Moreover, such an approach also yields a single, deterministic projection for global deployment that is in turn based on similar projections for individual countries.

In principle, the approach could be modified to produce probabilistic estimates instead, with global deployment projections aggregating probabilistic projections across individual individual countries.

Recent studies [47, 48] have pioneered an approach for producing probabilistic projections for the growth of different energy technologies in Swiss municipalities and different European countries. They also demonstrate a method for producing probabilistic projections for Switzerland as a whole by aggregating probabilistic projections for individual municipalities. However, they also flag the problem we highlight above, wherein different municipalities/countries adopt different technologies at different times, and thus there are many municipalities/countries for which there is not enough data to make a projection. The applicability of such an approach to generating probabilistic projections for global deployment is thus constrained by data limitations not only for fitting curves, but also for performing the iterative hindcasting required to generate the probabilistic distributions.

As of 2023, there were only 30 and 17 countries for onshore wind and solar PV respectively, for which we have at least 10 post-takeoff datapoints, the bare minimum we would need for in-sample fitting (5 datapoints) and out-of-sample validation (5 datapoints). Together these represent 75% of global electricity generation for onshore wind, and only 37% of global electricity for solar PV, with coverage dropping sharply for earlier years e.g. 18 wind countries covering 40% in 2016; just 6 solar countries covering 5%). This lack of historical depth not only limits the construction of robust global aggregates but also makes it impossible to rigorously hindcast and verify such a method.

However, this does not preclude the possibility of us making meaningful projections altogether as despite lags in cross-country diffusion, there are individual countries (which we call 'frontrunners') where both solar PV and onshore wind are already at more advanced phases of adoption [6].

Using national parameter distributions to project ranges for global growth

Introducing the idea of growth phases and frontrunner countries to time series forecasting facilitates yet another approach to project global deployment using national observations. This approach uses distributions of national growth parameters to inform estimates of global growth rather than aggregating projections for individual countries.

Building on discussions in Supplementary Notes 1-2, we posit that even though deployment data from these frontrunner countries do not yet capture slow-down and saturation, they offer us an empirical window into studying the evolving balance of drivers and barriers across the accelerating and steady growth phases.

We find that statistical ranges (median and IQR) for the growth constant (k) and peak growth rate (G) from logistic curves fit to national data frequently encompass the corresponding estimates for logistic curves fit to global data, and can thus help anchor expectations for the eventual values of these parameters for the world as a whole (Extended Data Figure 5). At the same time, our analysis also shows that similar statistics for L at the national scale are not informative for estimating L at the global scale.

However, in order to use these data to project global deployment, we need to identify the mathematical relationship between national parameter statistics measured at earlier points in time and the parameters of a curve describing the completed global deployment trajectory. This presents the same problem contemporary approaches struggle with – in the absence of a crystal ball that shows us data from the future, we have no way of knowing what the parameters of the eventual global trajectory will be.

One way to overcome this challenge would be to study historical technologies for which we have data spanning the whole S-curve and use them as reference cases. We could quantify the relationships between early national curve parameters and final global curve parameters, and then use them with other technologies. But this approach also presents a challenge – how do we know if the relationships measured for one reference technology also hold for another? Given the specificity of technological characteristics and the socio-political, economic context in which each technology is deployed, it is difficult to guarantee that historical patterns for one technology can be used to predict those for another. For instance, if we want to project the future deployment of solar power, is it likelier to follow patterns observed for other energy technologies like nuclear power or CCGTs during the 20th century, or for granular consumer technologies like mobiles in more recent years?

Yet another challenge relates to the actual process of quantifying these relationships between national and global parameters – what methods can we use to capture these complex, non-linear relationships that might vary in shape and form across different technologies?

We resolve these challenges through PROLONG – a probabilistic modelling approach.

Supplementary Note 4: PROLONG (PRobabilistic mOdeL Of techNology Growth)

PROLONG uses computational simulations to explore different possibilities for the growth and diffusion of a given technology in an ensemble of virtual worlds, and uses a machine learning model to capture the quantitative relationships between parameters describing incomplete national growth and completed global trajectories.

We argue that a diverse enough possibility space composed of thousands of simulated global trajectories, each of which is the aggregate outcome of a unique set of national growth dynamics, captures adoption patterns similar to those unfolding in the real world, as well as those where the technology is more/less successful. In generating these simulations, we make explicit assumptions about what we see as a plausible range for the takeoff timing, for the shape and form of national growth trajectories, and for permissible deployment speeds and ceilings. Our approach uses computational simulations to train a machine learning model to recognise the relationships between early national growth parameter statistics and the parameters of a technology's final global trajectory, and then use the trained model to make probabilistic projections for global growth using empirical national data.

The implementation of our modelling framework consists of the following steps:

- **Step 0:** Defining rules for the virtual worlds

- **Step 1:** Exploring diverse technology futures using Monte Carlo simulations
- **Step 2:** Generating training data from the ensemble of simulated trajectories
- **Step 3**: Using machine learning to identify the relationships between truncated national and final global parameters
- **Step 4:** Generating probabilistic projections from empirical data
- **Step 5:** Model validation and hindcasting

Step 0: Defining rules for the virtual worlds

Before we start generating simulations we define a set of core assumptions that form the basic structure for the model.

Our simulation module represents a virtual world composed of 150 countries, with the distribution of their relative, technology-specific market sizes carefully calibrated to reflect real-world market sizes. We quantify the market shares as normalised shares (summing to 1). For solar PV, onshore wind and nuclear power, market shares are derived from national total electricity generation, reflecting each country's relative electricity system size. For mobile phones, market shares are proportional to population counts, representing the potential user base. For CCGTs, market shares are calculated based on each country's existing natural gas electricity generation capacity. These market shares directly weight each country's contribution to global aggregate deployment. For example, a country with 5% of global electricity generation would contribute five times more to global solar deployment than a country with 1% generation share, assuming identical percentage-based deployment within each country. This market-weighted approach ensures our simulations properly account for the outsized influence of large markets on global technology diffusion patterns, while still capturing the diversity of growth dynamics across different national contexts.

We simulate technology growth in each country separately, and global deployment at each time step is the weighted sum of all national deployment at that instant.

We assume that each country can follow trajectories modelled by one of two shapes – a standard S-curve (using a logistic function) or a curve with two pulses (using a bi-logistic function). For the logistic trajectories, we need to assign each country a takeoff year (the year when deployment first exceeds 1%), intrinsic growth rate (k) and deployment ceiling (L), after which we can calculate the inflection point using the equation $t_0 = \text{takeoff}_{\text{year}} + (1/k)log(L/0.01 - 1)$, where t_0 is the inflection point year. For the bi-logistic trajectories, we need to assign each country a takeoff year, intrinsic growth rate for the first pulse (k_1) and the final deployment ceiling (L). The final ceiling is split

between the two pulses with respective ceilings L_1 and L_2 using a random fraction between 20-80%. We calculate the inflection point for the first pulse (t_{0_1}) and its peak annual growth rate (G_1). The second pulse starts after a random delay between 1-10 years after t_{0_1}, and is assigned a peak annual growth rate (G_2) calculated by multiplying G_1 by a random multiplier that is centred around 1 – this allows for the equal possibility of the second pulse exhibiting faster/slower growth than the first. The intrinsic growth rate (k_2) for the second pulse is calculated as $k = 4G2/L2$, and its inflection point (t_{0_2}) as $t_{0_2} = \text{takeoff}_{\text{year}_2} + (1/k2)log(L2/0.01 - 1)$. The choice of a 1-10 year delay for the second pulse allows for both quick transitions between pulses (1 year) and longer pauses between growth phases (up to 10 years).

Real-world technology deployment rarely follows perfect (bi-)logistic curves due to policy changes, economic fluctuations, supply chain disruptions, and other temporal factors. To replicate this variability, we implement a correlated noise model with three parameters. First, we apply multiplicative noise with an initial amplitude of 5% of the current deployment value. This ensures deviations scale proportionally with deployment levels – smaller variations in early adoption phases and larger absolute fluctuations during rapid growth periods. Second, we implement year-to-year correlation ($\rho = 0.7$) to create persistent effects that mimic how real-world drivers and barriers typically influence deployment over multiple consecutive years. For instance, the implementation of a new policy might affect deployment over several years rather than causing independent annual fluctuations. Third, we incorporate amplitude decay (2% annual reduction from the initial 5%) to reflect how mature markets tend to demonstrate more steady, predictable growth with reduced volatility. This three-component noise structure creates trajectories with realistic short-term variability while preserving the underlying diffusion pattern, avoiding both the artificial smoothness of perfect curves and the unrealistic randomness of uncorrelated noise.

Here is an example to illustrate how the noise works. Let's consider a country with 10% deployment in year t. With a 5% noise amplitude, the deployment value might be adjusted to $10\%(1+0.05) = 10.5\%$ or $10\%(1-0.05) = 9.5\%$, depending on the random noise value. In year $t+1$, if deployment grows to 15% and the previous year's noise was +5%, the correlated noise would be calculated as $0.7(+5\%) + 0.3$ (the new random noise). If the new random component is -3%, then the noise in year $t+1$ would be $0.7(+5\%) + 0.3(-3\%) = +2.6\%$. The deployment would then be adjusted to$15\%(1 + 0.026) = 15.39\%$. By year $t + 5$, the noise amplitude would have decayed to $5\%(0.98)^5 \approx 4.5\%$, reducing the magnitude of potential fluctuations as the market matures.

We introduce spatial heterogeneity in when growth starts, how fast it happens, and where it ends by assigning countries different parameters from different statistical distributions such as normal or gamma distributions which vary between technologies. We use a Monte Carlo simulation engine to simulate technology growth in thousands of virtual worlds, where each country follows a new, unique trajectory every single time, leading to the emergence of a diverse set of global growth patterns. The Monte Carlo simulation is implemented using R's parallel processing capabilities through the 'parallel', 'foreach', and 'doParallel' packages, allowing for efficient computation across multiple CPU cores simultaneously.

We simulate technology growth and diffusion over a time period of 50 years; long enough for countries to move from the formative to slow-down phases.

Step 1: Exploring diverse technology futures using Monte Carlo simulations

Establishing parameter distributions

To start generating simulations, we first define the distributions which control the national growth dynamics.

We first look at early empirical data for the technology – up to 2015 for solar PV, 2010 for onshore wind, 1998 for mobiles, 1985 for CCGTs and 1960 for nuclear power – and fit normal distributions to the available national takeoff years, and gamma distributions to logistic k and logistic L for mature countries (with logistic curve maturity $\geq 50\%$). These distributions inform our priors about national curve parameters for the specific technology at an early stage of adoption and define our 'base configuration'. Given what we know from empirical analyses of technology growth, these distributions most certainly do not capture the 'true' parameter space for the eventual growth of the technology and very likely underestimate its full potential. The gamma distribution is chosen for modelling k and L parameters because it naturally constrains values to be positive while allowing for right-skewed patterns.

We address this limitation by first defining a broader range of plausible values for the takeoff year, k and L for each technology (with the specific aim of accounting for a diverse set of diffusion patterns) and then systematically shifting the distributions for each parameter to explore different regions of the technology's possibility space. We use a structured approach that ensures thorough coverage of parameter combinations—creating worlds where technologies grow quickly but saturate at low levels, others where growth is slower but reaches higher penetration levels, and various combinations

in between. For example, one configuration might combine higher k values with moderate L, while another might pair slower k with higher L (Supplementary Figure 2, Supplementary Tables 2-5).

We implement this parameterization using a hybrid coverage approach that combines a full factorial design with face-centered points to efficiently explore the parameter space. Our code creates a grid of parameter values by dividing each parameter range (k, L, and takeoff year) into multiple divisions and selecting points at these positions. For example, with $k_{\text{divisions}} = 3$, we sample 3 different mean values for the gamma distribution of k, from lower to higher growth rates. Consider two adjacent grid points in our parameter space: one with $k_{\text{mean}} = 0.2$, $L_{\text{mean}} = 0.4$ and another with $k_{\text{mean}} = 0.3$, $L_{\text{mean}} = 0.6$. The face-centered approach adds an additional point between them at $k_{\text{mean}} = 0.25$, $L_{\text{mean}} = 0.5$. This ensures we capture not just corner cases but also intermediate parameter combinations, providing more comprehensive coverage of the parameter space. For each configuration, we adjust the underlying distribution parameters (shape and rate for gamma distributions, mean and standard deviation for normal distributions) to achieve the target mean values while maintaining appropriate dispersion. Setting $k_{\text{divisions}} = 3$, $L_{\text{divisions}} = 3$ with a single distribution for takeoff timing with this approach generates 13 distinct configurations that systematically explore different combinations of fast/slow growth, and low/high saturation levels (Supplementary Figure 2).

Running Monte Carlo simulations

We generate 13 different configurations and then use each one to simulate 1000 different technology diffusion pathways. For each configuration, we run a Monte Carlo simulation engine which randomly assigns parameter values to individual countries by drawing from the distributions defined in this configuration. Each country is assigned its own takeoff year, intrinsic growth rate (k) and deployment ceiling (L), which are then used to generate its deployment trajectory. We account for different market sizes by weighting each country's contribution proportionally to its share of the global market, simulating how technologies might spread differently in large versus small countries.

These country-level trajectories are then aggregated into a global adoption curve for each simulation run. By repeating this process 1000 times for each configuration, we generate a dataset of 13,000 plausible global diffusion trajectories—some showing rapid global adoption, others displaying more gradual growth patterns, and many exhibiting complex multi-phase growth.

In addition to exploring the possibility space by varying the parameter configurations, we also run these simulations under differing assumptions about the shape of national growth trajectories. We run three different batches of simulations – one where all countries have logistic growth, one where all countries have bi-logistic growth, and one where each country has an equal chance of following

logistic or bi-logistic growth and there's an even split in the number of countries following either pattern in each run. Subsequent steps involving the generation of training data and producing a trained machine learning model are followed separately for each batch – see Step 4 for more details.

We can make our coverage of the possibility space more comprehensive by running a larger number of simulations and using more granularly differentiated configurations, but each addition comes at the cost of significantly higher computational requirements.

To make subsequent analysis more computationally tractable, we apply a randomised filtering process to select a smaller subset of simulations for our final training dataset. For each parameter configuration, we randomly sample over a third of the runs (35%) from the pool of simulations. This approach ensures our training dataset maintains the diversity of possible diffusion patterns while substantially reducing its size – from 1000 total runs down to 350 selected runs per configuration for solar PV and onshore wind.

The selection of 35% of the total runs per configuration was determined through experiments balancing computational efficiency with maintaining diversity – we found this range provided sufficient pattern coverage while substantially reducing computation time for subsequent model training.

Step 2: Generating training data from the ensemble of simulated trajectories

For the final step in our data preparation, we transform our simulation data into a format that teaches the model how to make predictions with limited information. Here, we systematically truncate the national deployment data from each simulation run at various early years (years 12, 15, 18, 24, 30, and 35) to create snapshots of what the diffusion pattern would look like if observed at those points in time. This exercise mimics the real-world challenge of forecasting from partial historical data. For each truncated snapshot, we fit logistic growth curves to the truncated national-level data, extracting parameters that characterise the diffusion process in each country up to that point. These include median, Q1 and Q3 for k and G, as well as the number of "mature" countries in the steady growth phase and their combined market share. We only use observations from those countries where the logistic curve maturity is identified as being over 50%.

The parameter extraction process employs a curve-fitting procedure implemented adapted from ref. [6]. For each country with sufficient data points (at least 5) and meaningful deployment ($\geq 1\%$), we fit a logistic curve and extract the key parameters. We then calculate distributional statistics (median, first quartile, third quartile) across all mature countries to characterise the overall pattern of

national growth. This approach captures both the central tendency and variation in growth patterns among early adopters.

We then pair these early-stage national parameters for each simulation run with the known parameters of a logistic curve fit to its full global trajectory, creating training data that connect "what we know so far" at the national level with "what eventually happens" at the global scale. We feed this data into a quantile random forest model – an ensemble learning method that builds hundreds of decision trees, each of which learns decision rules that connect early-stage national diffusion patterns to their ultimate global outcomes.

Step 3: Using machine learning to identify the relationships between truncated national and final global parameters

We implement the quantile random forest using the 'ranger' package in R, a high-performance implementation of random forests particularly well-suited for large datasets. Our model uses 1000 trees, which we found sufficient to achieve stable predictions while balancing computational efficiency. We enable the "quantreg" option in ranger to estimate conditional quantiles rather than just the conditional mean. The importance of features is calculated using the impurity-based method, allowing us to identify which national parameters most strongly influence global outcomes.

The forest's structure captures the complex, non-linear relationships between early diffusion signals and long-term outcomes, while being robust against outliers and noise. Unlike standard regression models that predict only mean outcomes, our quantile forest approach estimates the entire conditional distribution of possible futures.

We train two separate models – one that uses early national data to predict the global k, and another which uses the same data to predict the global G. By training on a diverse set of simulated histories with known outcomes, the models develop an understanding of how partial patterns tend to evolve, enabling them to look at real-world data up to the present day and make informed projections about the full spectrum of likely future evolutions—from conservative lower bounds to ambitious upper estimates.

Step 4: Generating probabilistic projections from empirical data

To use the model to make projections, we prepare input data capture the median, Q1 and Q3 k and G for mature logistic fits to empirical national data, the number of mature countries in the sample, their combined global market share, and the current global deployment level.

The model takes these inputs and predicts the quantiles for k and G, which are then used to derive a value for the saturation level (L). Note that the random forests are trained to predict parameters that capture the speed of growth during the accelerating growth and steady growth phases, and not the deployment ceiling — we only derive L from these parameters in order to be able to generate a full curve. We consider all possible combinations of k and G quantiles (5x5=25 combinations), and filter out those combinations where $L \leq$ the current deployment. Each combination is then used to generate a complete logistic curve, with the inflection point calculated using the current global deployment (y_i) and current year (t_i) using the equation$t_0 = ((1/k) * log((L/y_i) - 1)) + t_i$, where t_0 is the inflection point year, k is the growth rate, L is the saturation level, y_i is the current deployment, and t_i is the current year.

For each valid k-G combination, we generate a full trajectory up to the desired projection horizon. The global growth constant (k) directly influences how quickly deployment accelerates and then decelerates, while the peak annual growth rate (G) helps determine the saturation level (L) through the relationship $L = 4G/k$. This approach captures the mathematical relationship between these parameters while ensuring consistency with current observed deployment levels.

To identify the most representative trajectory for each quantile level (5th, 25th, median, 75th, and 95th), we calculate the area difference between each generated trajectory and the year-by-year quantile values derived from all valid trajectories. The trajectory with the minimum area difference becomes the representative curve for that quantile, providing a coherent set of parameters that best represents that particular future pathway. This area-based matching ensures that the representative trajectories maintain consistent parameter relationships while closely tracking the statistical properties of the full ensemble.

For each year in the projection horizon, we collect the predicted deployment values from all valid trajectories and calculate summary statistics across the full set which gives us a year-by-year probability distribution for future global deployment.

Our probabilistic framework explicitly acknowledges multiple dimensions of uncertainty. First, the use of quantile random forests captures the uncertainty inherent in technology diffusion processes—the natural variability observed even when initial conditions are similar. Second, by generating projections from diverse parameter combinations rather than single parameter values, we address parametric uncertainty about the true values of growth parameters. Finally, by training separate models on different trajectory shapes (logistic, bilogistic, and mixed), we incorporate structural uncertainty about the underlying model form. This comprehensive treatment of uncertainty provides

a more realistic view of possible futures than deterministic approaches or those that address only a single dimension of uncertainty. See Supplementary Note 5 for a detailed assessment of uncertainty in PROLONG.

Step 5: Model validation and hindcasting

To validate the predictive capabilities of our approach, we implement a comprehensive hindcasting framework that systematically evaluates how well our models can project known historical deployment patterns from earlier, incomplete data.

We perform a series of hindcasting tests to evaluate the performance of our projection model. We do so by using the model with both, simulated and empirical national data truncated at different years in the past, and then comparing the resulting projections to 'out-of-sample' global data.

Our hindcasting approach follows these steps:

1. For each technology, we select multiple historical cutoff years (e.g., 2005, 2010, and 2015 for solar PV)
2. For each cutoff year, we extract national deployment data up to that year only
3. We apply our model to this truncated data to generate projections
4. We compare these projections against the actual observed deployment in subsequent years
5. We repeat this process for models trained on logistic, bilogistic, and mixed trajectory data

This process mimics real-world forecasting scenarios and provides an objective assessment of each model variant's predictive performance.

To estimate the absolute error, we use the symmetric mean absolute percentage error (sMAPE) [**?**] which quantifies the absolute magnitude of point errors (thereby avoiding the cancellation of negative and positive errors) and also accounts for the relative scale of the quantity being measured (thereby avoiding asymmetricity). We use it to assess the overall out-of-sample forecasting performance for each model when informed by in-sample observations until year z:

$$sMAPE = \frac{1}{t_{end} - z} \sum_{i=z}^{t_{end}} \frac{|y_z - f(z)|}{(|y_z| + |f(z)|)/2} * 100 \tag{5}$$

To estimate the directional error, we calculate the symmetric mean percentage error (sMPE) [**?**] which quantifies both the magnitude and direction of the point errors and avoids asymmetricity and large errors (when the out-of-sample values are close to zero). We use it to assess the tendency of a model informed by in-sample observations until year z, to over- or under-predict out-of-sample deployment:

$$sMPE = \frac{1}{t_{end} - z} \sum_{i=z}^{t_{end}} \frac{y_z - f(z)}{y_z} * 100 \qquad (6)$$

To measure the skill of our probabilistic projections we use the Continuous Ranked Probability Score (CRPS) [49] measures "how well the marginal distributions of the forecast represent the ground truth" [50]. To test the calibration of the probabilistic intervals we use other metrics including interval widths [51], interval scores [52], and relative uncertainty [51] (these are described in detail in Supplementary Note 5).

To evaluate the performance of our quantile random forest approach, we conducted out-of-sample testing using held-out simulation data across five diverse technologies: solar photovoltaic, onshore wind, combined cycle gas turbines (CCGT), mobile phones, and nuclear power. We generated probabilistic projections for global deployment and compared these against known simulation outcomes across multiple forecast horizons and data truncation points. The results demonstrate that our machine learning model exhibits predictable and well-calibrated performance characteristics across this diverse technology portfolio (Supplementary Figures 3 and 4). For our primary technologies of interest – solar photovoltaic and onshore wind – forecast accuracy measured by the symmetric Mean Absolute Percentage Error (sMAPE) exhibits a consistent degradation pattern, starting from approximately 2% at 1-year forecast horizons and increasing systematically to around 12% at 30-year horizons. This degradation follows a near-linear trend, with intermediate error levels of approximately 4-6% at 10-year horizons and 8-10% at 20-year horizons. The Continuous Ranked Probability Score (CRPS) correspondingly increases from approximately 0.005 at short horizons to 0.025-0.030 at 30-year horizons, indicating that while probabilistic forecast skill degrades with forecast distance, it remains within acceptable ranges for practical applications.

All five technologies exhibit remarkably similar performance patterns in terms of forecast accuracy degradation, though with notable technology-specific variations in absolute performance levels and uncertainty characteristics. CCGT demonstrates marginally higher forecast errors across all horizons, with sMAPE values reaching approximately 15% at 30-year horizons compared to 12% for renewable technologies. Nuclear power displays the most distinctive pattern, exhibiting substantially wider uncertainty intervals as measured by the 90% interval width (reaching 0.6-0.7 at long horizons compared to 0.4-0.5 for other technologies) and higher relative uncertainty levels that can exceed 1.0 at extended forecast horizons, indicating that prediction intervals become larger than the central forecasts themselves.

The uncertainty quantification metrics reveal systematic patterns in forecast reliability. The 90% interval width increases progressively from approximately 0.1 at short horizons to 0.4-0.6 at 30-year horizons across most technologies, while relative uncertainty grows from near-zero to 0.5-1.5 depending on the technology. These patterns demonstrate that PROLONG's probabilistic framework appropriately captures increasing uncertainty with forecast distance while maintaining practically useful precision for medium-term planning horizons of 10-20 years. Most importantly, the symmetric Mean Percentage Error (sMPE) remains consistently near zero (within ±0.05 or 5%) across all forecast horizons and technologies, indicating an absence of systematic bias in PROLONG's projections. This unbiased performance represents a critical advantage over traditional curve-fitting approaches that often exhibit substantial directional bias, particularly at longer forecast horizons. The consistent near-zero bias across simulated validation tests provides strong evidence that PROLONG's machine learning methodology successfully captures the underlying diffusion dynamics without introducing systematic over- or under-prediction tendencies.

The marked consistency in performance patterns across such diverse technologies—spanning energy generation (solar, onshore wind, CCGT, nuclear) and information technology (mobile phones)—provides strong evidence for the robustness of our modelling approach. These validation results confirm that our quantile random forest successfully captures the complex, non-linear relationships between early national deployment patterns and eventual global outcomes while providing realistic uncertainty bounds that appropriately widen with increasing forecast horizons. The consistent degradation patterns and absence of systematic bias across this diverse technology portfolio provide confidence in the model's general applicability for generating probabilistic technology diffusion projections from limited empirical data.

We then evaluate the performance of the quantile random forest with empirical data. First, for each technology, we compare the performance of the models trained on logistic, bilogistic and mixed data to identify which model most accurately captures empirically observed growth dynamics. We identify the best performing models for onshore wind (bilogistic data), solar PV (mixed data), mobiles (logistic data), CCGTs (bilogistic data), and nuclear (bilogistic data) and use these models for subsequent tests and comparisons.

The selection of the best model variant for each technology is based on an evaluation of sMAPE, sMPE and CRPS scores and prediction interval characteristics (Supplementary Figure 5). We found that technologies with more complex adoption patterns (like onshore wind, which often shows multi-phase growth) are better captured by the bilogistic model, while technologies with smoother adoption

curves (like mobile phones) are better represented by the simpler logistic model. For solar PV, the mixed model performed best, suggesting its deployment patterns so far show characteristics of both single-phase and multi-phase growth across different countries.

The empirical validation results demonstrate the superior performance of PROLONG compared to conventional forecasting approaches across all five technologies (Extended Data Figure 6; Supplementary Figure 6). When evaluated against exponential global fits, logistic global fits, and logistic national aggregation methods using real-world deployment data, PROLONG consistently achieves lower forecast errors and exhibits substantially reduced systematic bias. Across forecast horizons ranging from 1 to 30 years, PROLONG maintains sMAPE values typically below 0.5 for most technologies, while alternative methods often exceed 1.0, particularly for longer forecast horizons. Most importantly, PROLONG's sMPE values remain consistently near zero across all technologies and forecast periods, indicating minimal systematic bias, whereas conventional approaches frequently exhibit substantial positive or negative bias that worsens with forecast distance. The performance advantage is particularly pronounced for our primary technologies of interest – solar PV and onshore wind – where PROLONG's accuracy improvements are most evident. These empirical results validate that PROLONG's machine learning approach, which uses patterns from early national adoption to inform global projections, significantly outperforms traditional curve-fitting methods that rely solely on historical global time series or simple national aggregation approaches, providing both superior accuracy and more reliable uncertainty quantification for technology forecasting applications. However, it is also important to note that a conventional curve fitting approach begins to offer higher out-of-sample projection accuracy once a technology enters the saturation phase and approaches its final deployment ceiling; this is because at this stage of the diffusion process, the in-sample time series has enough information to correctly estimate L.

Supplementary Note 5: Treatment and measurement of uncertainty in PROLONG

This Supplementary Note examines how PROLONG addresses various sources of uncertainty in technology diffusion projections and presents uncertainty quantification, performance and the natural boundaries for probabilistic baseline projections with PROLONG.

Types of uncertainty in technology modeling and how it is addressed in PROLONG

Epistemic uncertainty in technology forecasting arises from incomplete knowledge about diffusion mechanisms and causal relationships. In principle, it could be reduced through additional research, better data, or improved understanding [53].

In technology diffusion modelling, parameter uncertainty [54, 55] emerges from limited historical data about growth rates, market penetration ceilings, and takeoff timings. This uncertainty is particularly strong when technologies are in early deployment phases, when available deployment data provides incomplete information about future patterns. Combined with uncertainty in mathematical representation, parameter and epistemic uncertainty induce *model uncertainty* [56, 57]. With respect to renewables diffusion, model uncertainty manifests in complex and non-linear relationship between early national deployment patterns and eventual global outcomes, with poorly understood functional forms.

Aleatory uncertainty captures inherent randomness or natural variability that remains irreducible regardless of knowledge improvements [53]. In technology diffusion, this arises from genuinely random variations in policies, adoption decisions, and market fluctuations. Both aleatory and epistemic uncertainty is manifested through variations in takeoff timings, growth rates, and saturation levels due to varying policy, resource, and socio-economic contexts. Finally, **deep uncertainty** [58, 59] encompasses fundamental limits to predictability in socio-technical systems creating uncertainties that map neither to epistemic nor aleatory categories. Systemic shocks including wars, pandemics, and economic crises can fundamentally alter deployment trajectories in ways that resist probabilistic characterization and quantitative modelling,

PROLONG engages with these uncertainties in the following ways:

- Epistemic uncertainty is reduced by grounding the model in cross-country empirical regularities, segmenting national growth into formative, accelerating growth, and steady growth phases that reflect underlying diffusion mechanisms.
- Parameter uncertainty is addressed by drawing information from countries at more advanced growth stages and using quantile random forests (Meinshausen 2006) to estimate full conditional distributions without restrictive parametric assumptions.
- Model uncertainty is managed by testing multiple functional forms (logistic, bi-logistic, mixed), ensuring that alternative growth structures are represented.

- Aleatory and epistemic uncertainties together are handled through Monte Carlo simulations: thousands of virtual worlds are generated by drawing parameters from wide distributions (capturing epistemic uncertainty) and assigning them stochastically to countries (capturing aleatory uncertainty). This "virtual laboratory" approach explores a wide range of plausible futures rather than producing projections based on a single historical trajectory.

Uncertainty quantification for solar PV and onshore wind

To test whether PROLONG's probabilistic forecasts are well-calibrated and practically informative, we conduct hindcasting experiments: truncating historical deployment data, generating probabilistic projections, and comparing them to withheld observations. We then assess forecast quality across several complementary metrics, with each metric serving a specific diagnostic purpose.

Interval widths measure how uncertainty evolves over time [51]; narrow intervals provide precision but risk missing observations, while wide intervals ensure coverage but sacrifice informativeness.

Coverage rates test whether our Monte Carlo approach successfully captures the full range of plausible outcomes by checking if actual out-of-sample observations fall within our prediction intervals [49].

Relative uncertainty normalises interval width by the median prediction, indicating whether uncertainty dominates the central estimate. Values above 1.0 suggest uncertainty exceeds the prediction itself, limiting practical utility [51].

Interval scores provide another means of assessing the trade-off between narrow and wide intervals through a penalty-based metric where out-of-sample observations within intervals receive only the interval width as penalty, while missed observations receive additional penalties proportional to the distance outside the interval [52].

Continuous Ranked Probability Score (CRPS) evaluates whether our machine learning approach produces well-calibrated probability distributions rather than just accurate point estimates [49, 50]. We first generate samples that respect the quantile structure of the forecasts and then use them to calculate how well the entire distribution matches reality, with lower values indicating better probabilistic skill. This ensures that PROLONG not only produces good average forecasts but also has reliable uncertainty bounds. CRPS is measured on the same scale as our data (in this case, in fraction in electricity generation).

Tail heaviness characterises the shape of the predictive distribution, i.e. whether forecasts allocate more or less probability to the extremes relative to the central range, distinguishing light- from heavy-tailed forecasts [60].

Together, these metrics provide a comprehensive picture of how successfully PROLONG handles different uncertainty types and the remaining uncertainty over the forecast horizons we can test. Our analysis (Supplementary Figure 8) shows the following:

- **Successful uncertainty capture.** Mean 90% interval widths increase with forecast horizon for both technologies (panel a) but remain modest (<0.08), indicating widening uncertainty that does not explode over time. Coverage rates stay close to the nominal 90% (panel b): Solar PV tracks closely across horizons, while onshore wind shows minor under-coverage at short horizons and over-coverage later. This suggests that Monte Carlo sampling captures a realistic range of plausible outcomes without requiring excessively wide intervals.
- **Appropriate precision-uncertainty balance.** Relative uncertainty (panel c) grows gradually with horizon, reaching around unity (around 1–1.4). This shows that uncertainty becomes comparable to central forecasts over longer horizons—appropriate for long-run energy projections—yet does so in a structured way rather than breaking down erratically. Interval scores (panel d) remain low and closely track interval widths, indicating that strong coverage is achieved through well-calibrated intervals rather than inflated uncertainty bands.
- **Well-calibrated probabilistic skill.** CRPS values are very low (all <0.01; panel e) and increase only gradually with horizon, reflecting the expected rise in difficulty. Because CRPS accounts for the entire predictive distribution, these results confirm that PROLONG generates distributions that are both informative and well-calibrated, not just accurate on average.
- **Extremes are disciplined, not suppressed.** Tail heaviness (panel f) stays between 1.2 and 1.6, lighter than a Gaussian benchmark (approx. 2.1). This indicates that PROLONG forecasts are moderately tailed: they assign probability to extremes, but in a disciplined way. Onshore wind shows increasing tail heaviness with horizon, reflecting growing long-run uncertainty, while Solar PV peaks at mid-horizons before tapering.

Taken together, these diagnostics demonstrate that PROLONG (i) generates uncertainty intervals that expand credibly with horizon, (ii) achieves near-nominal coverage, (iii) maintains low interval scores aligned with widths, (iv) delivers well-calibrated probabilistic distributions (low CRPS),

and (v) allocates moderate, technology-specific weight to extremes. In doing so, PROLONG balances reliability (calibration) with informativeness (sharpness), while avoiding unrealistic fat-tailed forecasts.

The Monte Carlo framework captures both epistemic uncertainty (via parameter distributions) and aleatory uncertainty (via stochastic country assignments), while the machine learning component addresses model uncertainty by learning national–global relationships directly from data. Nonetheless, these evaluations are limited by the historical record: we can only validate hindcasts up to 10 years for solar PV and 15 years for onshore wind. Beyond these horizons, empirical assessment is not yet possible, and future work should revisit coverage, CRPS, and tail dynamics as longer out-of-sample periods become available.

Supplementary Note 6: Acceleration scenarios for onshore wind and solar PV

To illustrate the scale of policy effort required to achieve global deployment trajectories in-line with keeping warming below 1.5°C, we develop two sets of stylised counterfactual trajectories. The first, Early Acceleration trajectories assume that the growth of solar PV and onshore wind continues along a logistic curve with additional policy effort accelerating additions by expanding the deployment ceiling. This is achieved by adjusting the logistic curve ceiling (L) to achieve the desired deployment level in 2040 while keeping the intrinsic growth rate (k) fixed. The second, Late Acceleration trajectories assume that the technologies follow our median projection until 2030 and then experience a fresh pulse of accelerating growth modelled using a second logistic curve with another ceiling and a high intrinsic growth rate, essentially producing a bilogistic trajectory. Here, additional policy effort introduces a new growth pulse which alters both, the effective deployment ceiling as well as the rate of growth acceleration.

These counterfactual scenarios represent distinct policy approaches: Early Acceleration reflects immediate, sustained policy intervention that shifts the technology's long-term trajectory by raising its deployment ceiling and addressing emergent barriers, while Late Acceleration represents a delayed but more intensive intervention that creates a distinct second wave of adoption. By modeling both approaches, we can examine trade-offs between timing and intensity of policy intervention, as well as the feasibility of meeting climate targets through different policy pathways.

These trajectories are compared against Baseline trajectories based on the median probabilistic projections from PROLONG. For onshore wind, this baseline is modelled using a logistic function

with $k = 0.15$ and $L = 28\%$. For solar PV, it is modelled using a logistic function with $k = 0.22$ and $L = 21\%$.

For onshore wind, we model the Early Acceleration trajectory using a logistic function with $k = 0.15$ (matching the Baseline) and an increased ceiling $L = 45\%$ (more than $1.6\times$ higher than the Baseline). We model the Late Acceleration trajectory using a bilogistic function with an initial pulse with $k_1 = 0.15$ and $L_1 = 28\%$ (same as the Baseline), and a second pulse from 2030 with $k_2 = 0.2$ and $L_2 = 12\%$, which gives an effective L $(L_1 + L_2)$ of 40%.

For solar PV, we model the Early Acceleration trajectory using a logistic function with $k = 0.22$ (matching the Baseline) and an increased ceiling $L = 47\%$ (more than $2\times$ higher than the Baseline). We model the Late Acceleration trajectory using a bilogistic function with an initial pulse with $k_1 = 0.22$ and $L_1 = 21\%$ (same as the Baseline), and a second pulse from 2030 with $k_2 = 0.3$ and $L_2 = 26\%$, which gives an effective L $(L_1 + L_2)$ of 47%.

For the Early Acceleration trajectories, we:

1. Start with the baseline projection's parameters (k, G, and L)
2. Maintain the growth rate parameter (k) while increasing the ceiling parameter (L)
3. Recalculate the inflection point (t_0) to ensure the curve passes through the current deployment level using the equation:
$$t_0 = \left(\frac{1}{k} \times \log\left(\frac{L}{y_i} - 1\right)\right) + t_i \tag{7}$$
where y_i is the current deployment level and t_i is the current year
4. Generate the full trajectory using the logistic function:

$$y(t) = \frac{L}{1 + \exp(-k \times (t - t_0))} \tag{8}$$

For Late Acceleration trajectories, we:

1. Take the baseline trajectory up to the second phase start year (2030)
2. Create a second logistic curve with new parameters (k_2, L)
3. Combine the two trajectories using a transition function that shifts from the first to the second curve.

This approach allows us to model the effect of delayed but intensive policy intervention that produces a distinct second wave of technology adoption starting in 2030.

This exercise gives us a set of six global trajectories. For each of these trajectories, we use a linear optimisation approach to distribute the required growth in each year from 2023 to 2050 between 10 regions – East Asia, North America, Europe, South Asia, Asia-Pacific Developed, South-East Asia and Developing Pacific, Africa, Eurasia, Latin America and the Caribbean, and the Middle East. We introduce constraints on the maximum deployment ceiling for each region for each technology, and on the maximum annual growth rate, and the maximum annual growth acceleration.

The maximum deployment ceilings for each technology in each region are adapted from peak deployment in IPCC AR6 scenarios [61], and have been deliberately set higher than what these scenarios consider achievable in some regions to avoid artificially constraining the optimization (Supplementary Table 7).

The algorithm takes as inputs the current deployment levels ($d_{i,t}$) and growth rates ($r_{i,t}$) for each region i at time t, regional weights (w_i) that sum to unity, and a target global deployment trajectory (D_t^*). It incorporates three key constraints that reflect physical and institutional limitations: maximum allowable deployment in each region ($d_{i,\max}$), maximum annual growth rate ($r_{\max}$), and maximum year-on-year acceleration in growth rates ($a_{\max}$).

For each timestep, the algorithm optimizes regional growth rates through an iterative process that converges when the weighted sum of regional deployments matches the global target within a specified tolerance ε:

$$\left| \sum_i (w_i \times d_{i,t}) - D_t^* \right| < \varepsilon \tag{9}$$

The regional deployment in each year is given by:

$$d_{i,t} = d_{i,t-1} + r_{i,t} \tag{10}$$

subject to the constraints:

$$\begin{aligned} 0 \leq d_{i,t} &\leq d_{i,\max} \quad \text{(deployment bounds)} && (11) \\ 0 \leq r_{i,t} &\leq r_{\max} \quad \text{(growth rate bounds)} && (12) \\ -a_{\max} \leq r_{i,t} - r_{i,t-1} &\leq a_{\max} \quad \text{(acceleration bounds)} && (13) \end{aligned}$$

Based on empirical observations of historical technology diffusion rates, we set $r_{\max} = 0.03$ (3 percentage points increase in market share per year) and $a_{\max} = 0.01$ (maximum 1 percentage point

change in annual growth rate). These constraints reflect the practical limitations observed in how quickly technologies can be deployed at regional scales.

For each year, the algorithm first calculates the required global increase in deployment ($\Delta D_t^* = D_t^* - D_{t-1}^*$). It then distributes this increase among regions proportionally to their available headroom ($h_{i,t} = d_{i,\max} - d_{i,t}$). The distribution factor ($f_{i,t}$) for each region is calculated as:

$$f_{i,t} = \frac{h_{i,t}}{\sum_j h_{j,t}} \tag{14}$$

where the sum is over all regions j that have not reached their constraints.

To prevent overshooting the global target, the algorithm implements a conservative adjustment mechanism. When $|\Delta D_t^*| < 0.01$, the algorithm applies a damping factor of 0.5 to the growth rate adjustments. If overshooting occurs ($\sum_i (w_i \times d_{i,t}) > D_t$), the algorithm allows growth rates to decline while maintaining $r_{i,t} \geq 0$ and respecting the acceleration constraint $|r_{i,t} - r_{i,t-1}| \leq a_{\max}$.

The algorithm verifies whether the specified global deployment trajectory is achievable under the given constraints by checking that the weighted sum of maximum regional deployments exceeds the global target:

$$\sum_i (w_i \times d_{i,\max}) \geq D_t^* \quad \forall t \tag{15}$$

If this condition is not met, the algorithm terminates, as the regional constraints preclude achievement of the global target.

This approach allows us to generate regional trajectories (Supplementary Figures 9 and 11, Extended Data Figure 9) that help quantify the regional policy effort required to achieve different global growth trajectories while maintaining consistency with empirically-observed limits on how quickly regions can accelerate technology deployment (Supplementary Figures 10 and 12).

Supplementary Note 7: The case of offshore wind power

Our analysis in this paper focuses on the growth of onshore wind power and excludes offshore wind primarily because the sample of countries for this technology is not sufficiently large. Although offshore wind power shares many fundamental characteristics with its onshore counterpart, it possesses several distinguishing features that warrant separate consideration. Its comparatively lower technological maturity, heightened technical complexity, generally larger turbine unit sizes, unique investment profiles, and specific geophysical requirements all contribute to potentially different temporal and spatial diffusion patterns.

At the global scale, the growth of offshore wind achieved takeoff in 2011 and as of 2023, accounted for roughly 7% of total global installed wind capacity and less than 0.8% of global electricity generation. Unlike onshore wind, its cross-country diffusion has remained rather limited. As of 2023, it participated in electricity generation in 19 countries, of which we have identified take-off in 11 countries – Sweden (2008), Denmark (2010), the UK (2011), Germany (2015), the Netherlands (2016), Finland & South Korea (both 2018), Belgium (2019), China, Portugal and Vietnam (all 2020).

Of these 11, 8 countries have seen deployment levels exceed 1% of annual electricity generation with Denmark (26.1%), the UK (17.5%), the Netherlands (10.1%), Belgium (9%), Germany (5.1%) and Vietnam (1.3%) showing particularly strong growth (Supplementary Figures 13 and 14). Together, these 7 countries are responsible for about 54% of global offshore wind deployment. China leads deployment in absolute terms and contributes to 52% of the global total – this means that over 96% of global offshore wind deployment is concentrated in just 8 countries.

Only Belgium, Denmark, Finland, Germany, South Korea, Sweden and the UK have ≥ 5 datapoints after take-off and are eligible for curve-fitting. As of 2023, only Denmark, Germany and Sweden had exhibited logistic curve maturities over 50%, indicating that growth is still accelerating in most countries. This leaves us with a sample far too small to use with our approach based on using evidence from countries at more mature phases of adoption to inform projections for global growth.

References

[1] Griliches, Z. Hybrid corn: An exploration in the economics of technological change. *Econometrica* **25**, 501 (1957).

[2] Rogers, E. M. *Diffusion of Innovations* 3 edn (The Free Press, New York; United States, 1983).

[3] Grubler, A. *The Rise and Fall of Infrastructures Dynamics of Evolution and Technological Change* (Physica-Verlag Heidelberg, Heidelberg; Germany, 1990). URL https://pure.iiasa.ac.at/id/eprint/3351/.

[4] Kazlou, T., Cherp, A. & Jewell, J. Feasible deployment of carbon capture and storage and the requirements of climate targets. *accepted to Nature Climate Change* (2024).

[5] Brutschin, E., Cherp, A. & Jewell, J. Failing the formative phase: The global diffusion of nuclear power is limited by national markets. *Energy Research & Social Science* **80**, 102221 (2021).

[6] Cherp, A., Vinichenko, V., Tosun, J., Gordon, J. A. & Jewell, J. National growth dynamics of wind and solar power compared to the growth required for global climate targets. *Nature Energy* **6**, 742–754 (2021).

[7] Bento, N., Wilson, C. & Anadon, L. D. Time to get ready: Conceptualizing the temporal and spatial dynamics of formative phases for energy technologies. *Energy Policy* **119**, 282–293 (2018). URL https://doi.org/10.1016/j.enpol.2018.04.015.

[8] Bento, N. & Wilson, C. Measuring the duration of formative phases for energy technologies. *Environmental Innovation and Societal Transitions* **21**, 95–112 (2016). URL http://dx.doi.org/10.1016/j.eist.2016.04.004.

[9] Grubler, A., Wilson, C. & Nemet, G. Apples, oranges, and consistent comparisons of the temporal dynamics of energy transitions. *Energy Research & Social Science* **22**, 18–25 (2016).

[10] Bergek, A., Jacobsson, S., Carlsson, B., Lindmark, S. & Rickne, A. Analyzing the functional dynamics of technological innovation systems: A scheme of analysis. *Research Policy* **37**, 407–429 (2008). URL message:%3CCAPfknAVYNJ2orwYcemUZECisCE_AS5mZhGcgJN4i7SiyiTgt-Q@mail.gmail.com%3E.

[11] Jacobsson, S. & Bergek, A. Transforming the energy sector: the evolution of technological systems in renewable energy technology. *Industrial and Corporate Change* **13**, 815–849 (2004).

[12] Markard, J. The next phase of the energy transition and its implications for research and policy. *Nature Energy* **3**, 628–633 (2018). URL http://dx.doi.org/10.1038/s41560-018-0171-7. First, policy support for now mature technologies such as wind and solar is likely to decrease (sic!).

[13] Jacobsson, S. & Lauber, V. The politics and policy of energy system transformation—explaining the german diffusion of renewable energy technology. *Energy Policy* **34**, 256–276 (2006).

[14] Gosens, J., Hedenus, F. & Sandén, B. A. Faster market growth of wind and PV in late adopters due to global experience build-up. *Energy* **131**, 267–278 (2017).

[15] Vinichenko, V., Jewell, J., Jacobsson, J. & Cherp, A. Historical diffusion of nuclear, wind and solar power in different national contexts: implications for climate mitigation pathways. *Environmental Research Letters* **18** (2023).

[16] Malhotra, A. & Schmidt, T. S. Accelerating low-carbon innovation. *Joule* **4**, 2259–2267 (2020).

[17] Wilson, C. *et al.* Granular technologies to accelerate decarbonization. *Science* **368**, 36–39 (2020).

[18] Pierson, P. Increasing returns, path dependence, and the study of politics. *American Political Science Review* **94**, 251–267 (2000).

[19] Jacobsson, S. & Johnson, A. The diffusion of renewable energy technology: an analytical framework and key issues for research. *Energy Policy* **28**, 625–640 (2000).

[20] Ioannidis, R. & Koutsoyiannis, D. A review of land use, visibility and public perception of renewable energy in the context of landscape impact. *Applied Energy* **276**, 115367 (2020).

[21] Frantál, B., Frolova, M. & Liñán-Chacón, J. Conceptualizing the patterns of land use conflicts in wind energy development: Towards a typology and implications for practice. *Energy Research & Social Science* **95**, 102907 (2023).

[22] Mulvaney, D. Identifying the roots of green civil war over utility-scale solar energy projects on public lands across the american southwest. *Journal of Land Use Science* **12**, 493–515 (2017).

[23] Stokes, L. C., Franzblau, E., Lovering, J. R. & Miljanich, C. Prevalence and predictors of wind energy opposition in north america. *Proceedings of the National Academy of Sciences* **120**, e2302313120 (2023).

[24] Ko, I. Rural opposition to landscape change from solar energy: Explaining the diffusion of setback restrictions on solar farms across south korean counties. *Energy Research & Social Science* **99**, 103073 (2023).

[25] Crawford, J., Bessette, D. & Mills, S. B. Rallying the anti-crowd: Organized opposition, democratic deficit, and a potential social gap in large-scale solar energy. *Energy Research & Social Science* **90**, 102597 (2022).

[26] Patterson, J. J. Backlash to climate policy. *Global Environmental Politics* **23**, 68–90 (2023).

[27] Breetz, H., Mildenberger, M. & Stokes, L. The political logics of clean energy transitions. *Business and Politics* **20**, 492–522 (2018).

[28] Stokes, L. C. & Breetz, H. L. Politics in the u.s. energy transition: Case studies of solar, wind, biofuels and electric vehicles policy. *Energy Policy* **113**, 76–86 (2018).

[29] Stokes, L. C. Electoral backlash against climate policy: A natural experiment on retrospective voting and local resistance to public policy. *American Journal of Political Science* **60**, 958–974 (2016).

[30] Gorman, W. *et al.* Grid connection barriers to renewable energy deployment in the united states. *Joule* **9**, 101791 (2025).

[31] Johnston, S., Liu, Y. & Yang, C. *An Empirical Analysis of the Interconnection Queue* Working Paper Series (National Bureau of Economic Research, Cambridge; United States, 2023). URL http://www.nber.org/papers/w31946.

[32] Heptonstall, P. J. & Gross, R. J. K. A systematic review of the costs and impacts of integrating variable renewables into power grids. *Nature Energy* **6**, 72–83 (2021).

[33] Martinot, E. Grid integration of renewable energy: Flexibility, innovation, and experience. *Annual Review of Environment and Resources* **41**, 223–251 (2016).

[34] Markard, J., Bento, N., Kittner, N. & Nuñez-Jimenez, A. Destined for decline? examining nuclear energy from a technological innovation systems perspective. *Energy Research & Social Science* **67**, 101512 (2020).

[35] Verhulst, P. Recherches mathématiques sur la loi d'accroissement de la population. *Nouveaux Mémoires de l'Académie Royale des Sciences et Belles-Lettres de Bruxelles* **18**, 1–45 (1845).

[36] Wilson, C., Grubler, A., Bauer, N., Krey, V. & Riahi, K. Future capacity growth of energy technologies: are scenarios consistent with historical evidence? *Climatic Change* **118**, 381–395 (2013).

[37] Debecker, A. & Modis, T. Determination of the uncertainties in s-curve logistic fits. *Technological Forecasting and Social Change* **46**, 153–173 (1994). Estimate the level of maturity of the logistic curve which is necessary to reliably estimate K from empirical data.

[38] Odenweller, A., Ueckerdt, F., Nemet, G. F., Jensterle, M. & Luderer, G. Probabilistic feasibility space of scaling up green hydrogen supply. *Nature Energy* **7**, 854–865 (2022).

[39] Grubb, M., Drummond, P. & Hughes, N. *The shape and pace of change in the electricity transition* (We Mean Business Coalition, Washington, DC; USA, 2020). URL https://www.wemeanbusinesscoalition.org/wp-content/uploads/2020/10/Shape-and-Pace-of-Change-in-the-Electricity-Transition-1.pdf.

[40] Dixon, R. Hybrid corn revisited. *Econometrica* **48**, 1451 (1980).

[41] Gompertz, B. On the nature of the function expressive of the law of human mortality and on a new mode of determining the value of life contingencies. *Phil.Trans.Roy.Soc.* **123**, 513–585 (1825).

[42] Haegel, N. M. *et al.* Photovoltaics at multi-terawatt scale: Waiting is not an option. *Science* **380**, 39–42 (2023).

[43] Victoria, M. *et al.* Solar photovoltaics is ready to power a sustainable future. *Joule* **5**, 1041–1056 (2021).

[44] Creutzig, F. *et al.* The underestimated potential of solar energy to mitigate climate change. *Nature Energy* **2**, 17140 (2017).

[45] Way, R., Ives, M. C., Mealy, P. & Farmer, J. D. Empirically grounded technology forecasts and the energy transition. *Joule* **6**, 2057–2082 (2022).

[46] Meyer, P. Bi-logistic growth. *Technological Forecasting and Social Change* **47**, 89–102 (1994).

[47] Zielonka, N. & Trutnevyte, E. Probabilities of reaching required diffusion of granular energy technologies in european countries. *iScience* **28**, 111825 (2025).

[48] Zielonka, N., Wen, X. & Trutnevyte, E. Probabilistic projections of granular energy technology diffusion at subnational level. *PNAS Nexus* **2**, pgad321 (2023).

[49] Gneiting, T. & Raftery, A. E. Strictly proper scoring rules, prediction, and estimation. *Journal of the American Statistical Association* **102**, 359–378 (2007).

[50] Price, I. *et al.* Probabilistic weather forecasting with machine learning. *Nature* **637**, 84–90 (2025).

[51] Gneiting, T. & Katzfuss, M. Probabilistic forecasting. *Annual Review of Statistics and Its Application* **1**, 125–151 (2014).

[52] Bracher, J., Ray, E. L., Gneiting, T. & Reich, N. G. Evaluating epidemic forecasts in an interval format. *PLoS Computational Biology* **17**, e1008618 (2021).

[53] Hüllermeier, E. & Waegeman, W. Aleatoric and epistemic uncertainty in machine learning: an introduction to concepts and methods. *Machine Learning* **110**, 457–506 (2021).

[54] Spall, J. C. Modeling uncertainty, an examination of stochastic theory, methods, and applications. *International Series in Operations Research Management Science* 685–709 (2002).

[55] Petropoulos, F., Hyndman, R. J. & Bergmeir, C. Exploring the sources of uncertainty: Why does bagging for time series forecasting work? *European Journal of Operational Research* **268**, 545–554 (2018).

[56] Draper, D. Assessment and propagation of model uncertainty. *Journal of the Royal Statistical Society: Series B (Methodological)* **57**, 45–70 (1995).

[57] Cai, Y. & Sanstad, A. Model uncertainty and energy technology policy: The example of induced technical change. *SSRN Electronic Journal* (2014).

[58] Walker, W. E. *et al.* Defining uncertainty: A conceptual basis for uncertainty management in model-based decision support. *Integrated Assessment* **4**, 5–17 (2003).

[59] Haas, C., Kempa, K. & Moslener, U. Dealing with deep uncertainty in the energy transition: What we can learn from the electricity and transportation sectors. *Energy Policy* **179**, 113632 (2023).

[60] Tian, H., Yim, A. & Newton, D. P. Tail-heaviness, asymmetry, and profitability forecasting by quantile regression. *Management Science* **67**, 5209–5233 (2021).

[61] Byers, E. *et al.* AR6 scenarios database (2022). URL https://doi.org/10.5281/zenodo.5886912.
